# Skeletal muscle atrophy in clinical and preclinical models of chronic kidney disease: A systematic review and meta‐analysis

**DOI:** 10.1002/jcsm.13400

**Published:** 2023-12-07

**Authors:** Ashley D. Troutman, Eliott Arroyo, Elizabeth M. Sheridan, Duncan J. D'Amico, Peyton R. Brandt, Rachel Hinrichs, Xiwei Chen, Kenneth Lim, Keith G. Avin

**Affiliations:** ^1^ Department of Physical Therapy, School of Health and Human Sciences Indiana University Purdue University Indianapolis Indiana USA; ^2^ Department of Medicine, Division of Nephrology & Hypertension Indiana University School of Medicine Indianapolis Indiana USA; ^3^ University Library Indiana University‐Purdue University Indianapolis Indiana USA; ^4^ Department of Epidemiology and Biostatistics, School of Public Health Indiana University Bloomington Bloomington Indiana USA

**Keywords:** Sarcopenia, Atrophy, Skeletal muscle, Chronic kidney disease, sex‐differences, dialysis

## Abstract

Patients with chronic kidney disease (CKD) are often regarded as experiencing wasting of muscle mass and declining muscle strength and function, collectively termed sarcopenia. The extent of skeletal muscle wasting in clinical and preclinical CKD populations is unclear. We evaluated skeletal muscle atrophy in preclinical and clinical models of CKD, with multiple sub‐analyses for muscle mass assessment methods, CKD severity, sex and across the different preclinical models of CKD. We performed a systematic literature review of clinical and preclinical studies that measured muscle mass/size using the following databases: Ovid Medline, Embase and Scopus. A random effects meta‐analysis was utilized to determine standard mean difference (SMD; Hedges' g) between healthy and CKD. Heterogeneity was evaluated using the *I* ^2^ statistic. Preclinical study quality was assessed via the Systematic Review Centre for Laboratory Animal Experimentation and clinical studies quality was assessed via the Newcastle‐Ottawa Scale. This study was registered in PROSPERO (CRD42020180737) prior to initiation of the search. A total of 111 studies were included in this analysis using the following subgroups: 106 studies in the primary CKD analysis, 18 studies that accounted for diabetes and 7 kidney transplant studies. Significant atrophy was demonstrated in 78% of the preclinical studies and 49% of the clinical studies. The random effects model demonstrated a medium overall SMD (SMD = 0.58, 95% CI = 0.52–0.64) when combining clinical and preclinical studies, a medium SMD for the clinical population (SMD = 0.48, 95% CI = 0.42–0.55; all stages) and a large SMD for preclinical CKD (SMD = 0.95, 95% CI = 0.76–1.14). Further sub‐analyses were performed based upon assessment methods, disease status and animal model. Muscle atrophy was reported in 49% of the clinical studies, paired with small mean differences. Preclinical studies reported significant atrophy in 78% of studies, with large mean differences. Across multiple clinical sub‐analyses such as severity of CKD, dialysis modality and diabetes, a medium mean difference was found. Sub‐analyses in both clinical and preclinical studies found a large mean difference for males and medium for females suggesting sex‐specific implications. Muscle atrophy differences varied based upon assessment method for clinical and preclinical studies. Limitations in study design prevented conclusions to be made about the extent of muscle loss with disease progression, or the impact of dialysis. Future work would benefit from the use of standardized measurement methods and consistent clinical staging to improve our understanding of atrophy changes in CKD progression, and analysis of biological sex differences.

## Introduction

Chronic kidney disease (CKD) is a progressive disease with an estimated global prevalence of 13% (11.7–15.1%)^1^ [S1]. Patients with CKD often present with a combination of decreased muscle mass and muscle strength and function which is defined as sarcopenia.^2^ The aetiology of sarcopenia in CKD is complex and multifactorial, with CKD‐related comorbidities (e.g., diabetes, obesity, cardiovascular disease), complications (e.g., uraemia, metabolic acidosis, inflammation, oxidative stress, Klotho deficiency) and therapies (e.g., dialysis) all impacting skeletal muscle wasting.^2^, ^3^, ^4^, ^5^ Together, these factors can increase protein degradation and decrease muscle protein synthesis, leading to a negative protein balance, impaired muscle regeneration and muscle atrophy^2^, ^3^, ^4^ [S2]. CKD‐related skeletal muscle wasting contributes to reductions in muscle strength and physical function,^6^ which increases the risk of bone fractures,^7^ worsens quality of life [S3] and increases mortality [S4]. The magnitude or severity of skeletal muscle atrophy associated with CKD remains unclear due to inconsistency in published literature for both clinical and preclinical models of CKD.

One of the challenges determining the extent of muscle atrophy in CKD is the wide variety of methods used to quantify muscle mass in published research studies. These methods include anthropometry, muscle biopsy, bioelectrical impedance analysis (BIA), dual energy X‐ray absorptiometry (DEXA), computed tomography (CT) and magnetic resonance imaging (MRI).^8^ These techniques vary widely in accuracy and reliability to assess skeletal muscle mass, such as total lean body mass, appendicular lean mass, muscle cross‐sectional area and myofibre cross‐sectional area.^9^ The accuracy of all methods for assessing muscle mass can be impacted by CKD‐related factors. For instance, fluctuations in hydration status may impede a proper assessment of muscle mass via BIA or DEXA.^8^ Together, the use of various techniques, measurements, equations and algorithms used to estimate skeletal muscle mass make it challenging to determine the magnitude of skeletal muscle atrophy in CKD.

Several preclinical models of CKD have been developed to investigate the pathophysiology of CKD and to evaluate the efficacy of new therapeutic approaches for CKD‐associated pathologies.^10^, ^11^, ^12^ These animal models closely mimic human CKD progression with decreased glomerular filtration rate (GFR) and uraemia.^11^, ^12^, ^13^ It is unclear from the published literature if skeletal muscle atrophy in preclinical models mimics the clinical CKD presentation. The aims of this meta‐analysis are to (1) evaluate the magnitude of skeletal muscle atrophy in both preclinical and clinical models of CKD and (2) to determine if skeletal muscle atrophy differs among the various methodologies used to assess skeletal muscle mass, across the various stages of CKD, across the different preclinical models of CKD and sex.

## Methods

### Literature search strategy and screen

The literature search utilized liberal search terms surrounding muscle atrophy to identify all potentially relevant studies of CKD and skeletal muscle. The intent of the search was not to identify effectiveness of interventions, but rather to identify the magnitude of skeletal muscle atrophy in clinical and preclinical CKD studies. Literature searches were originally performed on 17 February 2020 and then updated on 13 September 2021 in the following databases: Medline (Ovid), Embase (Elsevier) and Scopus (Elsevier) from 1946 to 2021. Search terms for CKD included kidney disease, renal insufficiency, renal replacement therapy, dialysis and kidney/renal failure. Search terms for skeletal muscle included muscular atrophy, sarcopenia and lean body mass as indicated in the supporting information or via hyperlinks. See the full search strategies for all databases in searchRxiv. The search was limited to English articles, and editorials, letters, news and commentaries were removed. Results were de‐duplicated and screened using Covidence systematic review software (Veritas Health Innovation, Melbourne, Australia). Studies identified in the search process were screened by nine reviewers in total, where each study was assessed by two reviewers. In the event of disagreement between two reviewers, a third reviewer served as a mediator to determine if a study should be included or excluded. Virtual meetings were held as needed in the event of a disagreement to clarify the screening process by discussing rationale for inclusion/exclusion (Figure S1). This study was registered in PROSPERO (CRD42020180737) prior to initiation of the search.

### Inclusion and exclusion criteria

The inclusion criteria included the following: (1) a study sample consisting of adults aged 18 and older with CKD stages III–IV or end‐stage kidney disease (ESKD; CKD stage 5 and/or 5d, the latter denotes those on dialysis), or CKD animal models of all relevant species (i.e., mouse, rat and rabbit) regardless of the underlying CKD aetiology; and (2) studies that included a measure of muscle mass/size such as: DEXA, BIA [i.e., single frequency‐BIA (SF‐BIA) and multifrequency bioimpedance spectroscopy (MF‐BIS)], MRI, ultrasound, histological assessment and/or anthropometry (i.e., circumferential measurement estimated body composition). Interventional studies that assessed muscle mass were included with only pre‐intervention values in the analyses. Studies with additional co‐morbidities were excluded except for diabetes which was included in the primary analysis and sub‐analyses. Kidney transplant recipient studies were only included as a sub‐analysis. To perform sub‐analyses, we determined *a priori* three studies needed to be published. As an example, if a particular animal model had less than three studies the data would be included in the overall preclinical analyses, but a sub‐analysis was not performed. Studies categorized as grey literature or prevalence studies were not considered in this analysis. Studies were excluded if authors were not able to obtain the full paper despite request (abstract only), if the study did not have the correct outcomes or patient population, if a healthy control was not referenced, if a study was duplicated, retracted or if the data were incompatible for our analysis (correlations).

### Outcome variables

Muscle atrophy was assessed in studies that compared group (healthy controls vs. CKD) differences for a measure of muscle size or mass. Muscle assessments may be performed indirectly through measures such as BIA (i.e., total body lean mass) or directly through histological assessment (i.e., muscle fibre cross‐sectional area). In studies with multiple measures, all measures were included to provide muscle mass/size changes from micro to macro, each valuable in identifying muscle size depending upon the research environment and target population. Additional sub‐analyses include the following categorical variables to account for assessment method or model heterogeneity: clinical CKD stage (stages III–IV and ESKD), dialysis status (haemodialysis (HD) and peritoneal dialysis (PD)), kidney transplant recipients, diabetes status, muscle size assessment method, muscle fibre type and preclinical CKD model (i.e., 5/6 nephrectomy, Cy/+, adenine‐induced).

### Data extraction and compilation

Data extraction and translation were performed independently by two authors per study to ensure proper data translation. Relevant data reported in figure form only, required data extraction by transforming numerical scale values from pixels using Photoshop (Adobe Photoshop, San Jose, CA). Data presented with standard error or confidence interval were converted to standard deviation using standard equations [S5]. All relevant data were compiled in an Excel database when available including study information (author, date), human/animal, sample size, sex (male, female, or mixed), mean age, muscle assessment technique, mean and standard deviation (SD) muscle mass; all conditions meeting the inclusion criteria were recorded.

### Quality analysis

Quality analysis was completed for preclinical studies using the Systematic Review Centre for Laboratory Animal Experimentation's (SYRCLE) and clinical studies were evaluated using the Newcastle‐Ottawa Scale (NOS) [S6, S7]. The use of these two assessments enabled the determination of bias, study selection and certainty of evidence. These assessments were utilized by two independent reviewers, with a third reviewer to determine consensus in the event of disagreement. The SYRCLE scale utilizes 10 domains to assign studies as low, high and unclear risk [S8]. Domains with a singular question were categorized by the risk level response. Domains that included multiple responses were categorized as follows. The baseline characteristic domain required two “yes” responses for low risk, varying inconsistent responses to the three questions with at least one “unclear” was categorized as an unclear risk). Random housing and detection domains were considered low risk with at least one ‘yes’ response, while two “unclear” responses resulted in an unclear risk. The attrition domain included four questions. If the first question response was answered ‘yes or unclear’, then the domain was reported as such. If question one was answered ‘no’, then the domain category was based upon questions two through four. If there was at least one ‘no’ response then the domain was categorized as high risk; two ‘yes’ responses resulted in low risk, and two “unclear” responses resulted in unclear risk. The other sources of bias domain included five questions. The domain was considered low risk if at least three of the questions were answered ‘yes’, unclear risk if the domain had less than three ‘yes’ and at least two ‘unclear’ responses, while greater than two ‘no’ responses resulted in high risk. Table S5 provides a detailed description of the SYRCLE's tool. The NOS scale assigns categories as good, fair and poor quality [S9]. Good quality was defined as three to four stars in the selection domain, two in the comparability domain and two to three stars in the outcome/exposure domain. Fair quality was defined as one to two stars in two of the three domains. Poor quality was defined as one star or less for selection or outcome exposure domains or none in the comparability domain. A more detailed explanation of the individual components of NOS are in Table S3 [clinical studies (excluding transplant)] and Table S4 (transplant studies).

### Statistical analyses

Effect size is the standardized mean difference (SMD) between two populations (i.e., healthy vs. CKD) and will be referred to as SMD in this paper [S10]. Hedges' g was chosen as the best SMD estimate due to its correction for overestimations that may occur with small samples [S11]. Effect sizes were categorized as small (0.2–0.49), medium (0.5–0.79) and large (≥0.80) [S12]. Heterogeneity estimates were evaluated at each level of analysis using Comprehensive Meta Analysis V2 software and deemed significant if *I* ^2^ > 50 [S13]. Pooled SMD (and associated variances) across studies were calculated using both random and fixed effects models so a model could be chosen a priori [S14]. A random‐model approach was chosen under the guise of generalizing results among the older population and inherent inequality of effect sizes across studies [S15]. Results are presented such that positive effect size values indicate the presence of muscle atrophy in CKD, whereas negative effect size values indicate greater muscle size in CKD; in figures this is labelled as atrophy and no atrophy, respectively. We present the results graphically using forest plots and include the percentage of studies with a *P*‐value <0.05, indicating significant atrophy in CKD.

## Results

Among the 6987 studies initially identified in the search, 825 studies underwent full text screening, of those 714 were excluded and 111 were included (Figure S1). Studies that met the inclusion criteria were categorized based upon the pre‐specified criteria (Table 1). Baseline characteristics of all the studies can be found in Tables S1 (clinical studies) and S2 (preclinical studies). Skeletal muscle atrophy significance (*P*‐value <0.05) was inconsistently reported when comparing CKD to control groups. Among the 106 total studies included in the main analysis, 78% of the preclinical studies and 49% of the clinical studies reported significant *P*‐values indicating atrophy in CKD. More than one muscle size assessment method was used in 70 studies, 22 of these studies lacked agreement between assessment methods for the presence of significant muscle atrophy. To provide an overview on the extent of skeletal muscle atrophy the random effects model demonstrated a medium overall SMD (SMD = 0.58, 95% CI = 0.52–0.64, *I* ^2^ = 62.46) when combining all clinical and preclinical studies.

**Table 1:** Study characteristics of clinical studies,^6^, ^25^, ^36^, ^37^, ^38^, ^39^, ^40^, ^41^, ^42^, ^43^, ^44^, ^45^, ^46^, ^47^, ^48^, ^49^, ^50^, ^51^, ^52^, ^53^, ^54^, ^55^, ^56^, ^57^, ^58^, ^59^, ^60^, ^61^, ^62^, ^63^, ^64^, ^65^, ^66^, ^67^, ^68^, ^69^, ^70^, ^71^, ^72^, ^73^, ^74^, ^75^, ^76^, ^77^, ^78^, ^79^, ^80^, ^81^, ^82^, ^83^, ^84^, ^85^, ^86^ [S16–38] and preclinical studies^13^, ^27^, ^28^, ^87^, ^88^, ^89^, ^90^, ^91^, ^92^, ^93^, ^94^, ^95^, ^96^, ^97^, ^98^, ^99^, ^100^, ^101^, ^102^, ^103^, ^104^, ^105^, ^106^, ^107^, ^108^, ^109^, ^110^, ^111^, ^112^, ^113^, ^114^, ^115^, ^116^, ^117^, ^118^, ^119^, ^120^

	Studies (*n*)	Participants (*n*)	Studies reporting significant atrophy, *n* (%)
Clinical CKD analysis (overall)			
Clinical (total) CKD	70	35 775	33 (47)
Stages III–IV	5	27 064	3 (60)
ESKD with or without dialysis	58	5351	28 (48)
ESKD without dialysis	5	755	1 (20)
Dialysis (total)	54	4657	27 (50)
Haemodialysis	44	3845	25 (57)
Peritoneal dialysis	11	702	2 (18)
Muscle biopsy	7	423	4 (57)
MRI	4	125	3 (75)
Ultrasound	3	583	2 (67)
DEXA	24	3891	7 (29)
BIA	21	29 180	13 (62)
Anthropometry	13	1166	7 (30)
Mean cross‐sectional area	3	140	3 (100)
Type I cross‐sectional area	7	141	2 (29)
Type II cross‐sectional area	7	282	3 (43)
Females	8	14 485	5 (63)
Males	14	12 924	9 (64)
Clinical sub‐analysis			
Diabetes excluded	18	1903	8 (44)
Transplant^a^	7	541	2 (29)
Preclinical CKD analysis (overall)			
Preclinical (total) CKD	37	1101	28 (76)
Adenine	8	269	6 (75)
5/6th nephrectomy	26	728	22 (85)
Mean CSA	22	274	17 (77)
Muscle weight	26	553	22 (85)
Type II cross‐sectional area	3	242	2 (67)
Type I cross‐sectional area	3	90	2 (67)
Females	5	87	3 (60)
Males	32	945	27 (84)

^a^ Sub‐analyses were not included in the primary CKD analysis.

### Muscle atrophy in chronic kidney disease: Clinical study overview

**Figure 1:**
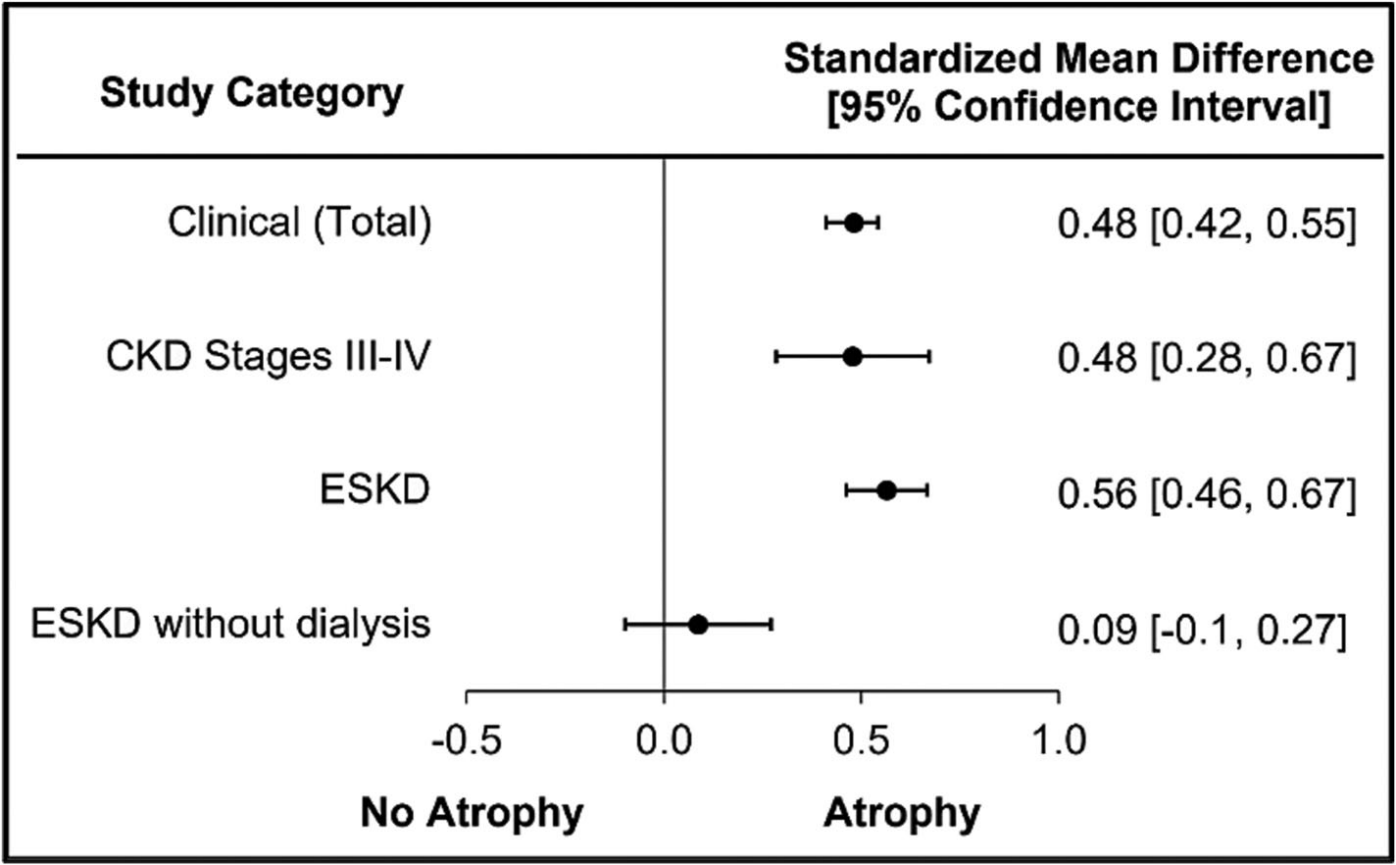
Muscle atrophy is similar across the different stages of CKD; however, patients with ESKD who are not receiving dialysis do not present with significant muscle atrophy.

A total of 34 out of 70 (49%) clinical studies reported atrophy in patients with CKD. To determine if disease progression impacted muscle atrophy, clinical studies were intended to be parcelled out by stage. The CKD literature did not report muscle size measurements by individual CKD stages, with some studies combining stages III–IV or stages II–III as one group. Data were then parcelled out by stages III–IV and ESKD (Figure 1); stages II–III did not meet the required threshold of three studies. A small significant difference was found in the total clinical population (SMD = 0.48, 95% CI = 0.42–0.55, *I* ^2^ = 59.06; all stages; Figure 1). CKD stages III–IV studies demonstrated a small significant difference (SMD = 0.48, 95% CI = 0.28–0.67, *I* ^2^ = 78.23; Figure 1 and Figure S2) and ESKD studies demonstrated a medium significant difference (SMD = 0.56, 95% CI = 0.46–0.67, *I* ^2^ = 24.45; Figure 1). The ESKD without dialysis sub‐analysis demonstrated a lack of muscle atrophy (SMD = 0.09, 95% CI = −0.10 to 0.27, *I* ^2^ = 0.00; Figure 1 and Figure S3). A sub‐analysis of dialysis modalties is presented in the subsequent muscle atrophy in dialysis.

### Muscle atrophy in dialysis

**Figure 2:**
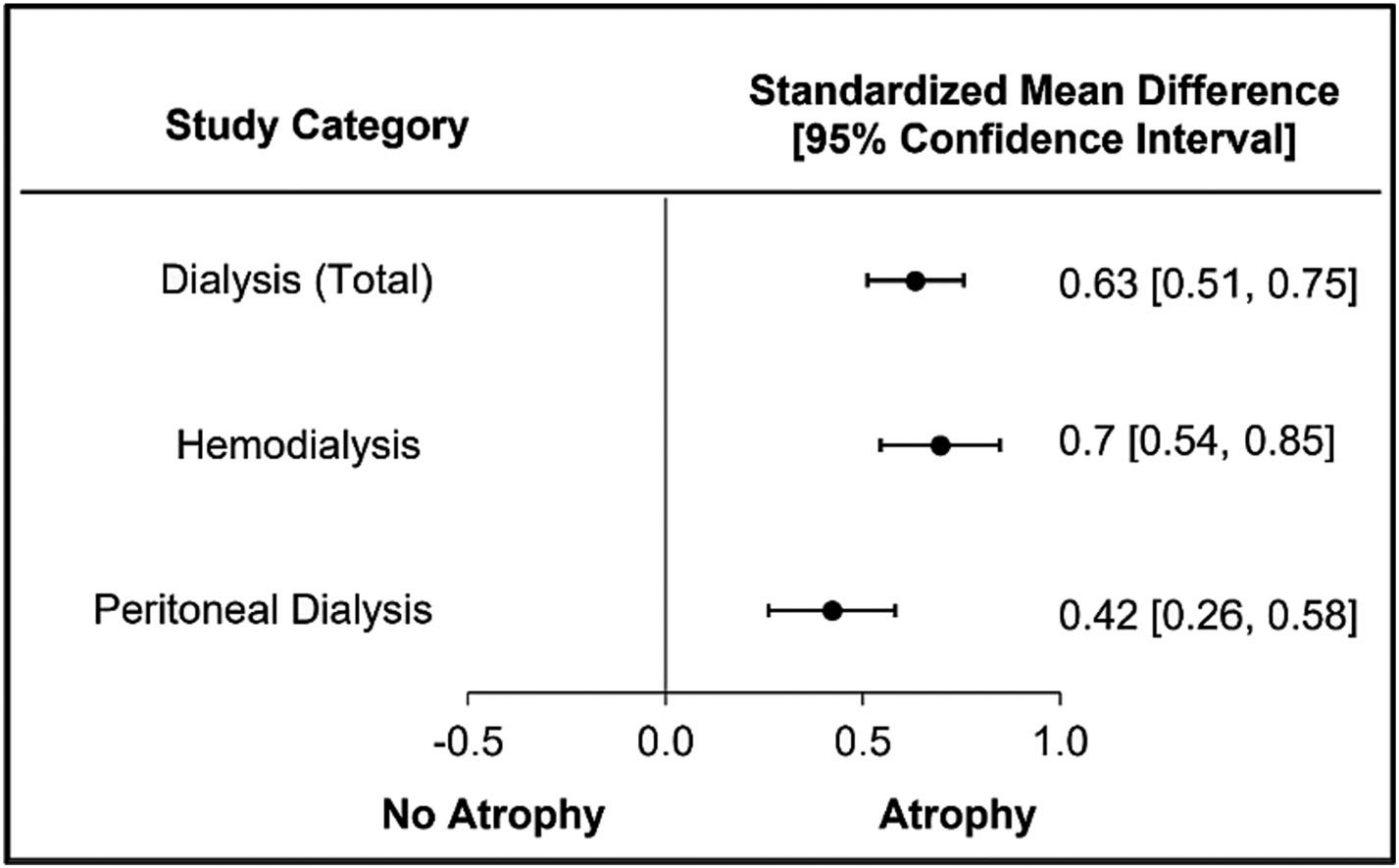
Muscle atrophy is significant in patients regardless of dialysis intake.

Twenty‐eight of the 54 (52%) studies reported atrophy in patients receiving dialysis regardless of type [i.e., HD, PD, not specified (HD&PD combined)]. The total dialysis category includes HD, PD and the unspecified/combined groups; HD and PD were separated for further analysis. Muscle atrophy was demonstrated in the total dialysis sub‐analysis with a medium significant difference (SMD = 0.63, 95% CI = 0.51–0.75, *I* ^2^ = 34.78; Figure 2). Twenty‐five out of 44 (57%) studies reported atrophy in patients receiving HD and three out of 11 studies (27%) reported atrophy in patients receiving PD. HD studies demonstrated a medium significant difference for muscle atrophy (SMD = 0.70, 95% CI = 0.54–0.85, *I* ^2^ = 40.24; Figure 2 and Figure S4), while PD studies demonstrated a small significant difference for muscle atrophy (SMD = 0.42, 95% CI = 0.26–0.58, *I* ^2^ = 0.00; Figure 2 and Figure S5).

### Assessment methods of muscle atrophy: Clinical studies

**Figure 3:**
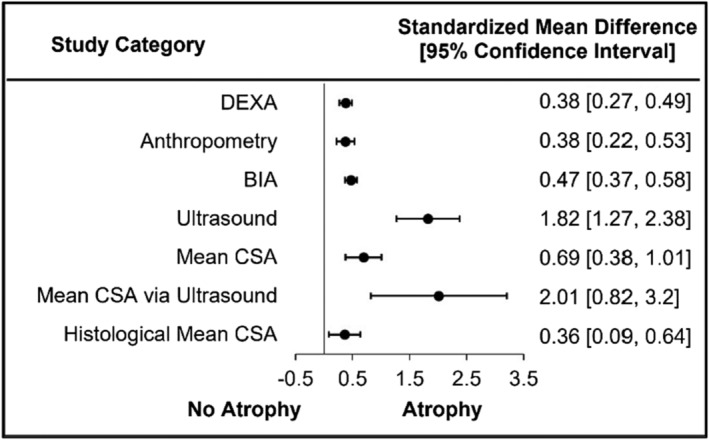
Muscle atrophy is significant in clinical measurements of skeletal muscle atrophy.

Clinical studies assessed muscle atrophy via multiple methods including muscle thickness via ultrasound (100% of three studies demonstrating atrophy), CSA (70% of 10 studies demonstrating atrophy) and BIA (62% of 21 studies demonstrating atrophy). These studies consistently demonstrated muscle atrophy in 60–100% of the studies, while DEXA (29% of 24 studies demonstrating atrophy) and anthropometry (30% of 13 studies demonstrating atrophy) were less consistent. In congruence with *P*‐value reporting, small significant differences were demonstrated for DEXA (SMD = 0.38, 95% CI = 0.27–0.49, *I* ^2^ = 10.47; Figure 3 and Figure S6) and anthropometry (SMD = 0.38, 95% CI = 0.22–0.53, *I* ^2^ = 0.95; Figure 3 and Figure S7), while a medium significant difference was found for BIA (SMD = 0.47, 95% CI = 0.37–0.58, *I* ^2^ = 72.99; Figure 3 and Figure S8) and a large difference for US (SMD = 1.82, 95% CI = 1.27–2.38, *I* ^2^ = 70.02; Figure 3 and Figure S9). Cross‐sectional area was determined via MRI (three studies), or CT (one study) which provided mean CSA or histological assessment (seven studies) which assessed CSA by fibre type (i.e., type I and type II); no histological study provided a mean CSA without fibre‐specific staining. A medium significant difference was found for mean CSA overall (SMD = 0.69, 95% CI = 0.38–1.01, *I* ^2^ = 35.87; Figure 3 and Figure S10), regardless of assessment method. Magnetic resonance imaging demonstrated significant muscle atrophy in all three studies with a large significant difference (SMD = 2.01, 95% CI = 0.82–3.20, *I* ^2^ = 32.56; Figure 3 and Figure S11). Histological assessment studies reported significant atrophy when collapsing fibre types in four of seven studies (57%) that resulted in a small significant difference (SMD = 0.36, 95% CI = 0.09–0.64, *I* ^2^ = 13.68; Figure 3 and Figure S12). Clinical and preclinical fibre‐type specific effect sizes are further disscussed in section assessment of atrophy in muscle fibre types.

### Muscle atrophy in chronic kidney disease with the specific exclusion of diabetes

Diabetes sub‐analyses were restricted due to a lack of properly controlled comparisons between a diabetes/CKD group compared with normal healthy controls or CKD without the presence of disease. The only permissible sub‐analysis was CKD alone (i.e., diabetes excluded) compared with a heterogeneous group of studies with varied percentages CKD participants with diabetes. CKD alone (i.e., diabetes excluded) studies reported significant atrophy in 8 of 18 studies (44%) with a medium significant difference (SMD = 0.56, 95% CI = 0.44–0.67, *I* ^2^ = 24.40; Figure S13). Heterogeneous studies of CKD and diabetes reported significant atrophy in 24 of the 52 studies (46%) with a small significant difference (SMD = 0.44, 95% CI = 0.36–0.52, *I* ^2^ = 69.70; Figure S13).

### Muscle atrophy in kidney transplant recipients

Kidney transplant studies performed muscle size assessment 1 month to 9 years post‐transplant. Muscle atrophy was reported in two of the seven (29%) kidney transplant studies with a small significant difference (SMD = 0.25, 95% CI = 0.06–0.4, *I* ^2^ = 27.03; Figure S14).

### Muscle atrophy in preclinical models of chronic kidney disease

**Figure 4:**
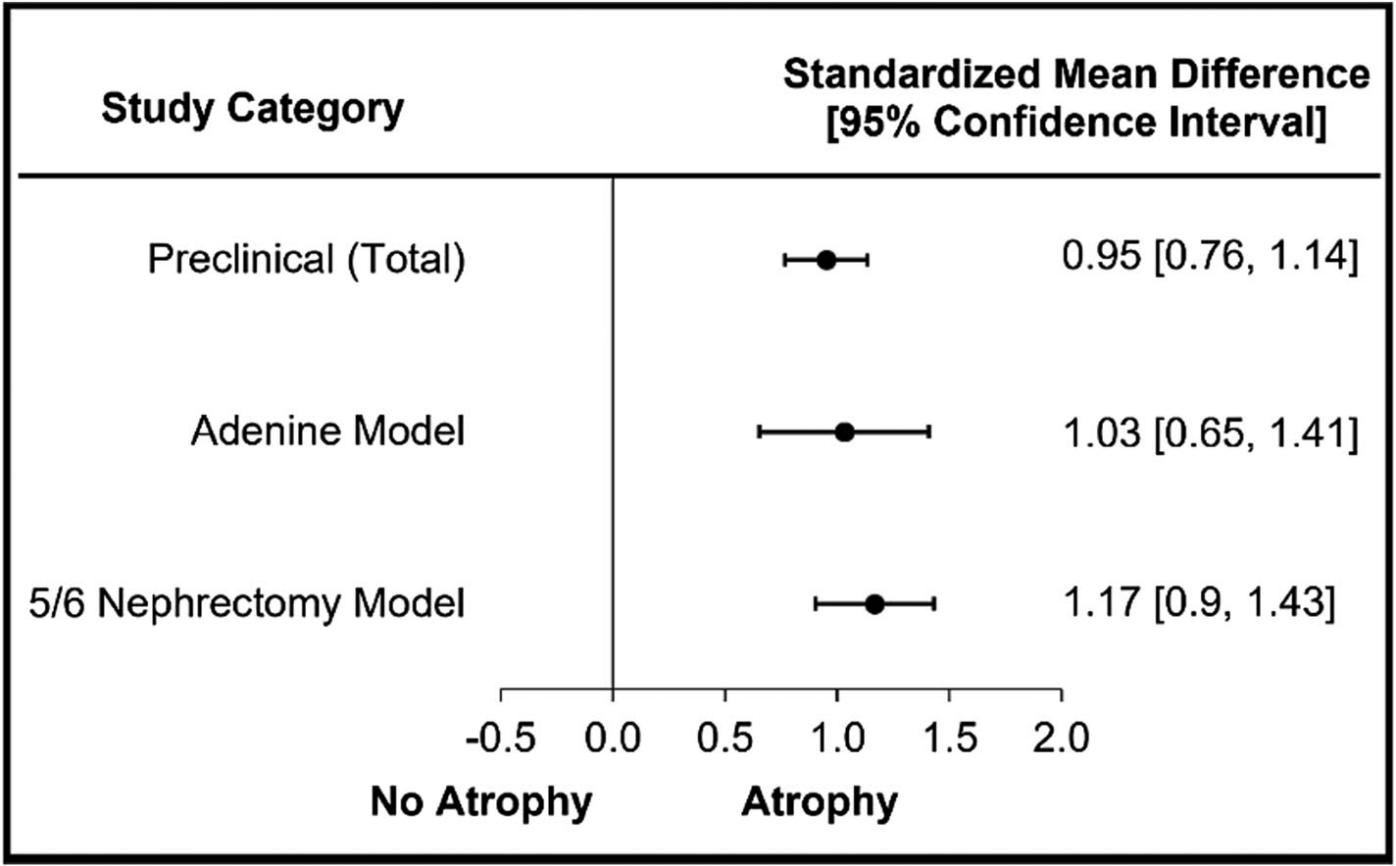
Muscle atrophy is significant in preclinical CKD.

Twenty‐nine out of 37 (78%) preclinical studies reported muscle atrophy, regardless of CKD model or species. A large significant difference was present for the total preclinical group (SMD = 0.95, 95% CI = 0.76–1.14, *I* ^2^ = 46.79; Figure 4). Sub‐analyses based upon CKD induction method (i.e., animal model) were performed for adenine diet and 5/6 nephrectomy only; the Cy/+ model did not meet the three‐study threshold. A large significant difference was present for both adenine‐induced CKD (SMD = 1.03, 95% CI = 0.65–1.41, *I* ^2^ = 12.30; Figure 4 and S15) and 5/6 nephrectomy‐induced CKD (SMD = 1.17, 95% CI = 0.90–1.43, *I* ^2^ = 57.70; Figure 4 and S16).

### Assessment methods of muscle atrophy: Preclinical studies

**Figure 5:**
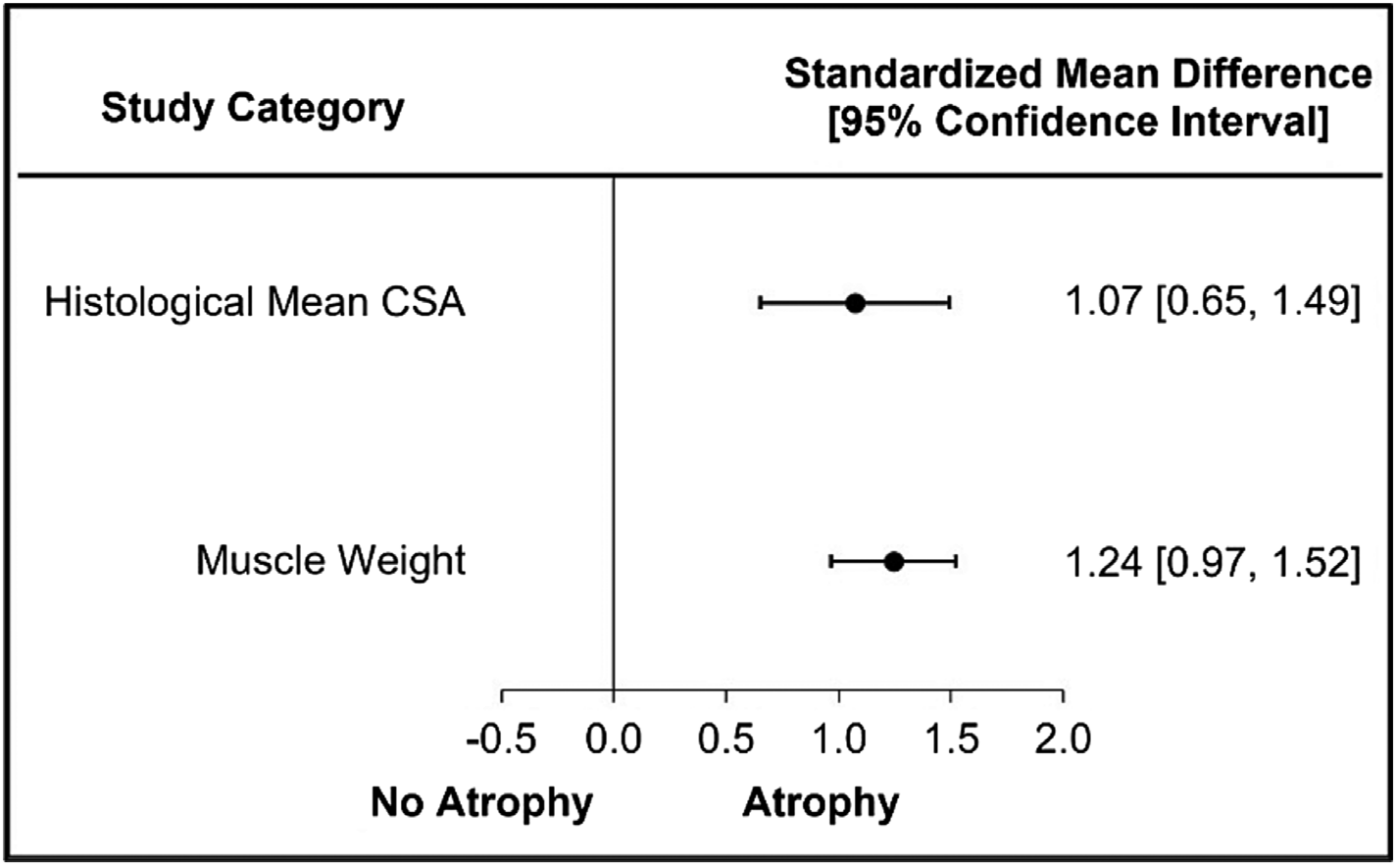
Muscle atrophy is significant in preclinical measurements of skeletal muscle atrophy.

Muscle atrophy determined by histological mean CSA demonstrated muscle atrophy in 18 of 22 studies (82%) with a large significant difference (SMD = 1.07, 95% CI = 0.65–1.49, *I* ^2^ = 50.67; Figure 5 and Figure S17). Muscle atrophy determined by muscle weight demonstrated muscle atrophy in 23 of 26 studies (88%) with a large significant difference (SMD = 1.24, 95% CI = 0.97–1.52, *I* ^2^ = 47.99; Figure 5 and Figure S18).

### Assessment of atrophy in muscle fibre types

**Figure 6:**
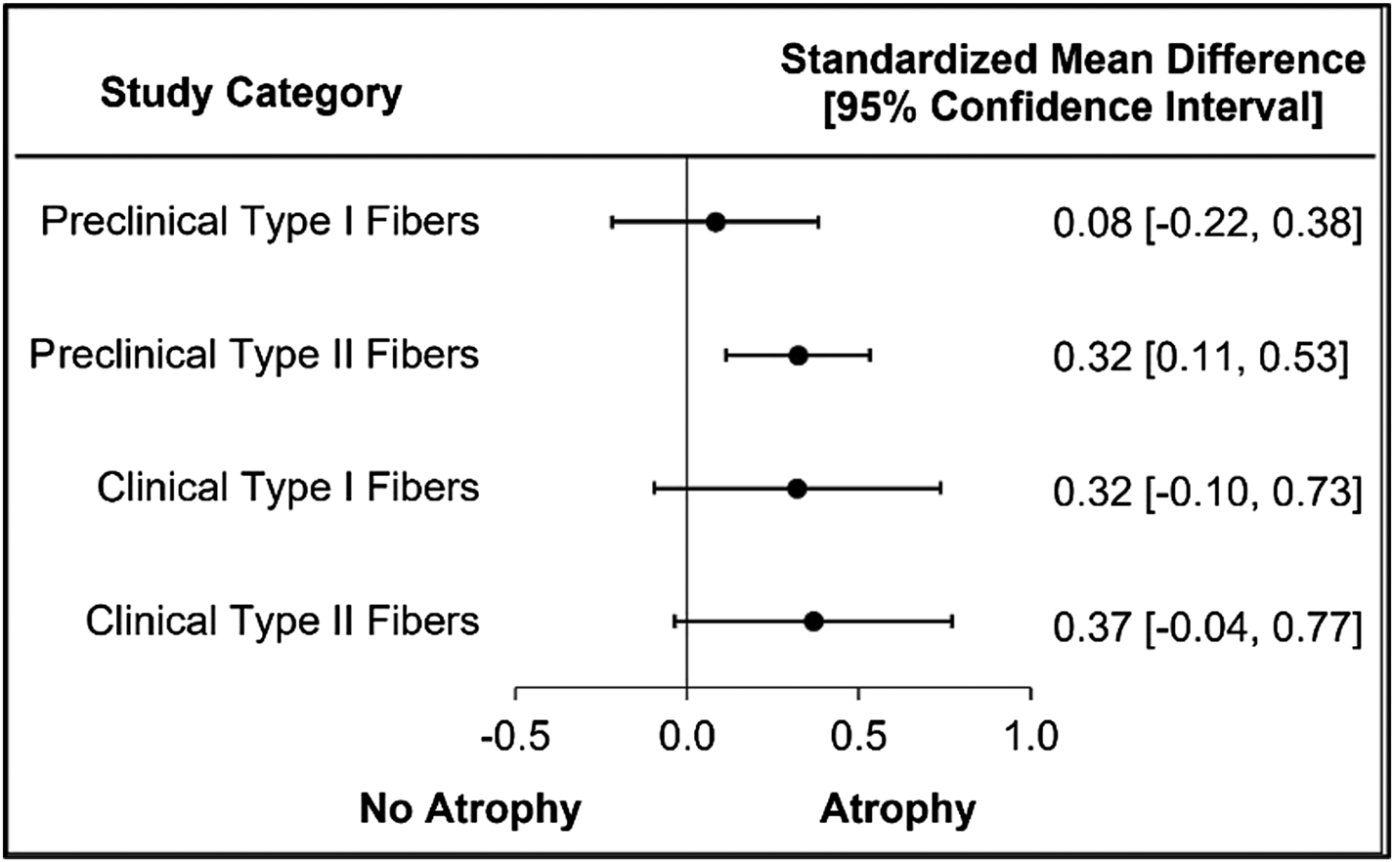
Muscle atrophy is fibre type specific in preclinical, but not clinical CKD.

A small number of clinical and preclinical studies assessed atrophy based upon CSA per muscle fibre type (i.e., type II/fast muscle fibre, type I/slow muscle fibre). Clinically, significant muscle atrophy was reported in three of seven studies (43%) for type II CSA and two of seven studies (29%) for type I CSA. Preclinically, significant muscle atrophy was reported in two of three studies (67%) for both types I and II muscle fibres. Type I muscle fibres did not demonstrate significant mean differences for atrophy in preclinical (SMD = 0.08, 95% CI = −0.22 to 0.38, *I* ^2^ = 0.00; Figure 6 and Figure S19) nor clinical (SMD = 0.32, 95% CI = −0.10 to 0.73, *I* ^2^ = 0.00; Figure 6 and Figure S20) studies. Type II fibres did not demonstrate significant mean differences for atrophy in clinical studies (SMD = 0.37, 95% CI = −0.04 to 0.77, *I* ^2^ = 23.42; Figure 6 and Figure S21), but a small significant difference was demonstrated in preclinical studies (SMD = 0.32, 95% CI = 0.11–0.53, *I* ^2^ = 0.79; Figure 6 and Figure S22).

### Assessment of muscle atrophy by sex

**Figure 7:**
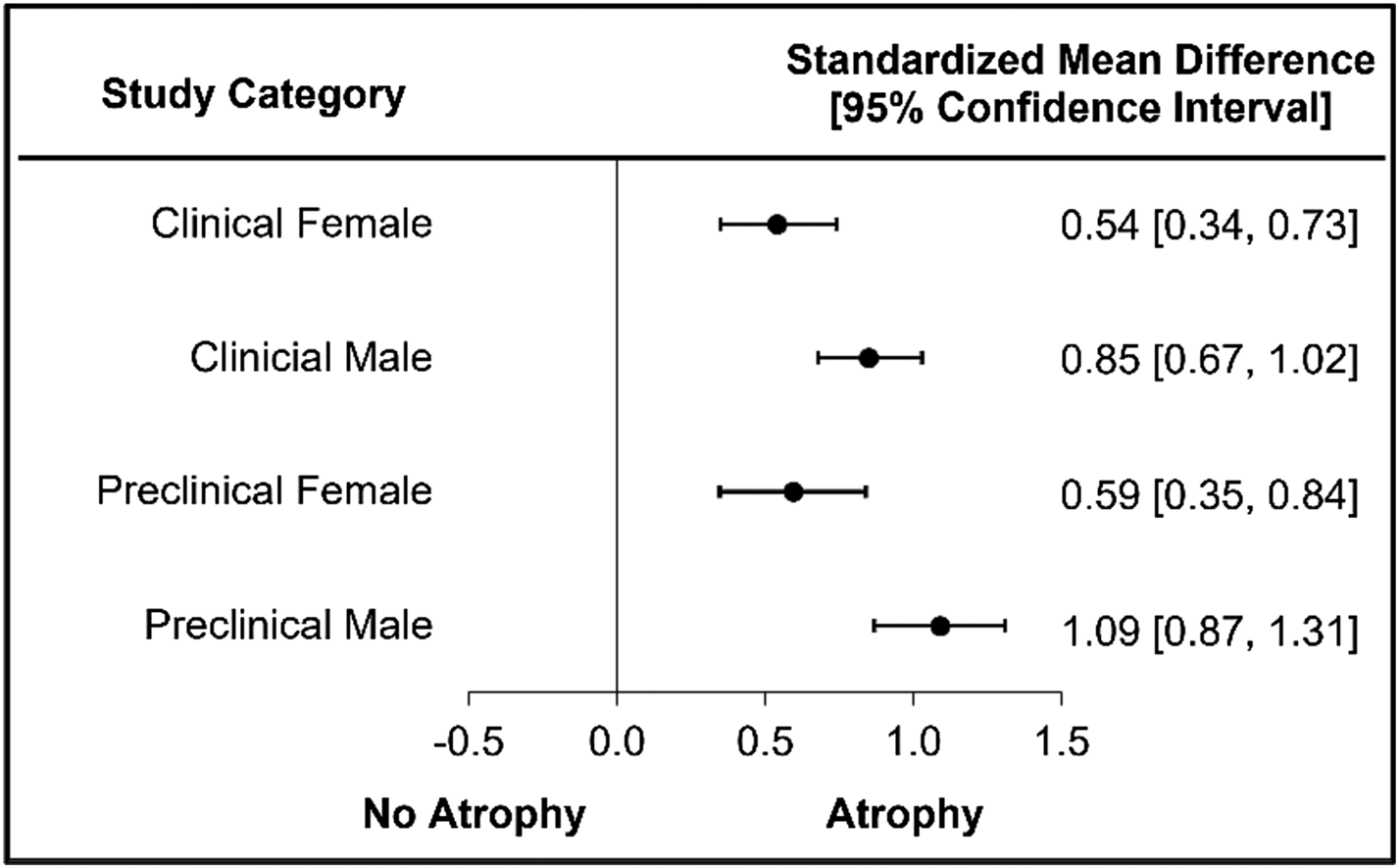
Muscle atrophy is sex specific in preclinical and clinical CKD.

Clinical studies reported significant muscle atrophy for female participants in five of eight (63%) studies with a medium significant difference (SMD = 0.54, 95% CI = 0.34–0.73, *I* ^2^ = 28.92; Figure 7 and Figure S23). Male clinical studies reported significant atrophy in nine of 14 (64%) studies with a large significant difference (SMD = 0.85, 95% CI = 0.67–1.02, *I* ^2^ = 78.75; Figure 7 and Figure S24). Preclinical female studies reported significant muscle atrophy in three of five (60%) studies with a medium significant difference (SMD = 0.59, 95% CI = 0.35–0.84, *I* ^2^ = 0.00 Figure 7 and Figure S25). Preclinical male studies reported significant atrophy with a large significant difference (SMD = 1.09, 95% CI = 0.87–1.31, *I* ^2^ = 50.51; Figure 7 and Figure S26) studies. Sex differences were consistent across clinical/preclinical studies with medium differences for females and large differences for males.

### Quality of the evidence

The overall quality of the clinical studies per the NOS assessment tool, was poor for 6 studies, fair for 56, and good for 7 studies (Figure S27, Tables S3 and S4). The overall quality for the transplant studies was poor for one study, fair for five studies, and good for one study (Figure S28). Preclinical studies were not assessed based on overall quality, but rather individual components of the study (detailed in the methods). The categories from the Syrcle's assessment tool that had >70% of the studies score low risk included baseline characteristics, random housing, detection blinding and reporting bias; while all other categories consisted primarily of unclear risk (Figure S29; Table S5).

## Discussion

Muscle atrophy is a primary component of sarcopenia that leads to impaired function and mobility. Identifying the extent of muscle atrophy is of clinical value to guide decision‐making for the management of musculoskeletal health. In CKD, skeletal muscle atrophy has been inconsistently reported in the literature. We utilized systematic and meta‐analytic techniques to identify the magnitude of muscle atrophy in clinical and preclinical studies. Among the 106 clinical and preclinical CKD studies that were included in this meta‐analysis (excluding transplant), a total of 63 studies reported significant skeletal muscle atrophy with a *P*‐value <0.05 in at least one measure of muscle size. The combined clinical and preclinical SMD for skeletal muscle atrophy in CKD was a medium significant difference of 0.58 (95% 95% CI = 0.52–0.64). These findings indicate that skeletal muscle atrophy is a common feature of both clinical and preclinical presentation of CKD.

The presence of skeletal muscle atrophy in CKD was accounted for with considerations for disease stage, presence of diabetes, transplant and dialysis. Patients with CKD stages III–IV and ESKD (PD) demonstrated a small significant difference, while those with ESKD (HD and total) demonstrated a medium significant difference in muscle atrophy. The effect sizes per the CKD stages differed, but the magnitude of the effect sizes was not overly impressive. The analyses for ESKD patients who were not on dialysis, resulted in insignificant muscle atrophy. This observation may appear to be counterintuitive, but the lack of atrophy observed in the population may result from confounding factors such as volume overload. DEXA and BIA can overestimate muscle mass in individuals with extracellular fluid accumulation, which is more pronounced in ESKD patients compared with CKD stages III–IV.^14^, ^15^ Spectroscopy has higher predictive power than BIA methods as the frequency ranges increase reliability when hydration levels are altered. To improve predictive power of muscle mass in overhydrated patients, a specific algorithm must be applied.^16^ Patients with ESKD may experience a false‐positive reversal of skeletal muscle atrophy due to volume overload.^17^ Future studies are needed to provide further insight into how volume overload impacts the assessment of muscle atrophy in the latter stages of CKD.

Three sub‐analyses performed for CKD‐diabetes properly controlled, transplant and dialysis modality demonstrated unexpected findings. The diabetes sub‐analyses demonstrated a medium significant difference (SMD = 0.56, 95% CI = 0.44–0.67) in atrophy for studies that controlled or excluded for diabetes, while studies that did not control for diabetes demonstrated a lower SMD (SMD = 0.44, 95% CI = 0.36–0.52). It is important to highlight that diabetes studies were categorized as either allowing subjects with diabetes or specifically excluding those with diabetes. Proper within‐study comparisons for participants with diabetes compared with those without diabetes were not performed and prevent definitive conclusions to be made on the impact of diabetes and muscle atrophy. The classification of properly or not properly controlled were used due to this limitation in study design. We suggest future studies better control for the inclusion/exclusion of diabetes to fully appreciate the impact it may have upon muscle size. The authors originally expected a large SMD given the inclusion of two comorbidities, but surprisingly found a small to medium significant differences. Further studies should be performed to determine the impact that diabetes may have upon muscle atrophy in those with CKD. In dialysis‐specific studies, patients receiving HD demonstrated a medium significant difference in muscle atrophy while PD patients demonstrated a small significant difference. A recent systematic review and meta‐analysis found that the prevalence of sarcopenia was higher in HD populations (31%, 95% CI: 24.7–37.3) than PD populations (23.4%, 95% CI: 11.9–34.9), though the difference was not statistically significant.^18^ In support of our findings, patients receiving HD often report a lower quality of life (QOL) compared with PD, which could be impacted by greater muscle atrophy.^19^ Finally, in our meta‐analysis participants who received a renal transplant demonstrated a small SMD, which is aligned with previous studies showing a prevalence of sarcopenia ranging from 7%^20^ to 33%^21^ in this population, depending on the diagnostic criteria used. These findings suggest that kidney transplantation does not reverse CKD‐associated sarcopenia. The presence of muscle atrophy in kidney transplant recipients has been variably attributed to impaired renal function, the use of corticosteroid therapy and diabetes^22^, ^23^, ^24^ [S33]. Clinically these findings could help guide future research studies as well as clinical management of musculoskeletal health in CKD.

Clinical studies that assessed cross‐sectional area were drastically different based upon assessment method, with a large SMDs for MRI and a small SMD for histological assessment. Despite differences in the SMD, the bodily site locations for MRI and histology similarly included the gastrocnemius, soleus, tibialis anterior and quadriceps, excluding this as an underlying contributor to the observed differences. Both assessment methods included similarly staged participants who were ESKD primarily on HD. Although there could be additional explanations, we postulate that the observed differences may be attributed to assessment of the whole muscle in MRI as opposed to a small fraction of the muscle with histology. It is important to highlight that these sub‐analyses are not directly comparing assessment method, but rather providing mean differences for each method. Six studies included multiple assessment methods (not limited to CSA) with demonstrated consistency among SMDs or significance in three of the studies. The SMDs for anthropometry were different from BIA in three studies, but there a was lack of direction agreement among the comparisons, preventing definitive conclusions from being made between these assessment methods^25^ [S22, S35]. Further investigation is needed specifically comparing muscle atrophy across different assessment methods while controlling for factors of disease stage, age, BMI.

Preclinical models of CKD demonstrated a large significant mean difference indicating muscle atrophy. Studies involving preclinical CKD models utilized multiple species including rats, mice and rabbits.^26^ CKD is commonly induced in these models via diet (such as adenine‐diet), surgical induction (5/6 nephrectomy) or missense gene mutations (Cy/+ progressive).^13^, ^27^, ^28^ The Cy/+ progressive method for CKD was only utilized by one study, therefore it was not included as a sub‐analysis.^28^ In a unique study that included both the 5/6 nephrectomy and adenine models, similar changes were demonstrated in disease severity and muscle atrophy^13^; a finding in line with our current analysis. Animal studies are conducted under the guise that a specific disease model will closely mimic human pathology, and ultimately guide disease management with reduced patient burden.^29^ Unfortunately, there are few animal models of CKD that reproduce all human pathophysiological ailments.^26^ Due to the various methods of measuring muscle size/mass, we cannot make direct comparisons between clinical and preclinical models but will draw attention and appreciation to the SMD differences. Clinical studies reported muscle atrophy in 49% of studies with a small SMD, while preclinical studies reported muscle atrophy in 78% of studies with a large SMD. The incongruency between clinical and preclinical models supports the need to identify which animal model best represents the clinical presentation of muscle in CKD. The incongruency could potentially be explained by metabolic acidosis, which has been shown to be quite impactful to protein degradation and muscle atrophy.^30^ Unfortunately, many preclinical and clinical studies do not assess bicarbonate and blood gases consistently to fully appreciate the impact of metabolic acidosis in clinical and preclinical studies. Further, there is often a desire in studies to show that atrophy is occurring, but with many clinical studies (51%) not showing atrophy, then it is unclear what preclinical model is most representative. It maybe that a preclinical study without atrophy present is viewed as a ‘negative study’ but may be the most translational and representative of clinical changes seen in CKD. Further investigations are needed into how the presence or absence of muscle atrophy in CKD impacts muscle function and mobility.

In addition to the differences found between assessment methods, histological cross‐sectional area demonstrated greater atrophy in a fibre‐specific manner for preclinical studies only. Preclinical type II fibres displayed significant atrophy with a small SMD, with no differences found in type II clinical or type I preclinical and clinical; note the confidence interval was nearly significant (−0.04) for clinical type II CSA. The overall variance of the clinical histological measurements was much larger than that of the preclinical fibre types. This variance could be due to the method of obtaining muscle biopsies in humans versus obtaining muscle samples from animals. Human muscle biopsies are a representative sample of the muscle that may lose its structure during preparation for histology.^31^ In contrast, rodent skeletal muscle is prepared with the whole muscle intact, decreasing the potential for structural changes or variation in fibre size. Regardless, some clinical studies reported type I muscle atrophy (29%) and type II muscle atrophy (49%) indicating that atrophy does occur. Overall, our findings suggest that a standardized muscle atrophy measurement needs to be utilized in research and clinical settings to assess the presence of atrophy accurately and consistently across clinical and preclinical models.

Analysis of biological sex (males, females) demonstrated significant differences in both clinical and preclinical chronic kidney disease. Females demonstrated a medium effect size, while males demonstrated a large effect size in both preclinical and clinical CKD. This suggests that skeletal muscle responds differently in males and females to the pathophysiological process that occurs in chronic kidney disease. The differences in muscle atrophy between sex, may be related to differing muscle compositions, anabolic (i.e. satellite cell activation) and catabolic (i.e. ubiquitin‐proteasome activity, autophagy activity) factors, hormonal interactions, and mitochondrial content and function.^32^ This is an important distinction as few clinical studies parcelled out female and male muscle atrophy data. Only five preclinical studies investigated muscle atrophy in female animals, compared with the 39 studies that reported male data. More studies should investigate the pathophysiology of females with CKD to improve our understanding of the disease mechanisms and to optimize therapeutic interventions for this subset of the population.

### Strengths and limitations

This is the first meta‐analysis to analyse skeletal muscle atrophy in both clinical and preclinical models of CKD. Inclusion of both preclinical and clinical studies allowed a comprehensive understanding of the current literature regarding muscle size changes in CKD. The authors acknowledge that muscle mass was measured with a multitude of methods that have intrinsic differences.^33^ For example, myofibre CSA gives insight to fibre size of each muscle fibre, while DEXA determines an individual's total or appendicular lean mass. Differences between measurement methods may have affected the identification of atrophy in the individual studies.^33^ In addition to shear intrinsic difference between measurement methods, changes in fluid volume (i.e., dialysis timing) must be kept in consideration because BIA was used to measure lean mass.^34^ Another limitation of the literature was the inconsistent CKD clinical staging that required grouping by stages III–IV and ESKD, limiting the authors ability to assess for atrophy changes throughout the disease progression. The degree of muscle wasting in different stages of CKD and different preclinical models of CK may also be impacted by the different assessment methods included. To be comprehensive all methods were included in this analysis. As previously discussed, studies with multiple assessments found varying degrees of consistency regarding magnitude and significance of atrophy. Given the variable levels of resources and research environment it was deemed most appropriate to include all methods and appreciate that differences may be confounded by assessments methods. Differences in muscle atrophy may have also been affected by physical activity/inactivity. The individual studies did not control for physical activity, which could have resulted in varying levels of atrophy due to training effects. A potential bias would be the lack of reporting in studies without significant atrophy. Given the inconsistent demonstration of significant atrophy in clinical (49%) and preclinical (78%) studies, investigators should be encouraged to report all muscle size outcomes despite findings of significance. A potential strength and/or limitation was the a priori decision to exclude prevalence studies. A recent prevalence study found sarcopenia to be very common in those with kidney transplant recipients.^35^ They also found similar prevalence between males and females following kidney transplant. Our specific analysis did not explore sex differences in the kidney transplant population, but we did find a sex difference in preclinical and clinical studies regardless of stage or modality. It maybe that males and females have similar prevalence for atrophy, but males are impacted to a greater extent than females. The review process was completed by multiple reviewers, which may have increased the likelihood of bias or error with study inclusion decisions or transposition of data. Despite these limitations, our findings point towards significant muscle atrophy in both preclinical and clinical CKD.

## Conclusions

In summary, clinical and preclinical models of CKD demonstrated small to large SMDs across multiple analyses including severity of CKD, dialysis modality, biological sex, and assessment method. Muscle atrophy was present in CKD, but we were unable to determine if there is a progressive loss with disease severity or if the loss occurs at the onset of dialysis. The preferential loss of fast fibre size was only found in preclinical studies with a small SMD but may have clinical implications given the role of these fibres in strength and power‐based movements in activities of daily living and recovery from perturbations [S39]. Finally, it appears that the preclinical models included in this analysis are appropriate for investigating the translational impact of sarcopenia in CKD. Future work would benefit from the use of standardized measurement methods and consistent clinical staging to improve our understanding of atrophy changes in CKD progression, and analysis of biological sex differences.

## Funding

KGA is supported by NIH NIDDK K08 DK110429 and R03 DK125665. ADT is supported by Foundation for Physical Therapy Research Promotion of Doctoral Studies (PODS) I.

## Conflict of interest

The authors declare no conflict of interest.

## Supporting information


**Data S1.** Supporting Information.

**Data S2.** Supporting Information.

**Data S3.** Supporting Information.

**Data S4.** Supporting Information.

## Data Availability

We conducted secondary analysis of publicly available data.
