# Supplementary material for: Skeletal muscle atrophy in clinical and preclinical models of chronic kidney disease: A systematic review and meta‐analysis
Source: J Cachexia Sarcopenia Muscle. 2023 Dec 7;15(1):21–35. doi: 10.1002/jcsm.13400 (PMC10834351; doi:10.1002/jcsm.13400)
Supplement: Supplementary file 1 — Data S1. Supporting Information. [file JCSM-15-21-s004.docx]

**Supplemental Material**

**Additional Search Strategies for Skeletal Muscle & CKD Systematic Review**

**Search originally ran on February 17, 2020. Update search ran on September 13, 2021.**

***Medline Ovid (1946-)***

**((Kidney Diseases/ or exp Renal Replacement Therapy/ or Renal Insufficiency/ or exp Renal Insufficiency, Chronic/ or dialysis.tw. or (hemodialysis or haemodialysis).tw. or (hemofiltration or haemofiltration).tw. or (hemodiafiltration or haemodiafiltration).tw. or (kidney disease* or renal disease* or kidney failure or renal failure).tw. or (predialysis or pre dialysis).tw.)**

**AND**

**(exp Muscle Fibers, Skeletal/ OR Muscle, Skeletal/ or exp Muscular Atrophy/ OR ((muscle OR muscular OR muscles) adj3 (skeletal OR voluntary OR atroph* OR wasting OR thickness OR mass)).tw OR (lean adj3 mass).tw OR sarcopeni*.tw))**

**not (letter OR editorial or news or comment).pt.**

**Filter: English**

**Original search 1946-2/17/2020: 3299 results**

**Update (2/17/2020 – 9/13/2021): 415 results**

***EMBASE (Elsevier)***

**('kidney disease'/de OR 'kidney failure'/de OR 'chronic kidney failure'/de OR 'mild renal impairment'/de OR 'moderate renal impairment'/de OR 'severe renal impairment'/de OR 'renal replacement therapy-dependent renal disease'/de OR 'renal replacement therapy'/de OR 'subclinical renal impairment'/de OR 'subclinical renal impairment'/de OR 'end stage renal disease'/de OR 'kidney transplantation'/de OR 'diabetic nephropathy'/de OR hemodialysis:ab,ti OR haemodialysis:ab,ti OR hemofiltration:ab,ti OR haemofiltration:ab,ti OR hemodiafiltration:ab,ti OR haemodiafiltration:ab,ti OR dialysis:ab,ti OR predialysis:ab,ti OR pre‐dialysis:ab,ti OR ((kidney OR renal) NEAR/1 (transplant* OR graft* OR allograft*)):ab,ti OR 'kidney disease*':ab,ti OR 'renal disease*':ab,ti OR 'kidney failure':ab,ti OR 'renal failure':ab,ti OR 'diabetic nephropath$':ab,ti OR 'diabetic kidney disease$':ab,ti)**

**AND**

**('skeletal muscle cell'/exp OR 'skeletal muscle'/de OR 'muscle atrophy'/exp OR ((muscle OR muscular OR muscles) NEAR/3 (skeletal OR voluntary OR atrop* OR wasting OR thickness OR mass)):ab,ti OR (lean NEAR/3 mass):ab,ti OR sacropeni:ab,ti)**

**Filters: English, article, article in press, review or short survey**

**Original search 1947-2/17/2020: 3598 results**

**Update (2/17/2020 – 9/13/2021): 646 results**

***Scopus***

**TITLE-ABS ((hemodialysis OR haemodialysis OR hemofiltration OR haemofiltration OR hemodiafiltration OR haemodiafiltration OR dialysis OR predialysis OR pre‐dialysis OR ((kidney OR renal) W/1 (transplant* OR graft* OR allograft*)) OR “kidney disease*” OR “renal disease*” OR “kidney failure” OR “renal failure” OR “diabetic nephropath*” OR “diabetic kidney disease*”))**

**AND**

**TITLE-ABS (((muscle OR muscular OR muscles) W/3 (skeletal OR voluntary OR atrop* OR wasting OR thickness OR mass))**

**OR (lean W/3 mass)**

**OR sacropeni*)**

**Include: English only**

**Exclude: Conference paper, editorial, erratum, letter, book, note, book chapter**

**Original search 1946-2/17/2020: 2722 results**

**Update (2/17/2020 – 9/13/2021): 423**

**Figure S1.** Identification of Studies. PRISMA Flow chart of screening process from the initial search to the final inclusion.


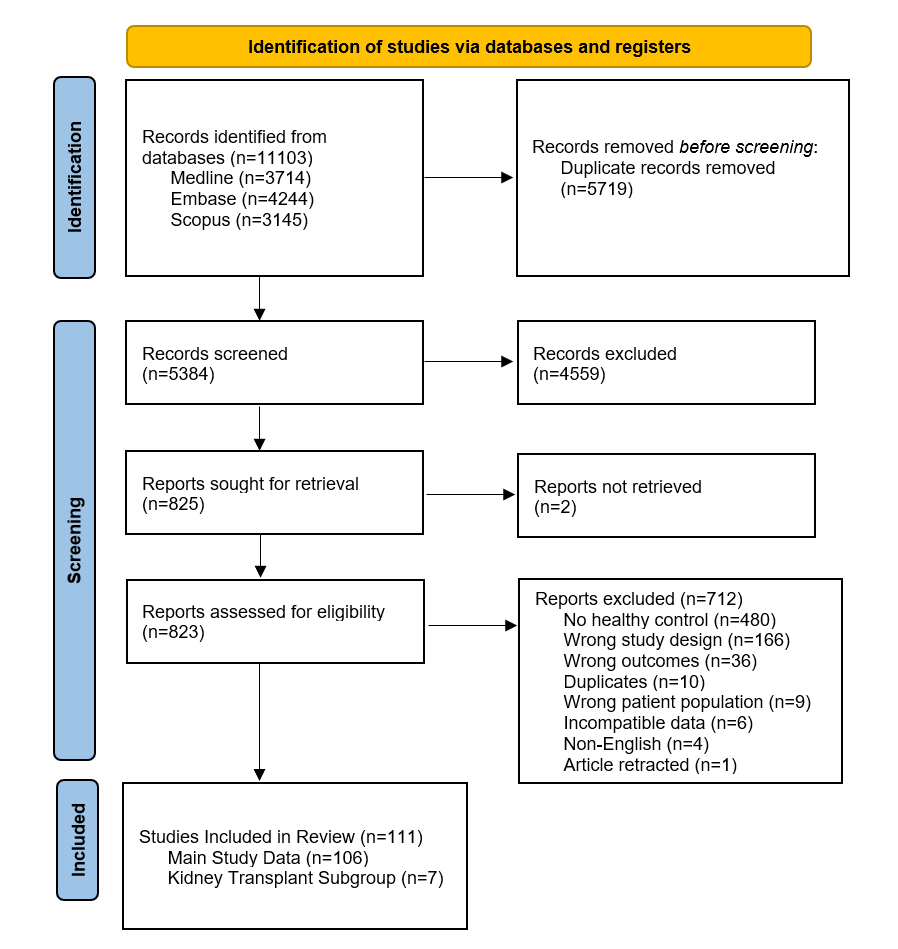


*From:*  Page MJ, McKenzie JE, Bossuyt PM, Boutron I, Hoffmann TC, Mulrow CD, et al. The PRISMA 2020 statement: an updated guideline for reporting systematic reviews. BMJ 2021;372:n71. doi: 10.1136/bmj.n71

**
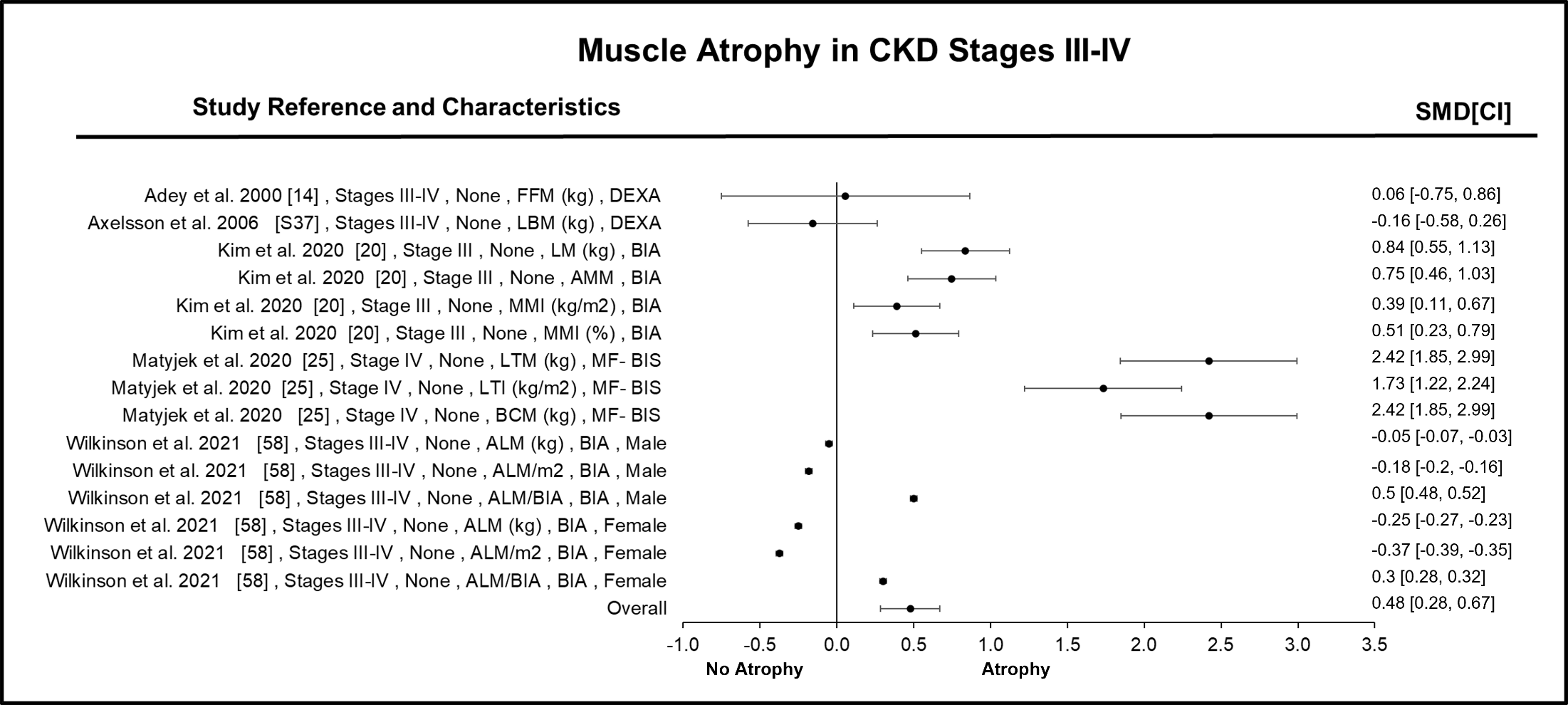
**

**Figure S2.** Muscle Atrophy in CKD Stages III-IV. SMD, standardized mean difference, CI, confidence interval. Random effects model used for analysis.

**
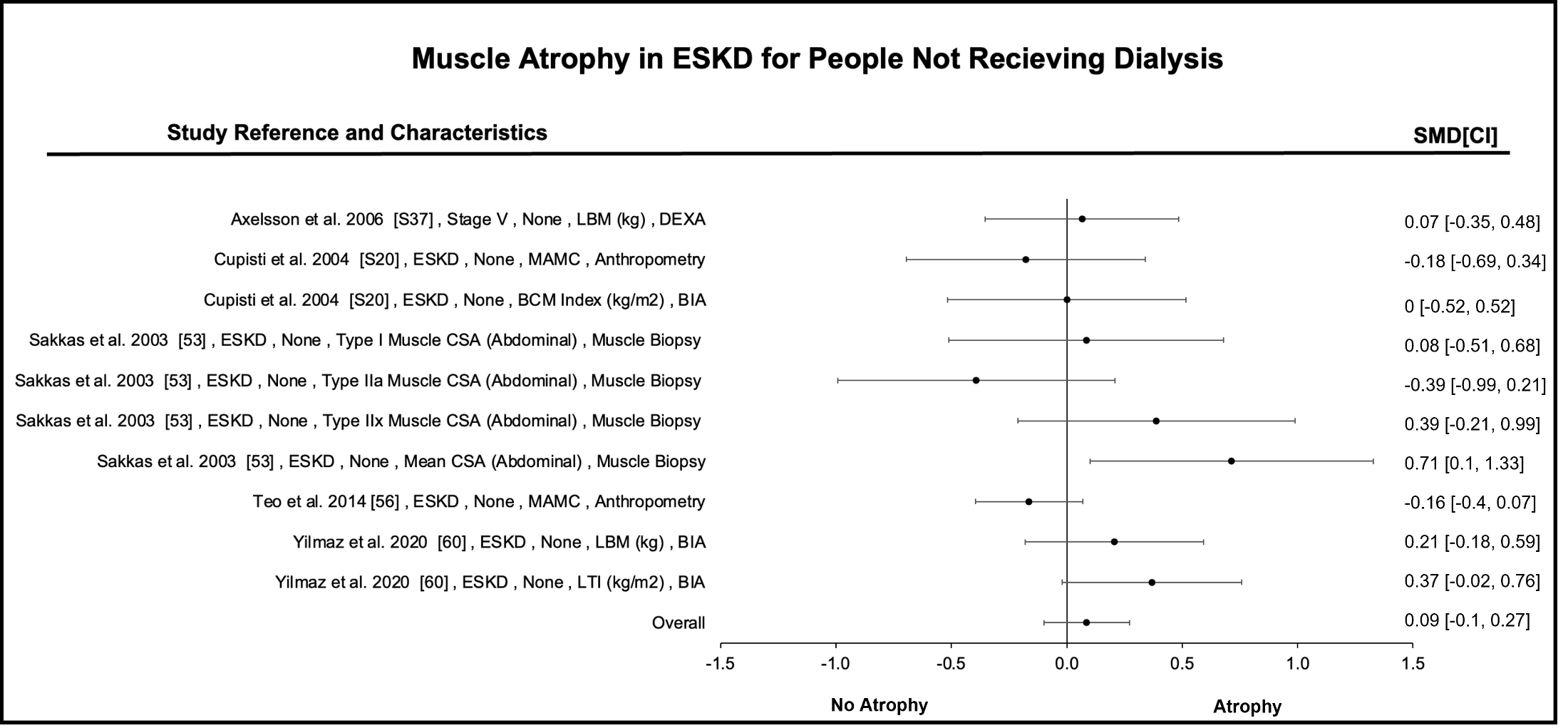
**


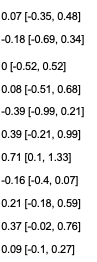
**Figure S3.** Muscle Atrophy in End-stage Kidney Disease (ESKD) Not Recieving Dialysis. SMD, standardized mean difference, CI, confidence interval. Random effects model used for analysis.

**
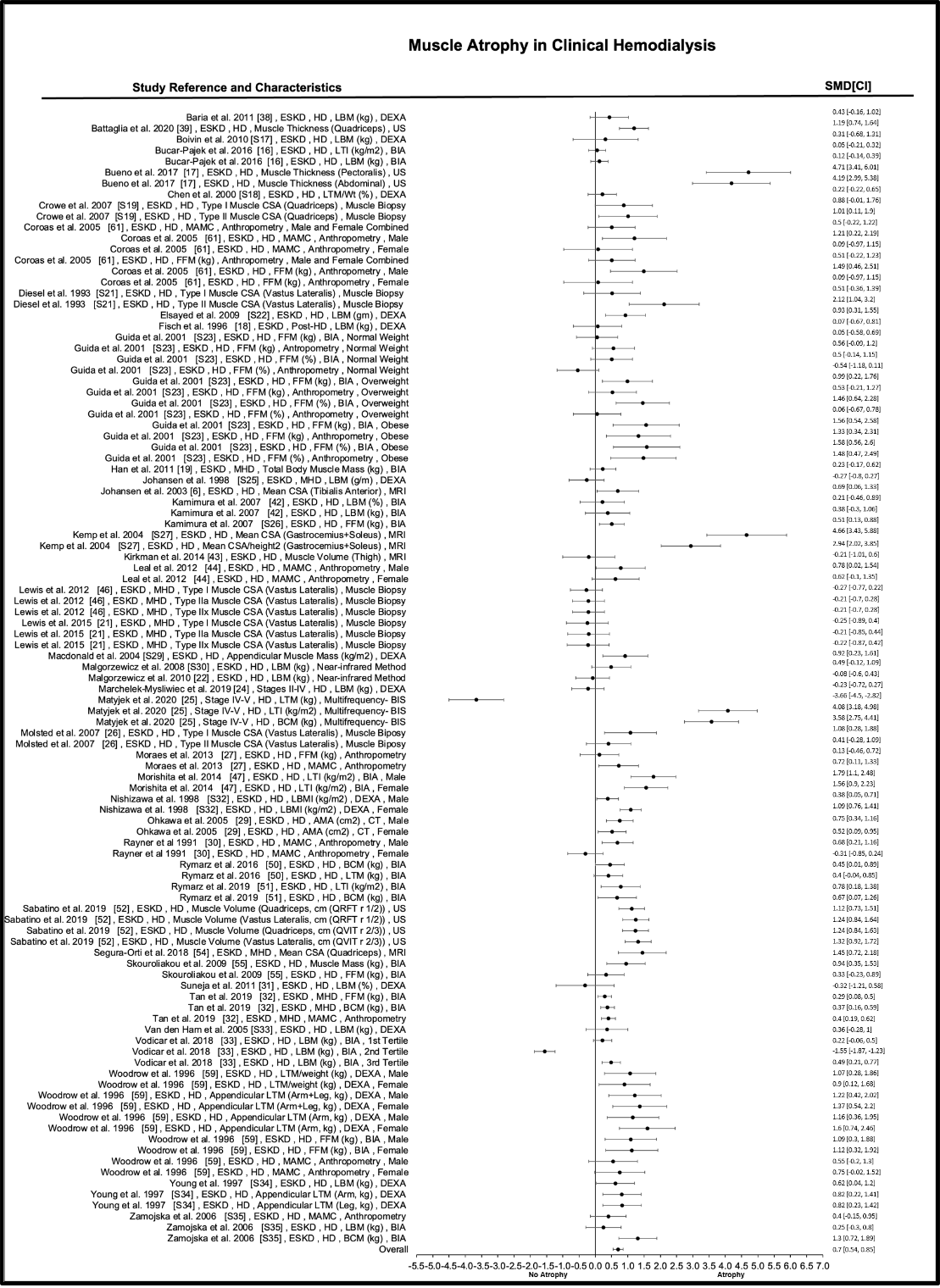
**

**Figure S4** Muscle Atrophy in Clinical Hemodialysis. SMD, standardized mean difference, CI, confidence interval. Random effects model used for analysis.


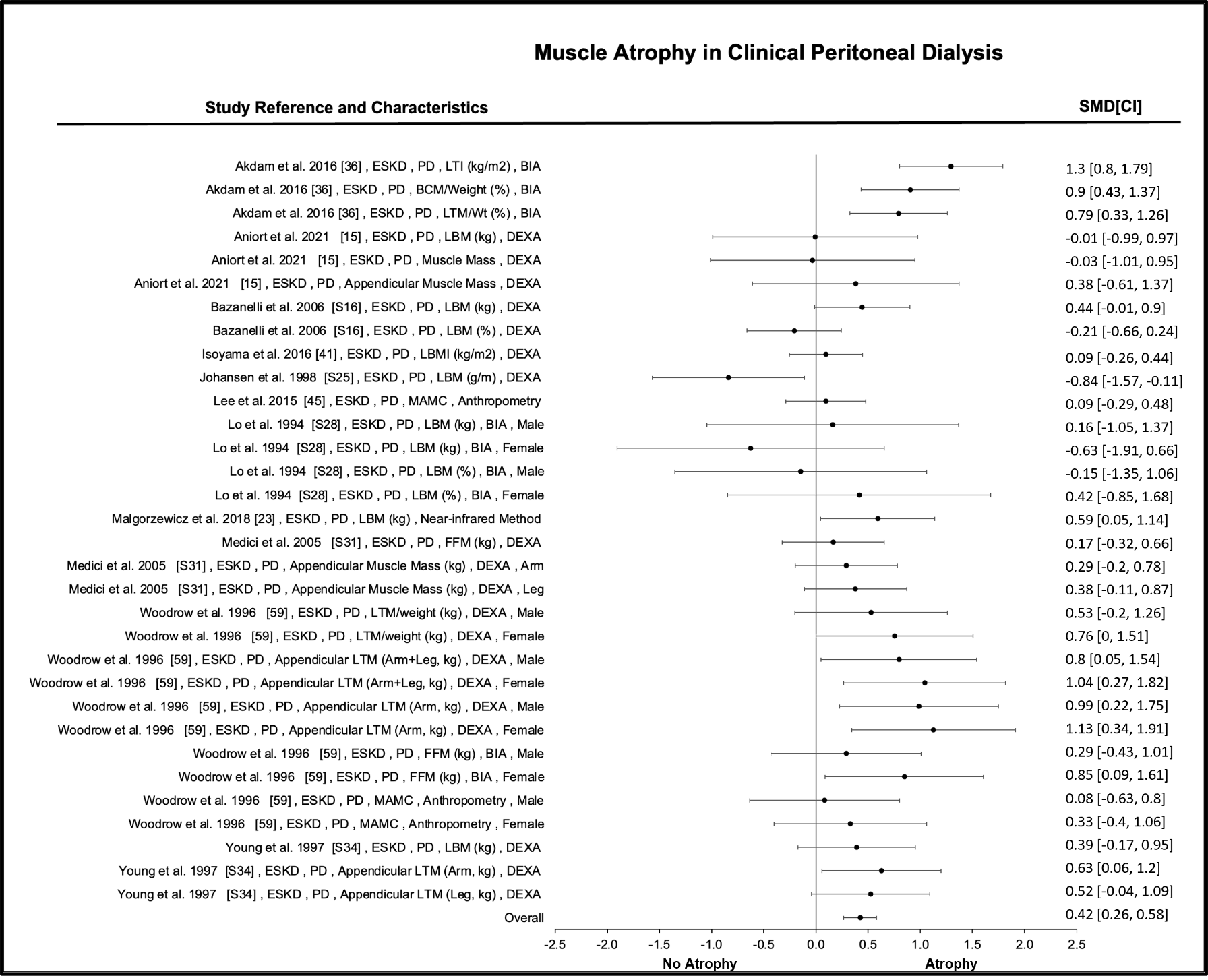


**Figure S5.** Muscle Atrophy in Clinical Peritoneal Dialysis. SMD, standardized mean difference, CI, confidence interval. Random effects model used for analysis

**
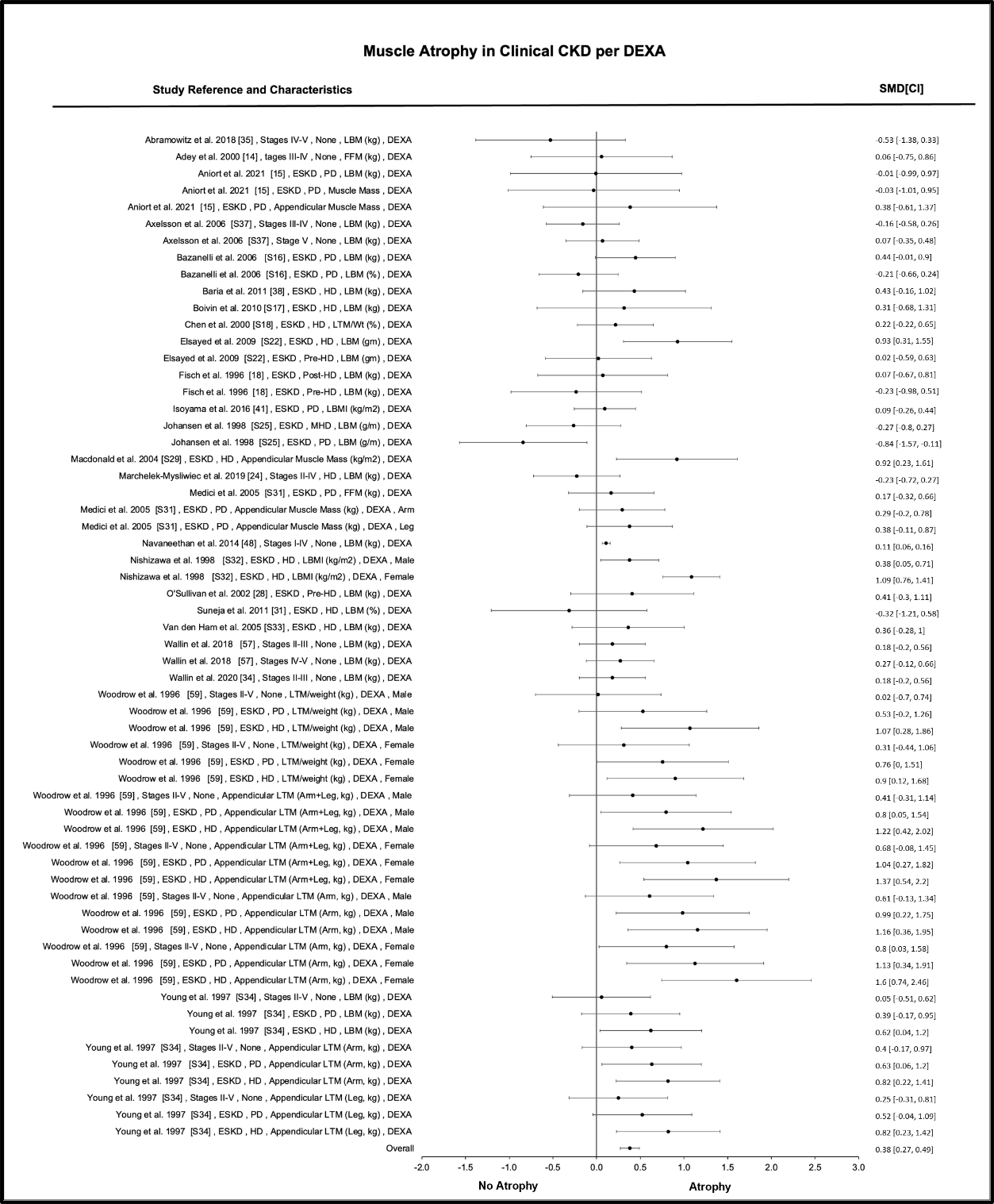
**

**Figure S6.** Muscle Atrophy in Clinical CKD per DEXA. SMD, standardized mean difference, CI, confidence interval. Random effects model used for analysis.

**
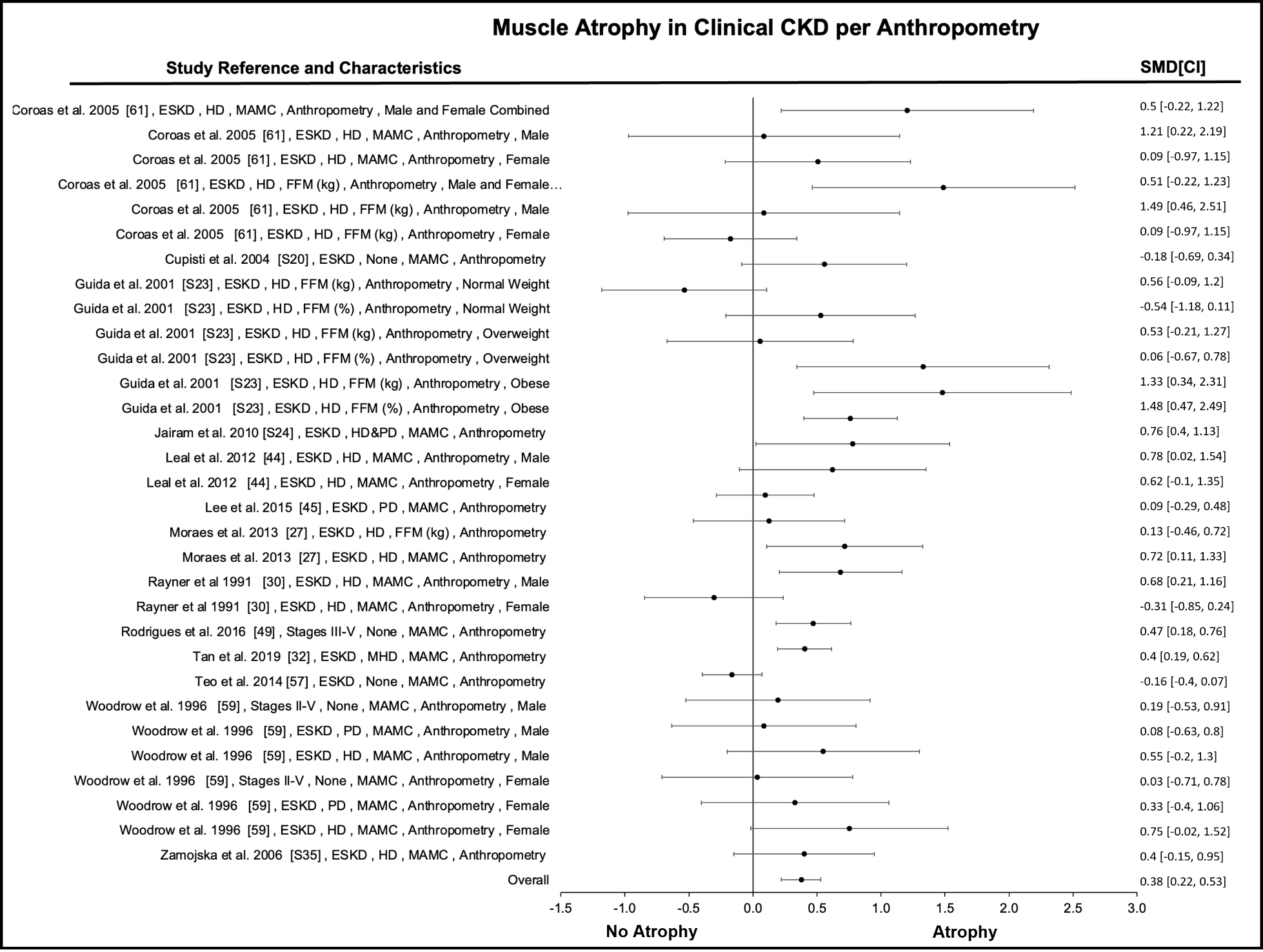
**

**Figure S7.** Muscle Atrophy in Clinical CKD per Anthropometry. SMD, standardized mean difference, CI, confidence interval. Random effects model used for analysis.


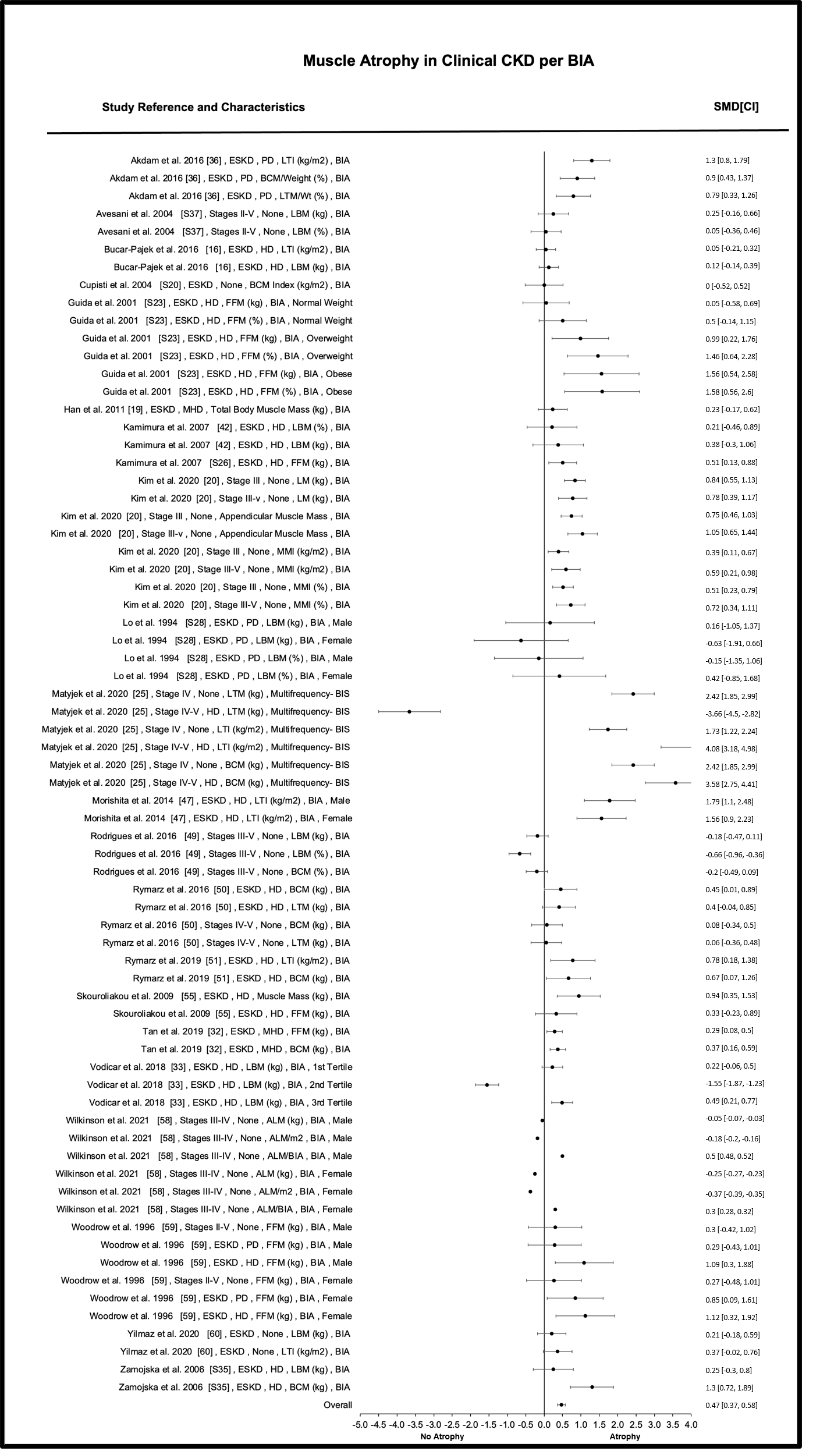


**Figure S8.** Muscle Atrophy in Clinical CKD per BIA. SMD, standardized mean difference, CI, confidence interval. Random effects model used for analysis.

**
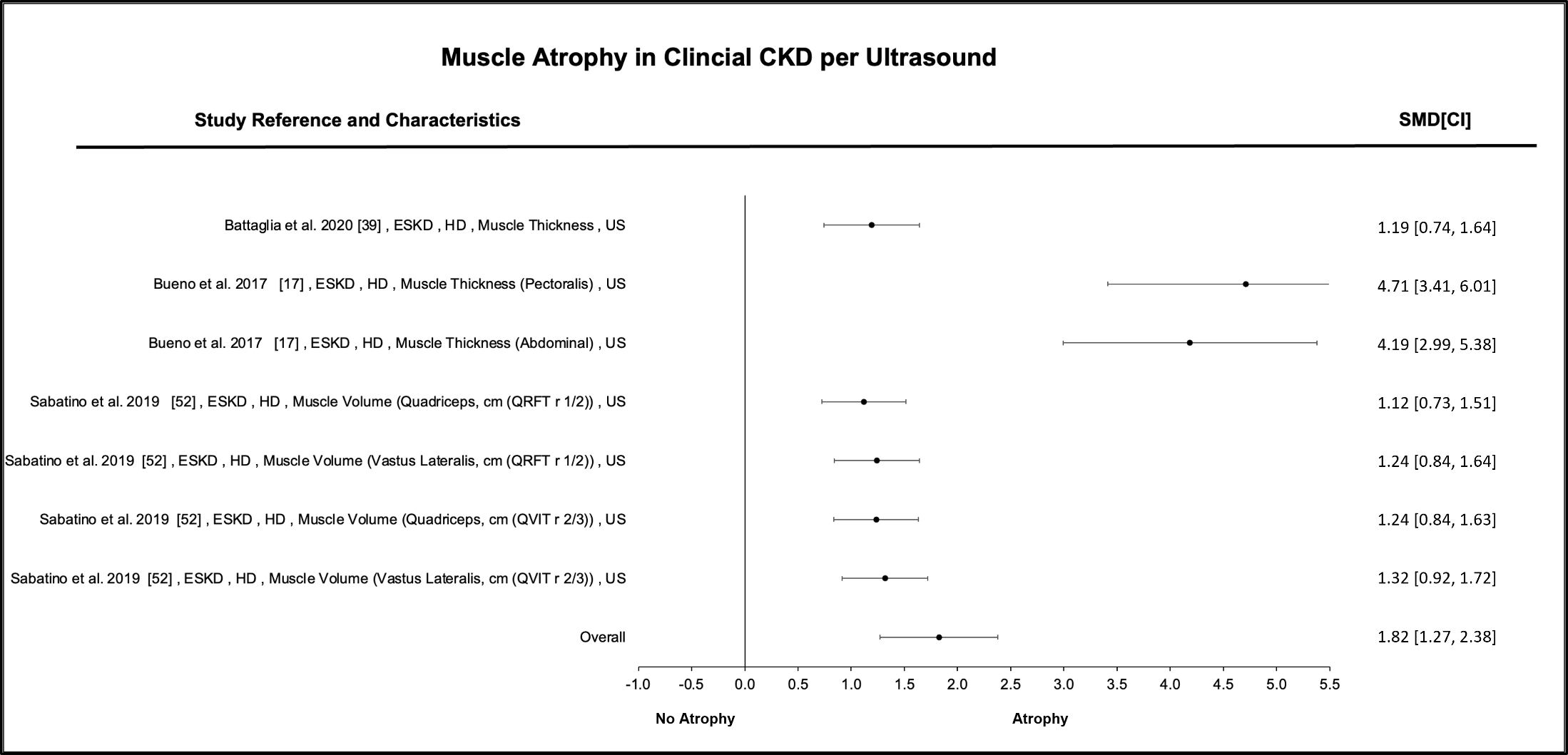
**

**Figure S9.** Muscle Atrophy in Clinical CKD per Ultrasound. SMD, standardized mean difference, CI, confidence interval. Random effects model used for analysis.


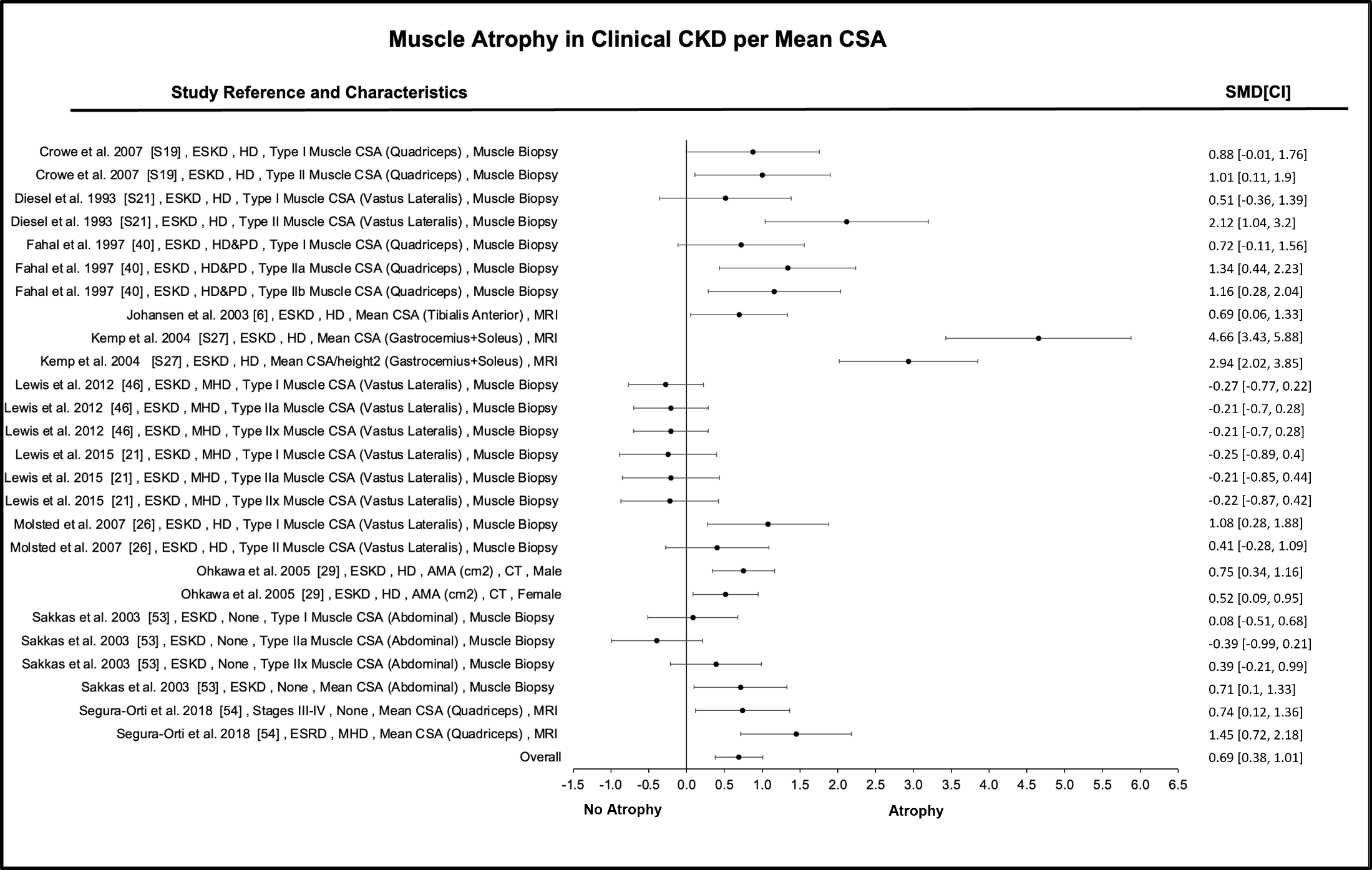


**Figure S10.** Muscle Atrophy in Clinical CKD per Overall Mean CSA, regardless of measurement method. SMD, standardized mean difference, CI, confidence interval. Random effects model used for analysis.


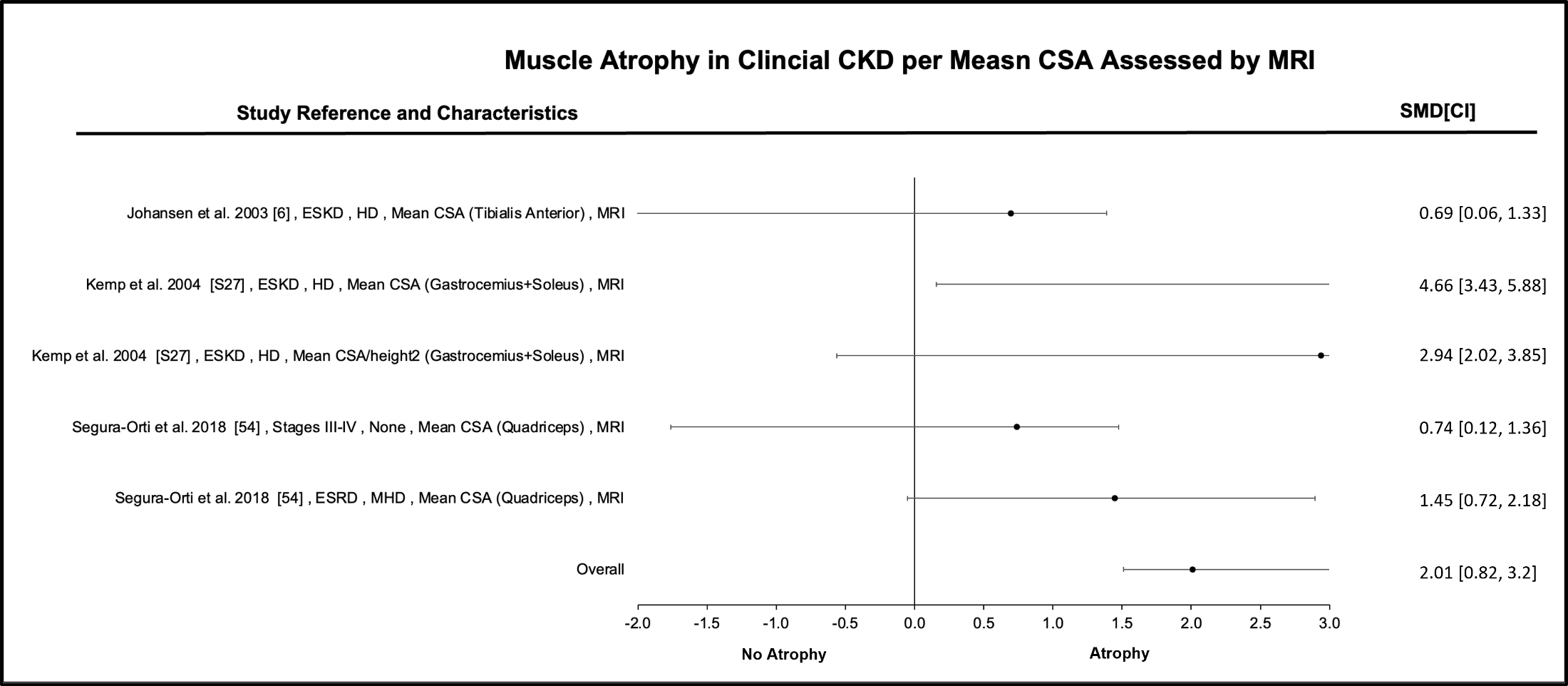


**Figure S11.** Muscle Atrophy in Clinical CKD per MRI Mean CSA. SMD, standardized mean difference, CI, confidence interval. Random effects model used for analysis.

**
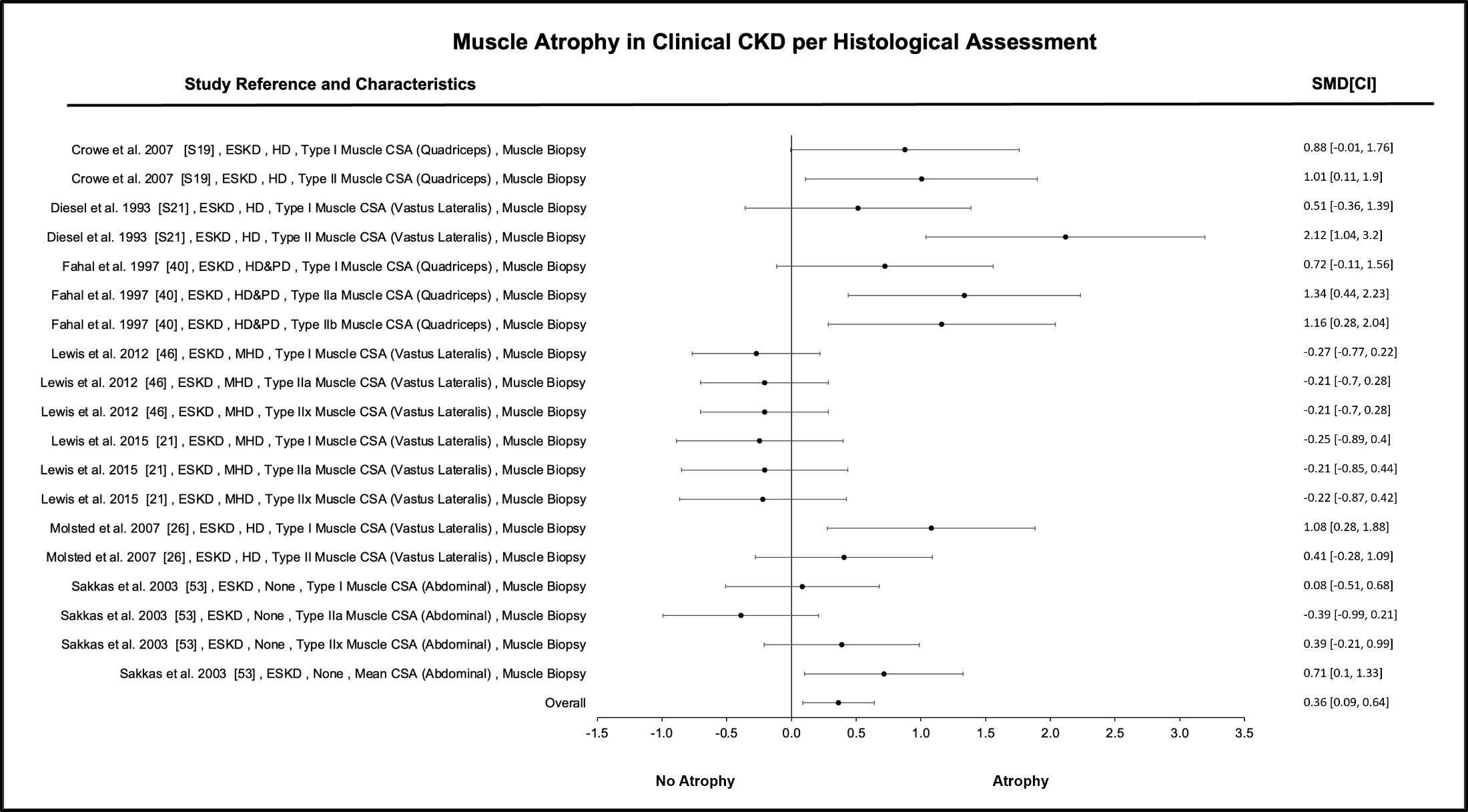
**

**Figure S12.** Muscle Atrophy in Clinical CKD per Histological CSA Assessment. SMD, standardized mean difference, CI, confidence interval. Random effects model used for analysis.

**
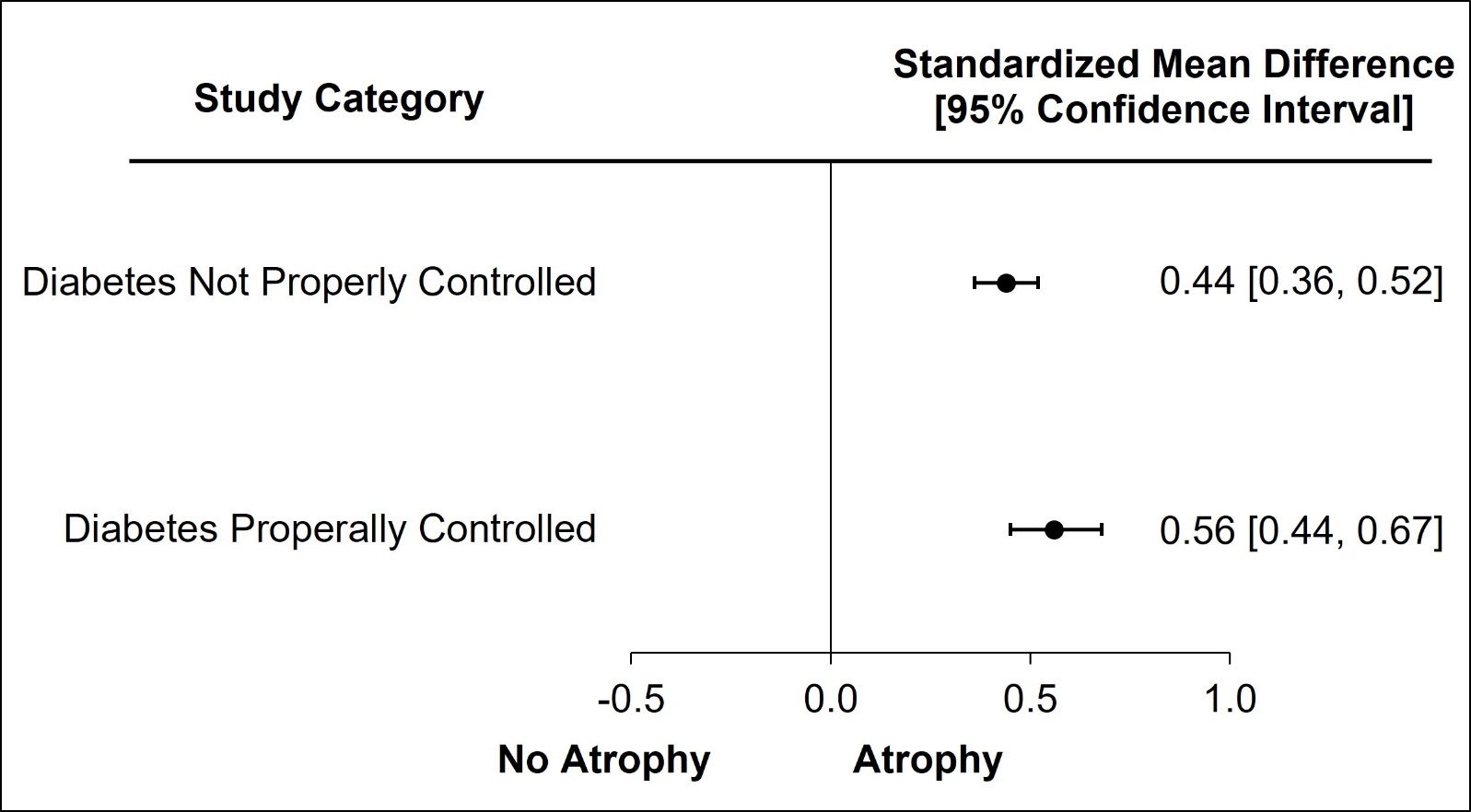
**

**Figure S13.** Muscle Atrophy in CKD and Diabetes. SMD, standardized mean difference, CI, confidence interval. Random effects model used for analysis.

**
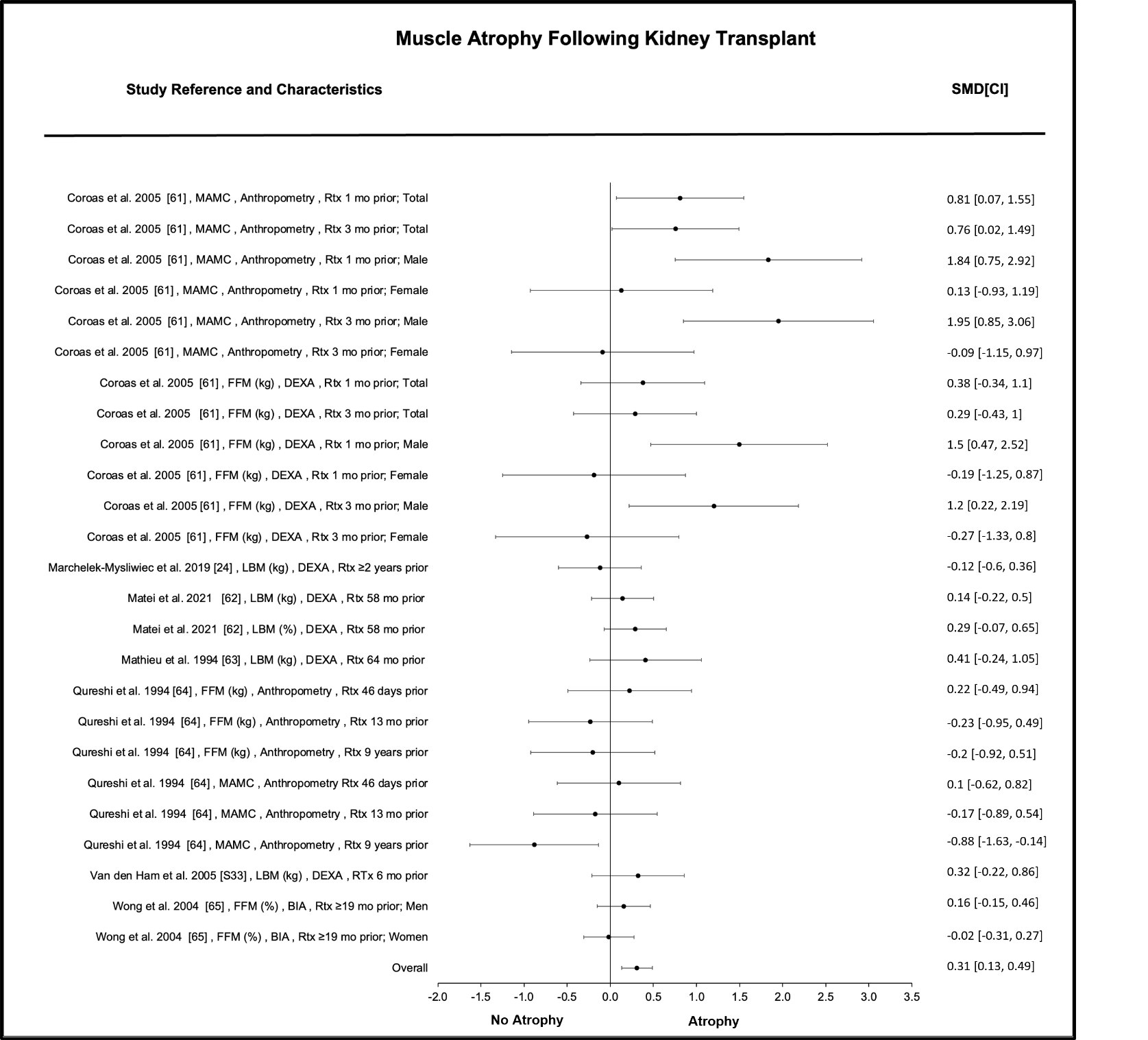
**

**Figure S14.** Muscle Atrophy Following Kidney Transplant in Recipients. SMD, standardized mean difference, CI, confidence interval. Random effects model used for analysis.


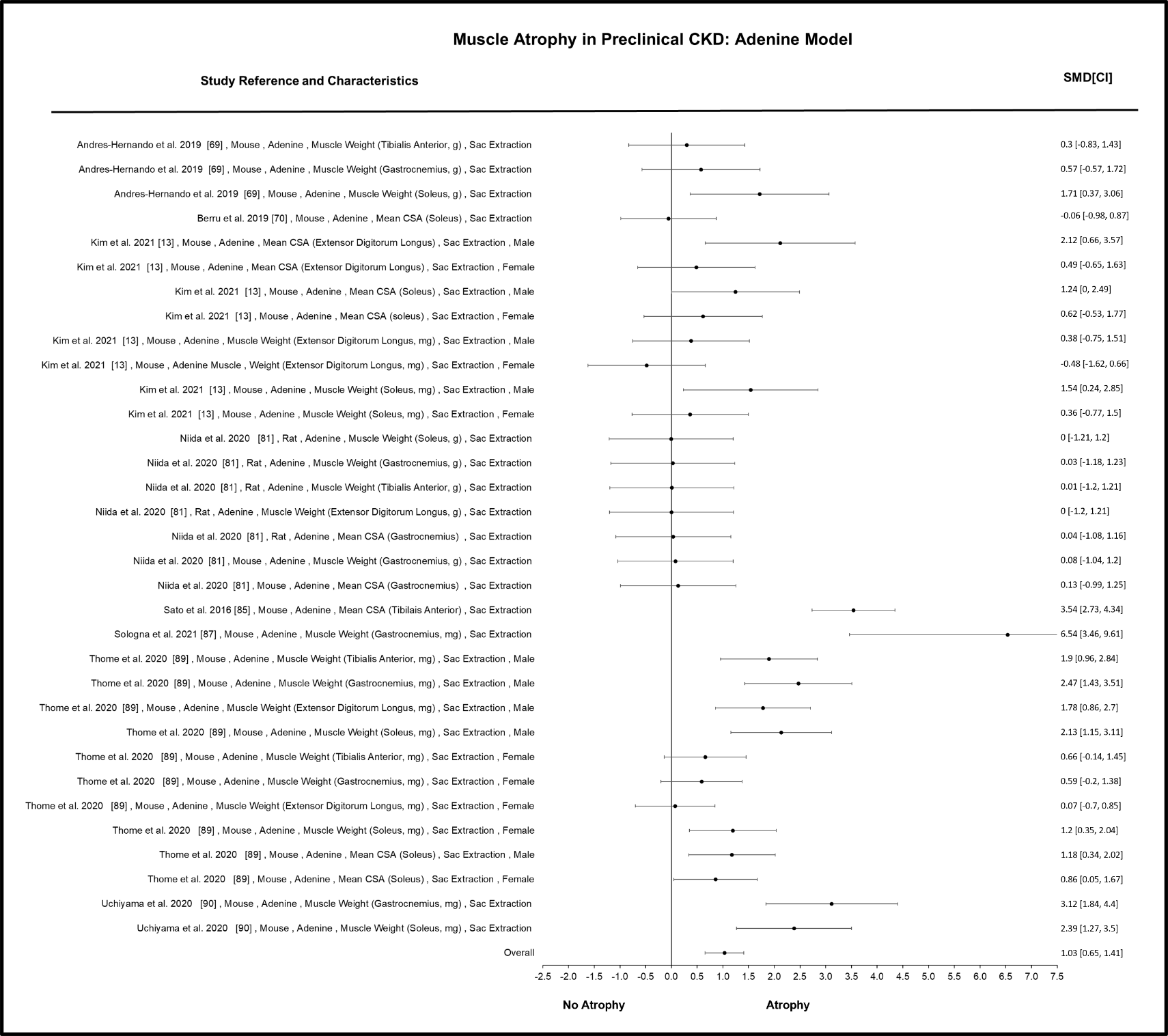


**Figure S15.** Muscle Atrophy in Preclinical CKD: Adenine Model. SMD, standardized mean difference, CI, confidence interval. Random effects model used for analysis.


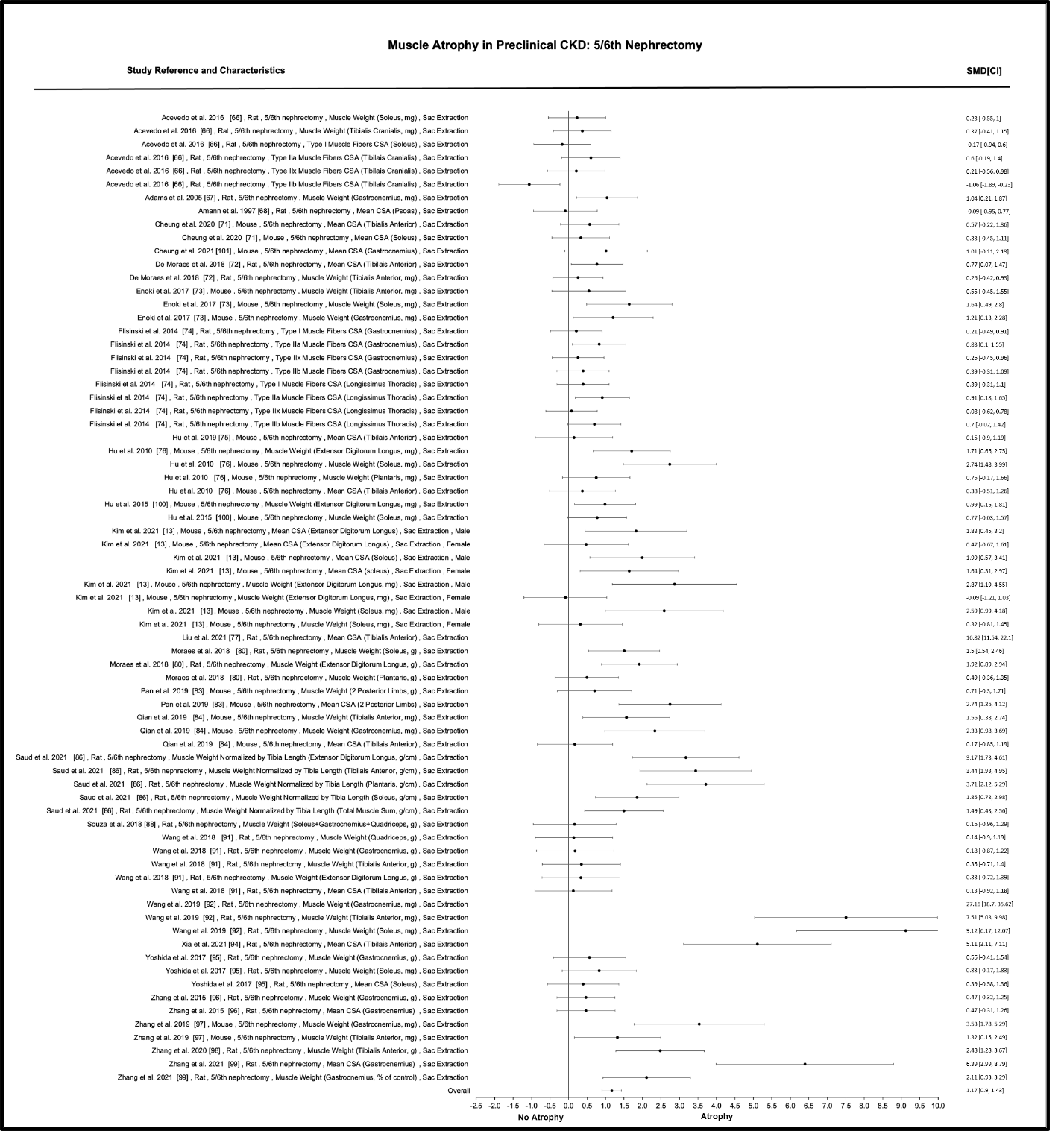


**Figure S16.** Muscle Atrophy in Preclinical CKD: 5/6 Nephrectomy Model. SMD, standardized mean difference, CI, confidence interval. Random effects model used for analysis.


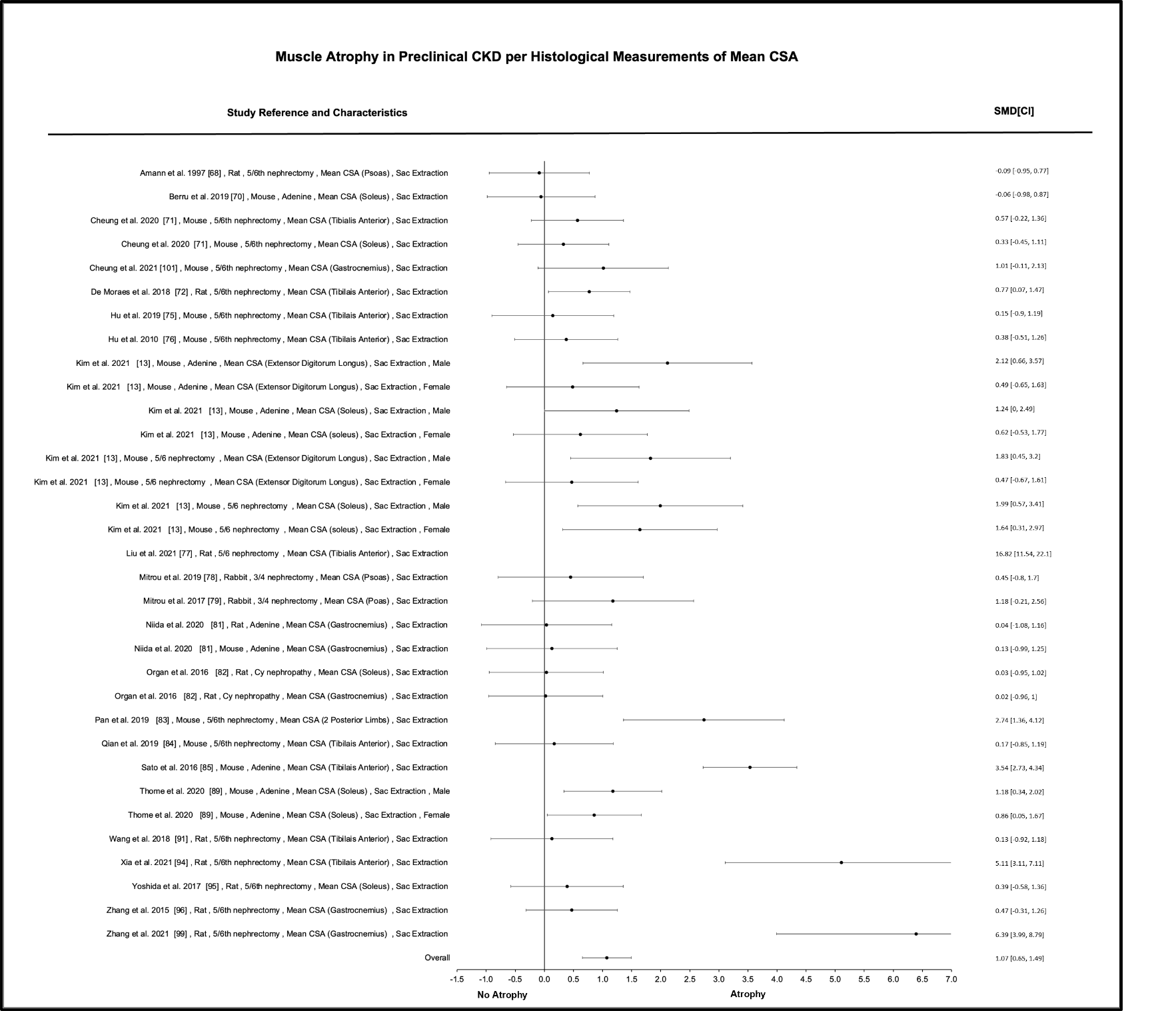


**Figure S17.** Muscle Atrophy in Preclinical CKD per Histological Mean CSA. SMD, standardized mean difference, CI, confidence interval. Random effects model used for analysis.


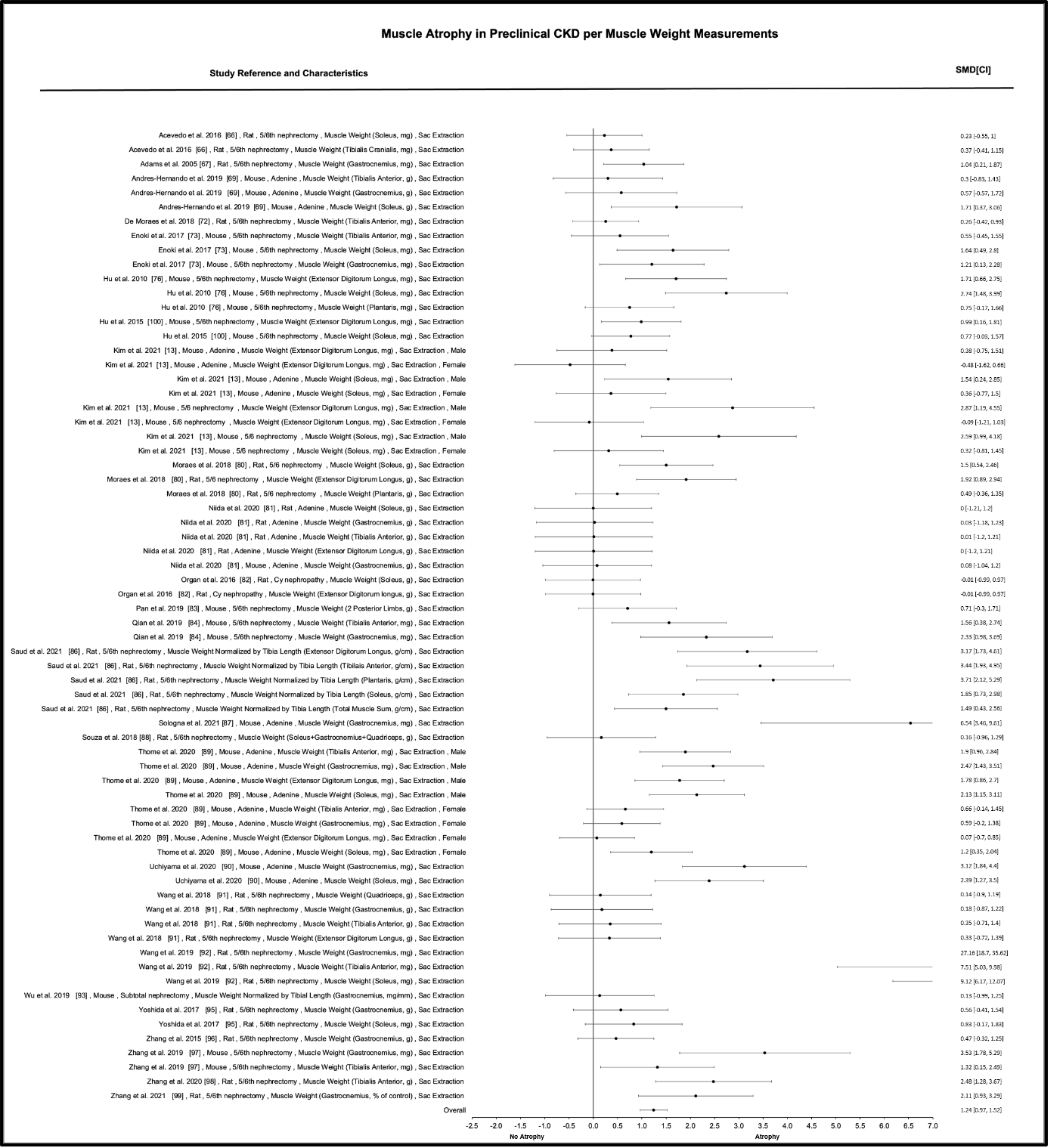


**Figure S18.** Muscle Atrophy in Preclinical CKD per Muscle Weight. SMD, standardized mean difference, CI, confidence interval. Random effects model used for analysis.


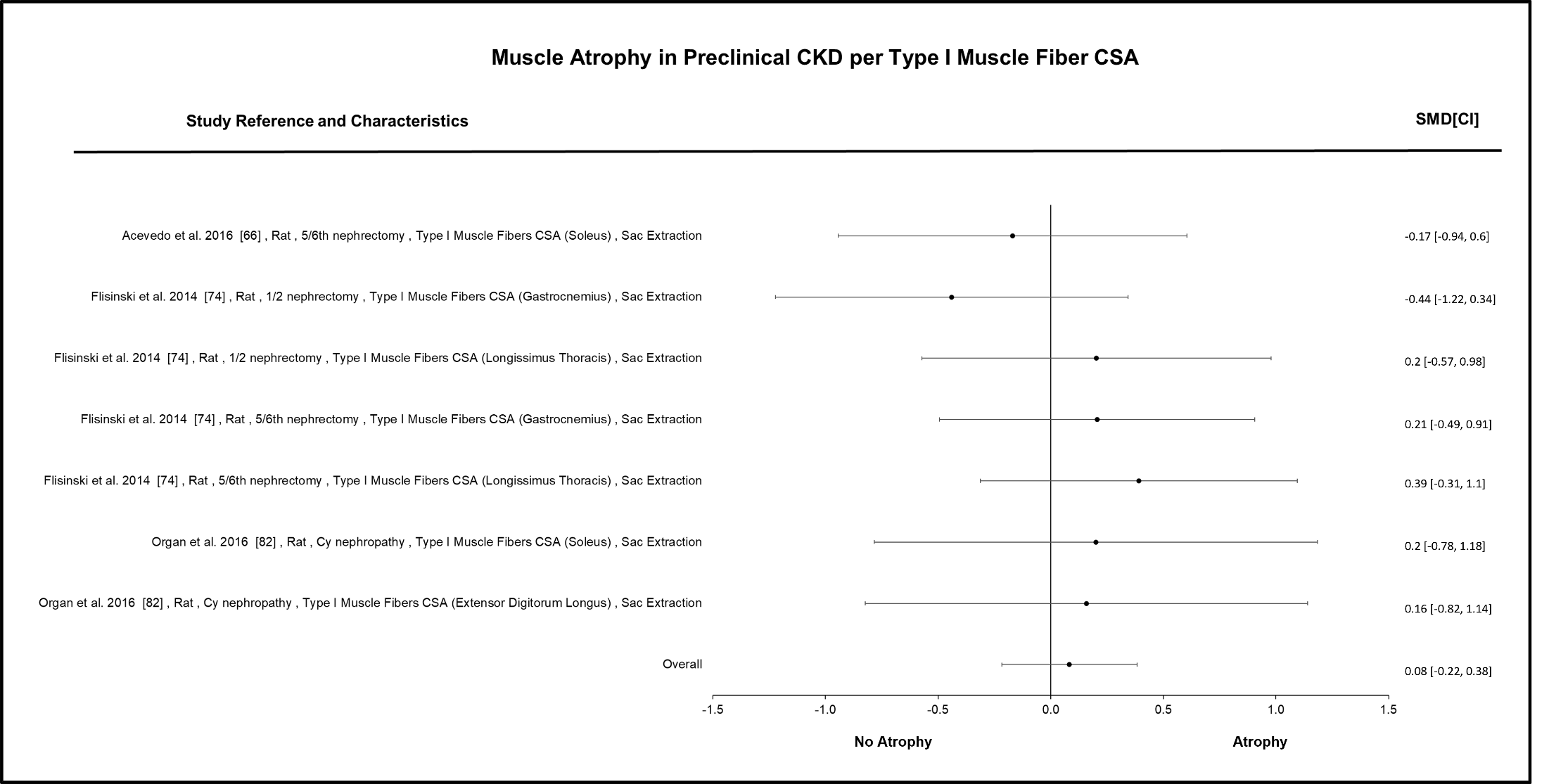


**Figure S19.** Muscle Atrophy in Preclinical CKD per Type I Muscle Fibers CSA. SMD, standardized mean difference, CI, confidence interval. Random effects model used for analysis.

**
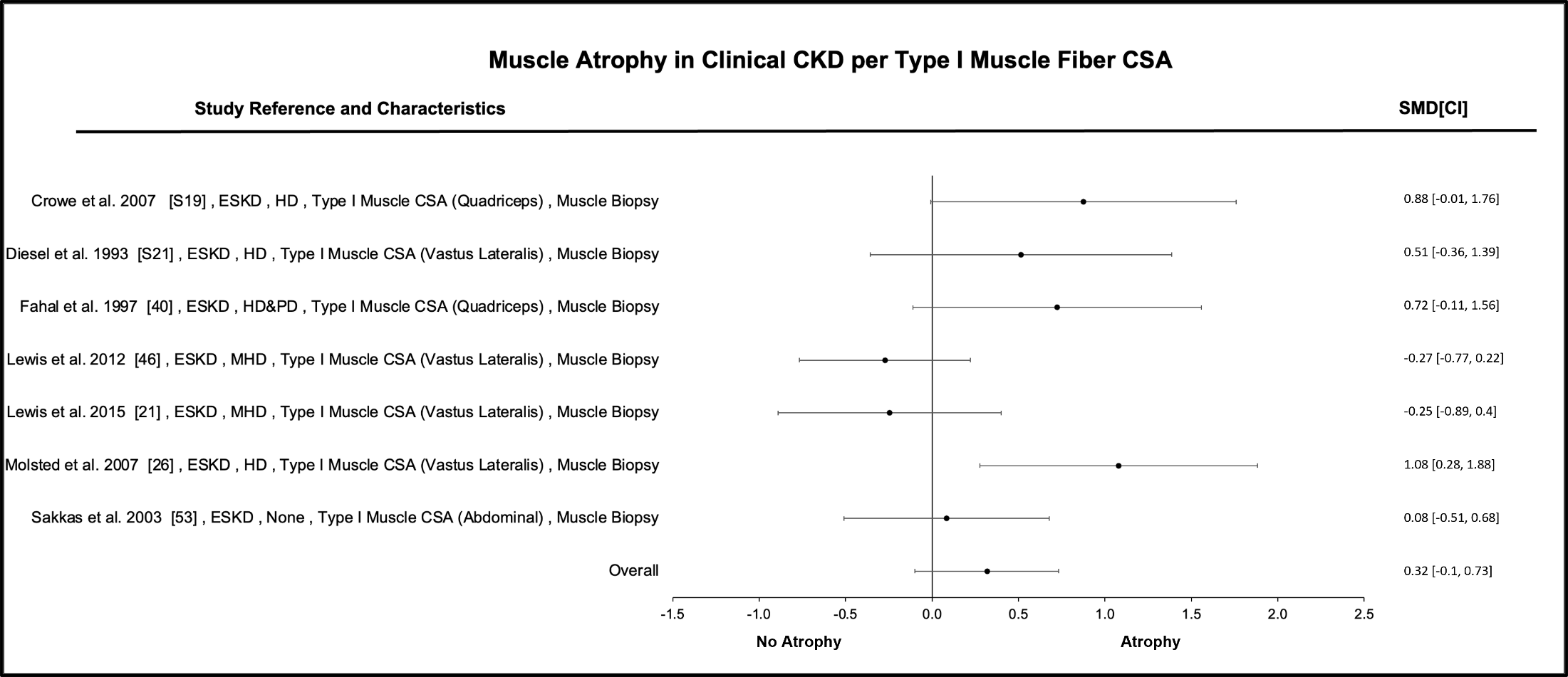
**

**Figure S20.** Muscle Atrophy in Clinical CKD per Type I Muscle Fiber CSA. SMD, standardized mean difference, CI, confidence interval. Random effects model used for analysis.


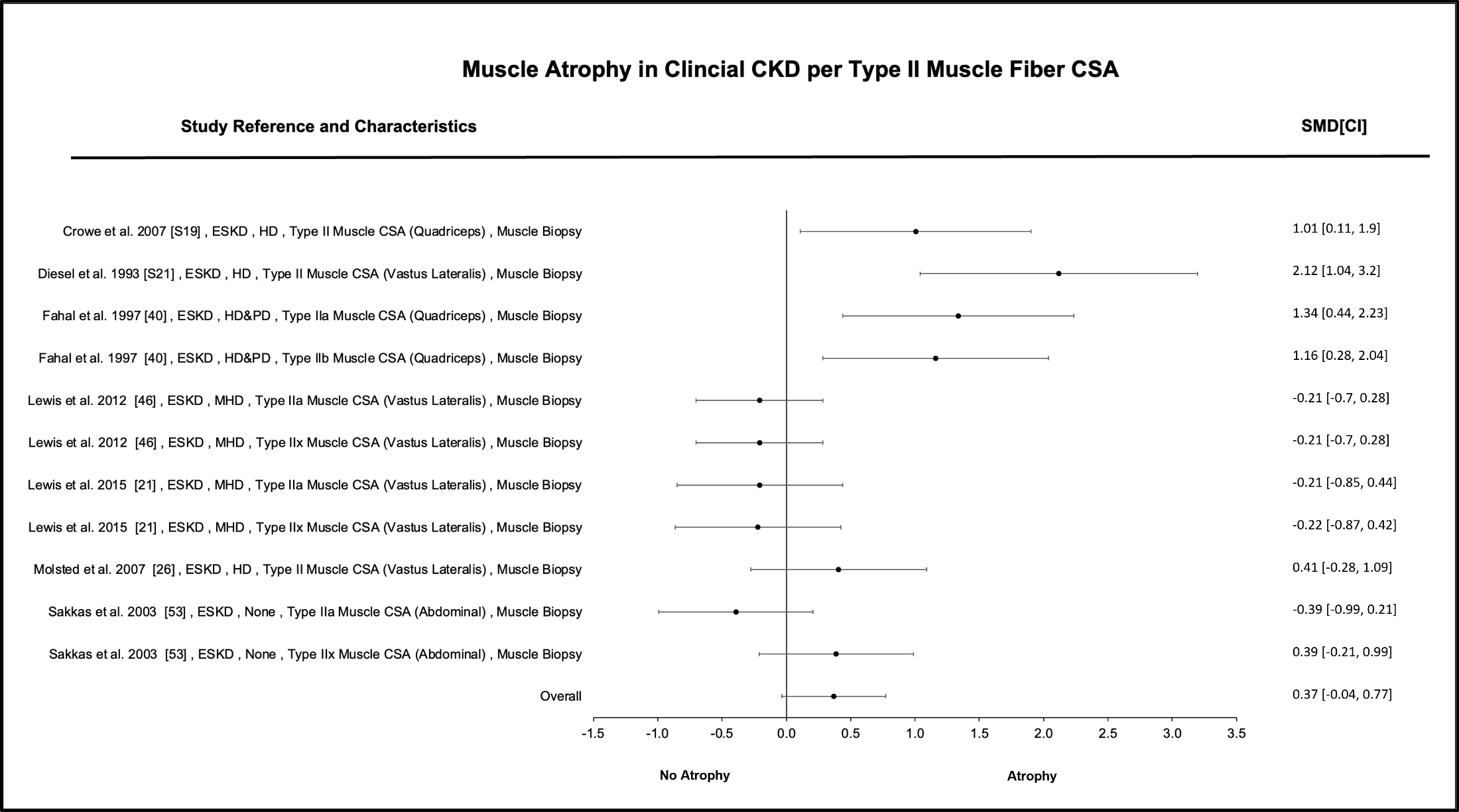

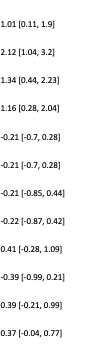


**Figure S21.** Muscle Atrophy in Clinical CKD per Type II Muscle Fiber CSA. SMD, standardized mean difference, CI, confidence interval. Random effects model used for analysis.


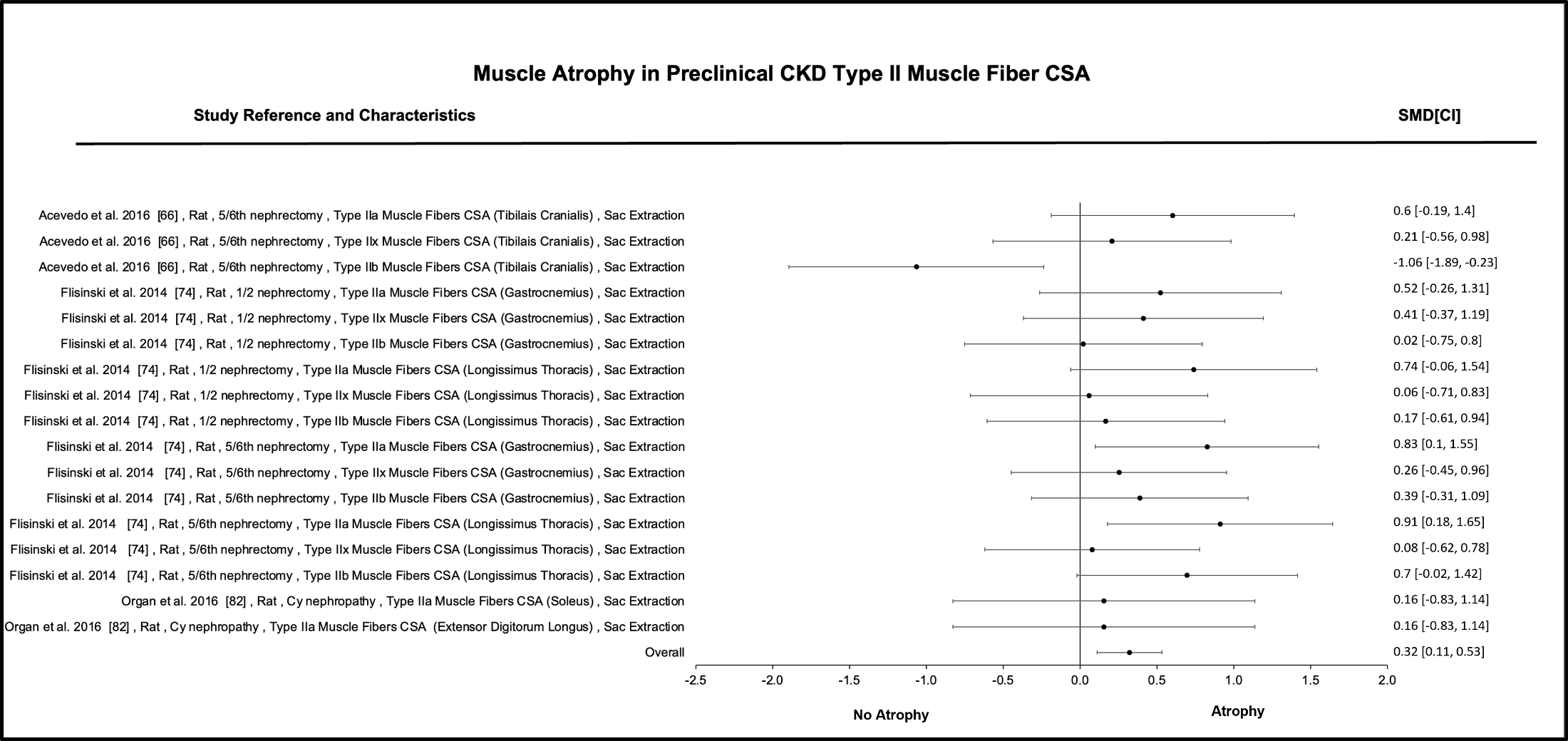


**Figure S22.** Muscle Atrophy in Preclinical CKD per Type II Muscle Fiber CSA. SMD, standardized mean difference, CI, confidence interval. Random effects model used for analysis.

**
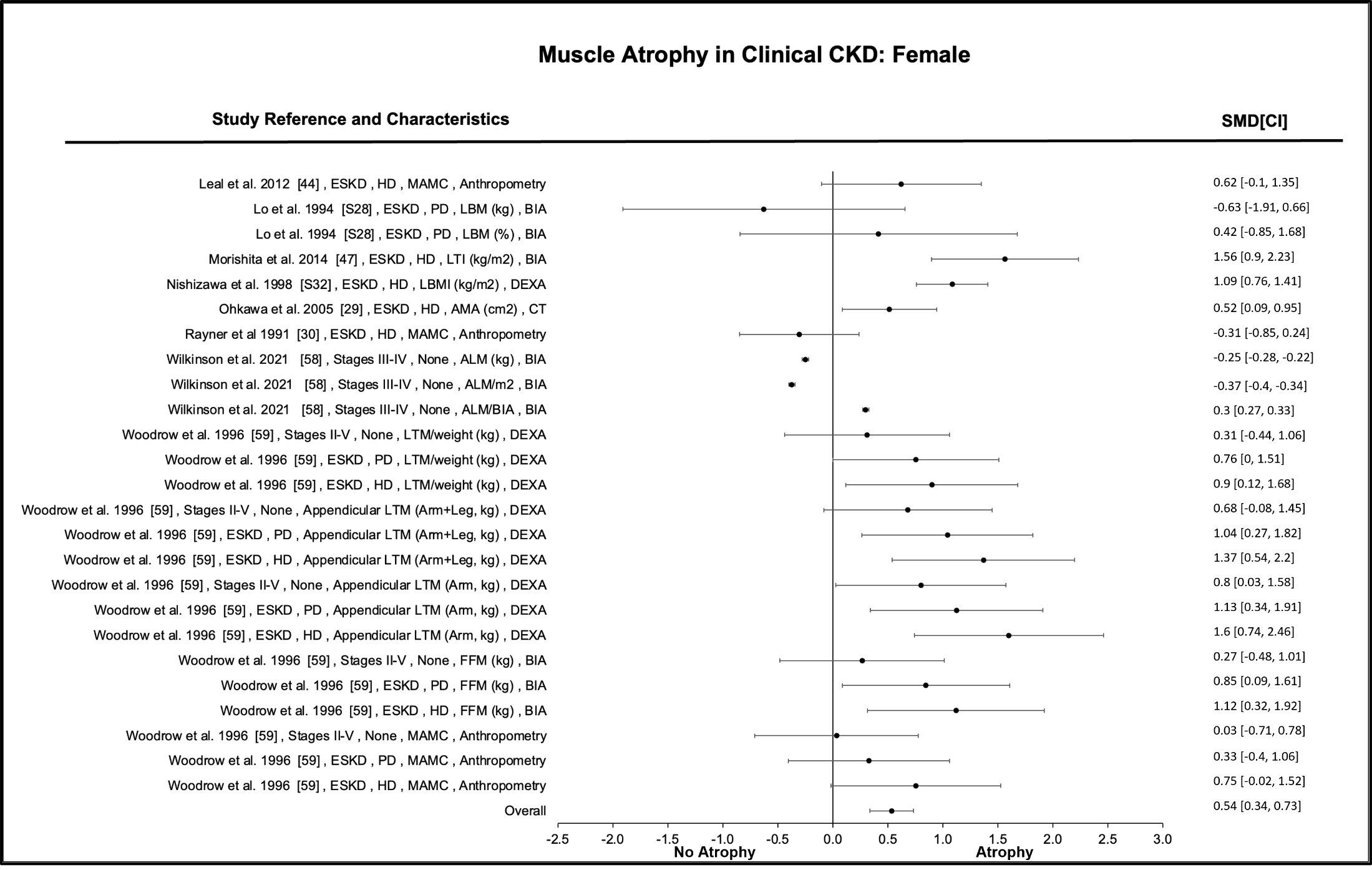
**

**Figure S23.** Muscle Atrophy in Clinical CKD: Females. SMD, standardized mean difference, CI, confidence interval. Random effects model used for analysis.

**
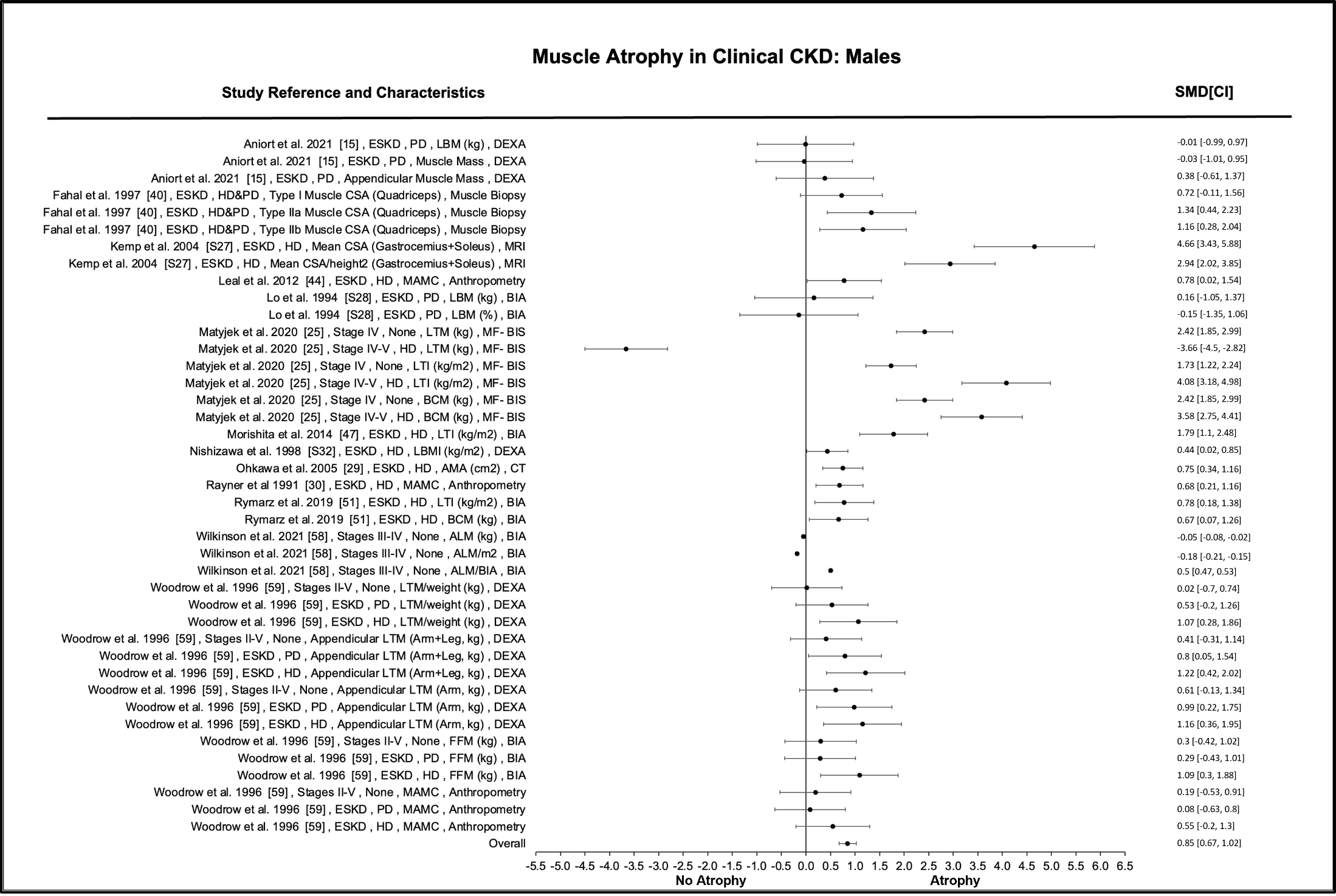
**

**Figure S24.** Muscle Atrophy in Clinical CKD: Males. SMD, standardized mean difference, CI, confidence interval. Random effects model used for analysis.

**
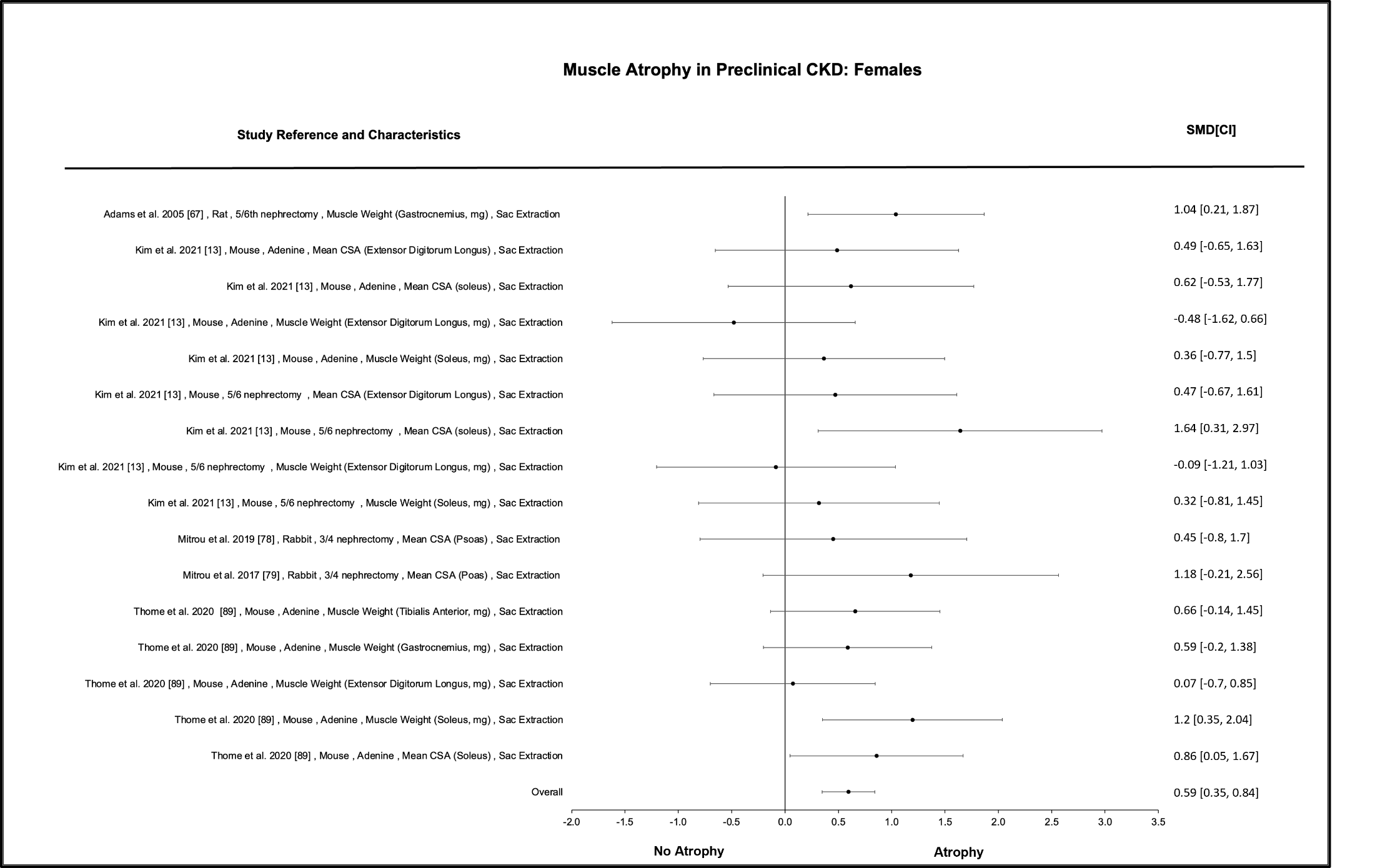
**

**Figure S25.** Muscle Atrophy in Preclinical CKD: Females. SMD, standardized mean difference, CI, confidence interval. Random effects model used for analysis.

**
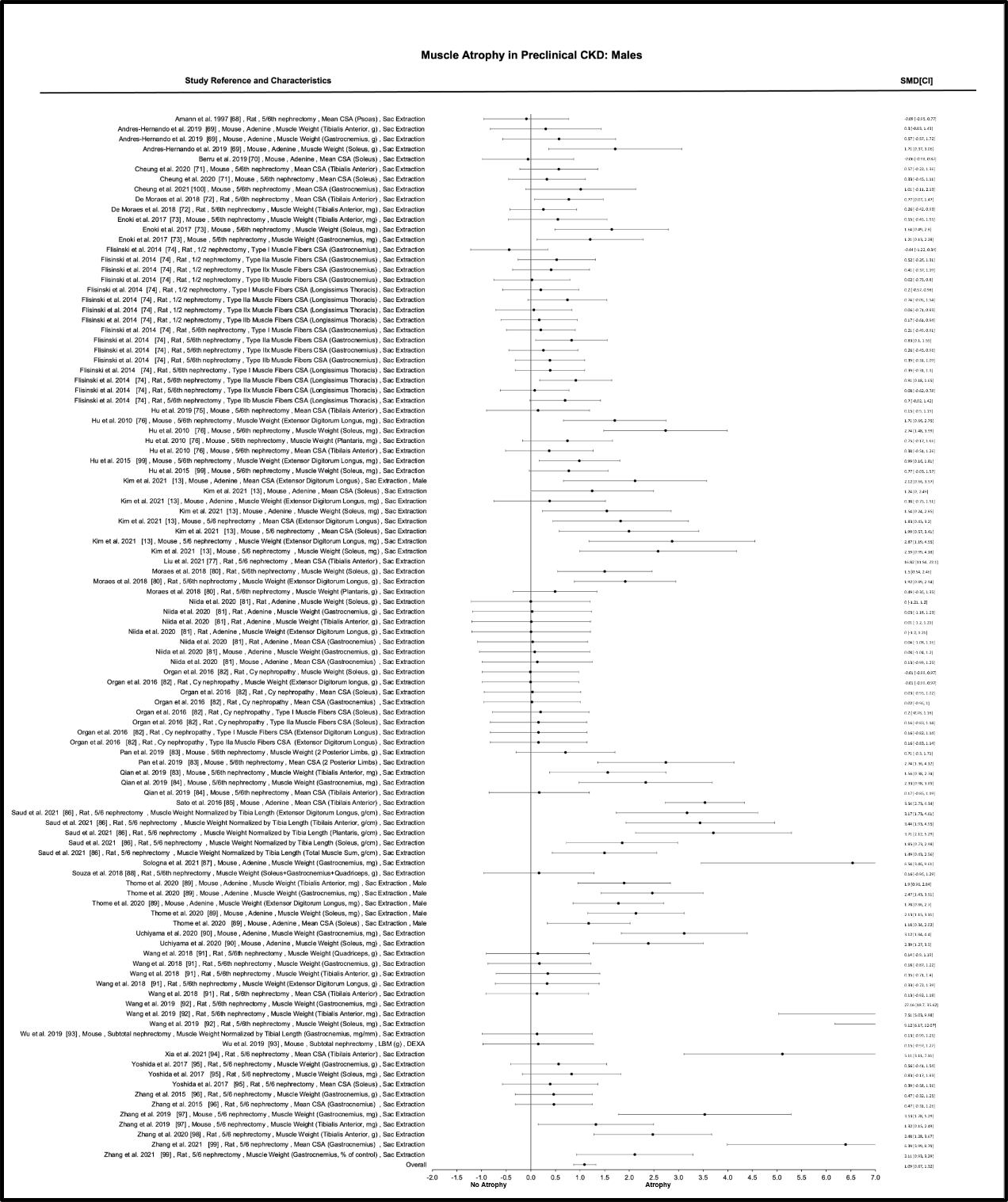
**

**Figure S26.** Muscle Atrophy in Preclinical CKD: Males. SMD, standardized mean difference, CI, confidence interval. Random effects model used for analysis.


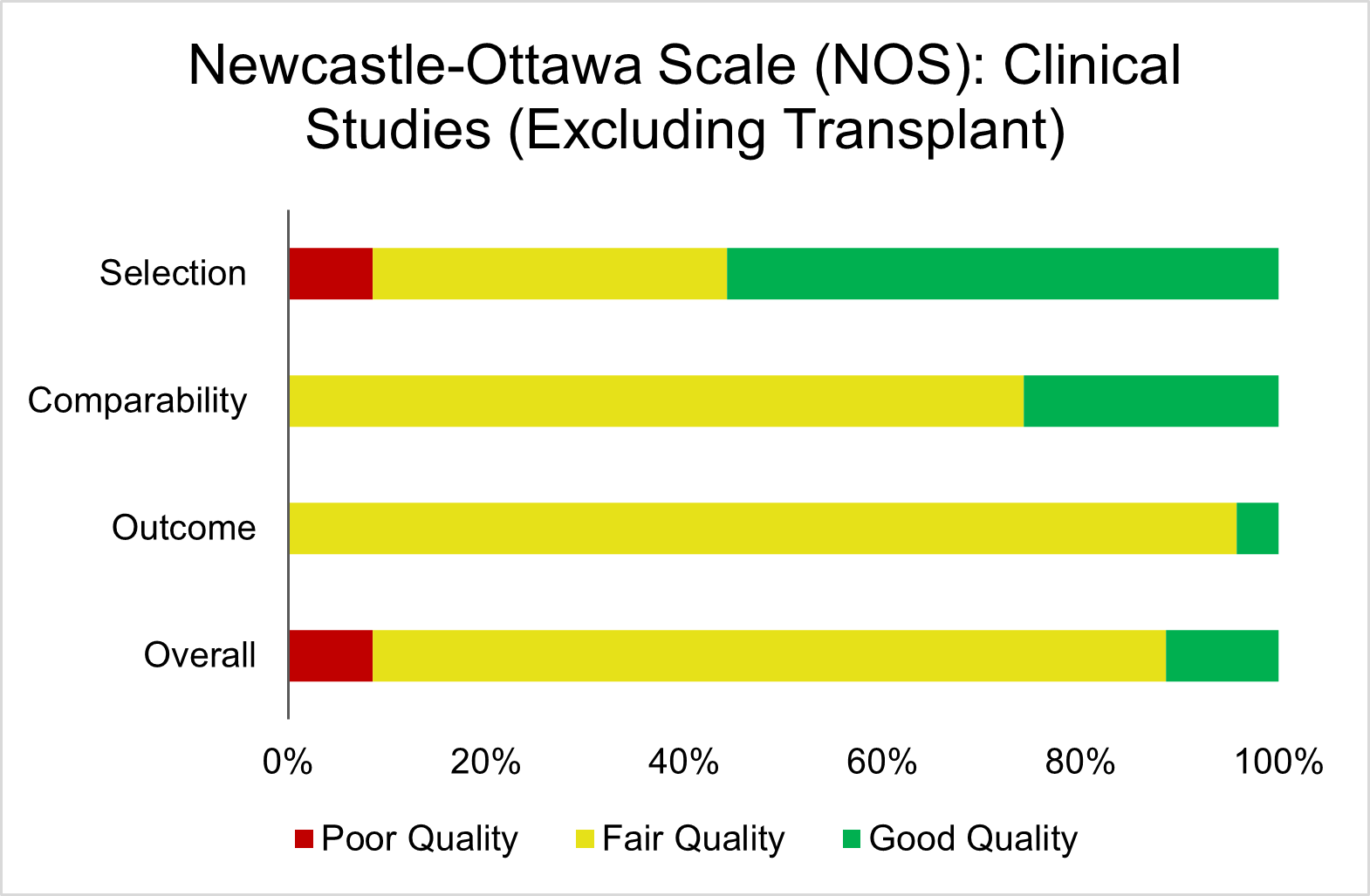


**Figure S27.** Quality Analysis of Clinical Studies (excluding transplant) using the NOS scale


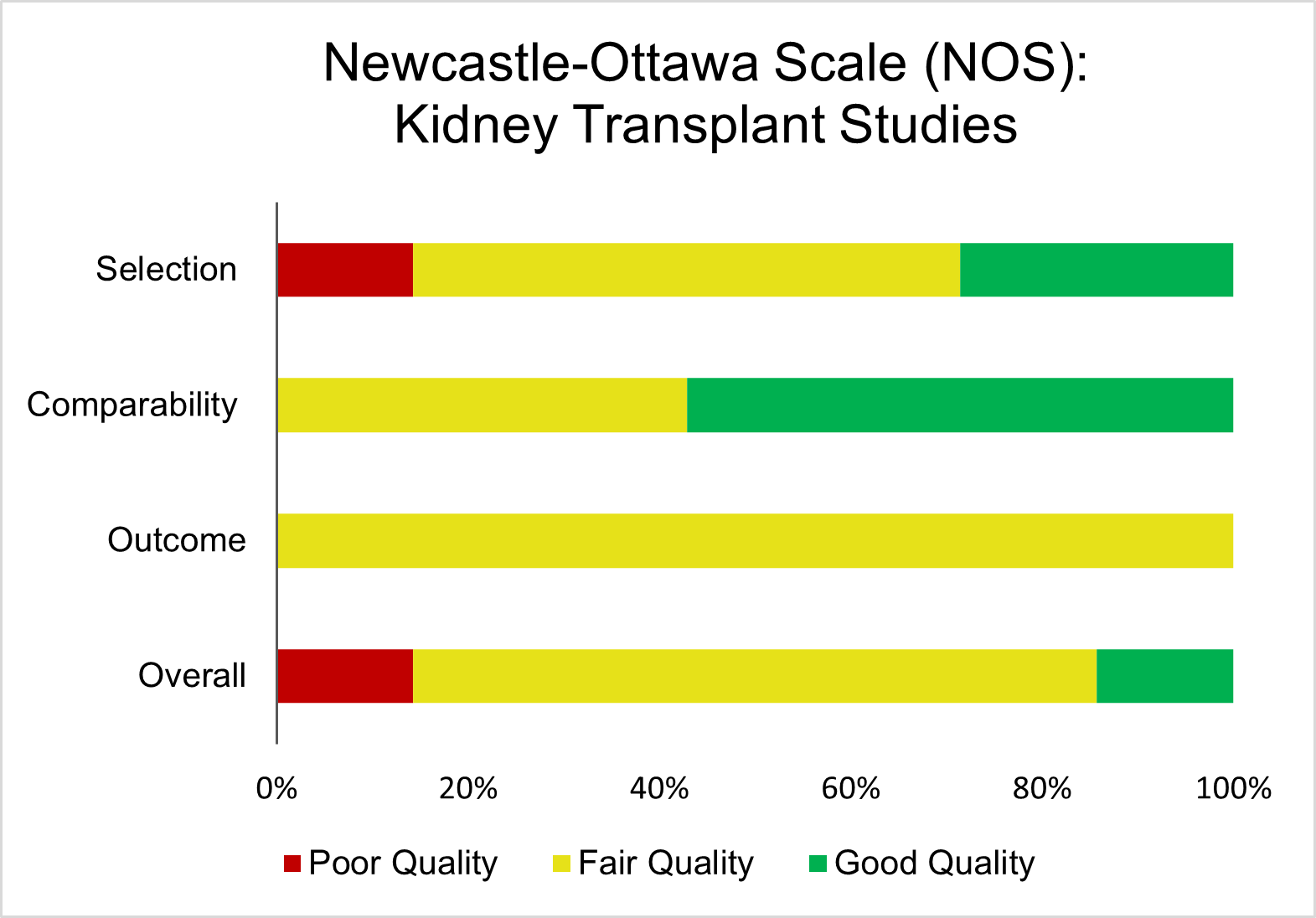


**Figure S28.** Quality Analysis of Transplant Studies using the NOS scale


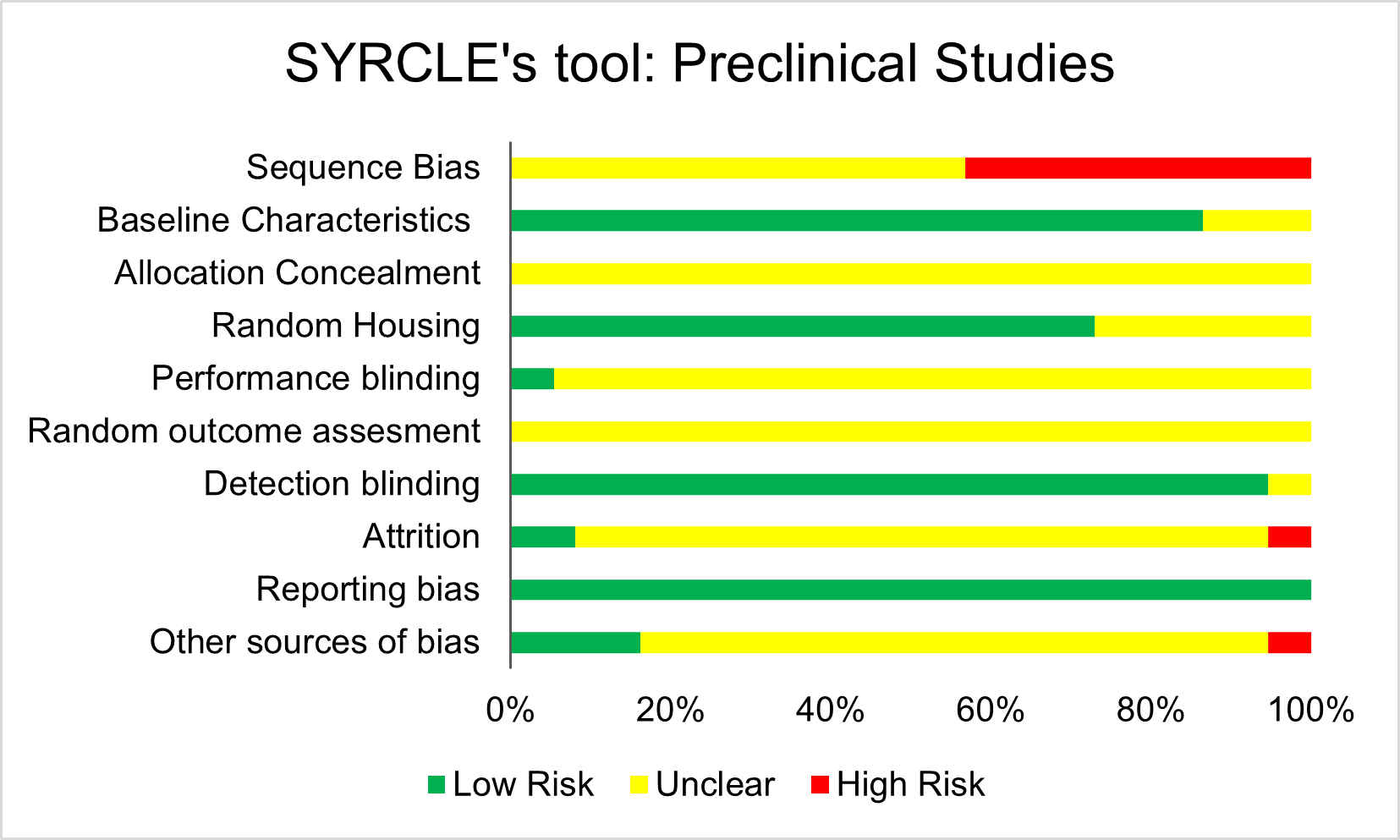


**Figure S29.** Quality Analysis of Preclinical Studies using the SYRCLE’s tool

| **Table S1. Characteristics of Clinical Studies** | | | | | | | | | | | | | | |
| --- | --- | --- | --- | --- | --- | --- | --- | --- | --- | --- | --- | --- | --- | --- |
| **Study** | **Disease Stage** | **Diabetes** | **Dialysis** | **Additional Defining Variable** | **Muscle** | **Measurement** | **Measurement Method** | **Controls**  **(N)** | **Age** | **SD** | **CKD**  **(N)** | **Age** | **SD** | **Significant Atrophy**  **(p< 0.05)** |
| Abramowitz et al. 2018 [35] | IV-V | Yes | None |  | Whole Body | LBM (kg) | DEXA | 10 | 61 | 15 | 10 | 62 | 8 | No |
| Adey et al. 2000 [14] | III-IV | No | None |  | Whole Body | FFM (kg) | DEXA | 10 | 41 | 10 | 12 | 43 | 9 | No |
| Akdam et al. 2016 [36] | ESRD | Yes | PD |  | Whole Body | LTI (kg/m^2^) | BIA | 37 | 50 | 14 | 38 | 52 | 16 | Yes |
| Akdam et al. 2016 [36] | ESRD | Yes | PD |  | Whole Body | BCM/Weight (%) | BIA | 37 | 50 | 14 | 38 | 52 | 16 | Yes |
| Akdam et al. 2016 [36] | ESRD | Yes | PD |  | Whole Body | LTM/Weight (%) | BIA | 37 | 50 | 12 | 38 | 52 | 16 | Yes |
| Aniort et al. 2021 [15] | ESRD | No | PD |  | Whole Body | LBM (kg) | DEXA | 7 | 53 | 6 | 7 | 50 | 16 | No |
| Aniort et al. 2021 [15] | ESRD | No | PD |  | Whole Body | Muscle Mass (kg) | DEXA | 7 | 53 | 6 | 7 | 50 | 16 | No |
| Aniort et al. 2021 [15] | ESRD | No | PD |  | Appendicular | LBM (kg) | DEXA | 7 | 53 | 6 | 7 | 50 | 16 | No |
| Agarwal et al. 2010 [S36] | III-V | Yes | None |  | Whole Body | LBM (kg) | Plesmography | 20 | 60 | 10 | 77 | 67 | 11 | No |
| Avesani et al. 2004 [37] | II-V | No | None |  | Whole Body | LBM (kg) | BIA | 45 | 45 | 12 | 45 | 45 | 12 | No |
| Avesani et al. 2004 [37] | II-V | No | None |  | Whole Body | LBM (%) | BIA | 45 | 45 | 12 | 45 | 45 | 12 | No |
| Axelsson et al. 2006 [S37] | III-IV | Yes | None |  | Whole Body | LBM (kg) | DEXA | 24 | 59 | 10 | 239 | 53 | 15 | No |
| Axelsson et al. 2006 [S37] | ESRD | Yes | None |  | Whole Body | LBM (kg) | DEXA | 24 | 59 | 10 | 239 | 53 | 15 | No |
| Bazanelli et al. 2006 [S16] | ESRD | Yes | PD |  | Whole Body | LBM (kg) | DEXA | 37 | 44 | 13 | 37 | 45 | 13 | Yes |
| Bazanelli et al. 2006 [S16] | ESRD | Yes | PD |  | Whole Body | LBM (kg) | DEXA | 37 | 44 | 13 | 37 | 45 | 13 | No |
| Baria et al. 2011 [38] | ESRD | Yes | HD |  | Whole Body | LBM (kg) | DEXA | 22 | 46 | 15 | 22 | 46 | 14 | No |
| Battaglia et al. 2020 [39] | ESRD | Yes | HD |  | Quadriceps | Muscle Thickness (cm) | US | 33 | 47 | 8 | 65 | 69 | 14 | Yes |
| Boivin et al. 2010 [S17] | ESRD | Yes | HD |  | Whole Body | LBM (kg) | DEXA | 6 | 45 | 12 | 8 | 43 | 17 | No |
| Bucar-Pajek et al. 2016 [16] | ESRD | Yes | HD |  | Whole Body | LTI (kg/m^2^) | BIA | 140 | 52 | 16 | 90 | 55 | 16 | No |
| Bucar-Pajek et al. 2016 [16] | ESRD | Yes | HD |  | Whole Body | LBM (kg) | BIA | 140 | 52 | 16 | 90 | 55 | 16 | No |
| Bueno et al. 2017 [17] | ESRD | Yes | HD |  | Pectoralis | Muscle Thickness (mm) | US | 17 | 48 | 15 | 17 | 54 | 14 | Yes |
| Bueno et al. 2017 [17] | ESRD | Yes | HD |  | Abdominals | Muscle Thickness (mm) | US | 17 | 48 | 15 | 17 | 54 | 14 | Yes |
| Chen et al. 2000 [S18] | ESRD | No | HD |  | Whole Body | LTM/weight (%) | DEXA | 40 | 54 | 9 | 40 | 54 | 9 | No |
| Coroas et al. 2005 [61] | Transplant | No | None | 1 mo post Rtx | Arm | MAMC (cm) | Anthropometry | 12 | 36 | 8.2 | 18 | 36 | 10.9 | Yes |
| Coroas et al. 2005 [61] | Transplant | No | None | 3 mo post Rtx | Arm | MAMC (cm) | Anthropometry | 12 | 36 | 8.2 | 18 | 36 | 10.9 | No |
| Coroas et al. 2005 [61] | Transplant | No | None | 1 mo post Rtx Male | Arm | MAMC (cm) | Anthropometry | 7 | 36 | 8.2 | 11 | 36 | 10.9 | Yes |
| Coroas et al. 2005 [61] | Transplant | No | None | 1 mo post Rtx Female | Arm | MAMC (cm) | Anthropometry | 5 | 36 | 8.2 | 7 | 36 | 10.9 | No |
| Coroas et al. 2005 [61] | Transplant | No | None | 3 mo post Rtx Male | Arm | MAMC (cm) | Anthropometry | 7 | 36 | 8.2 | 11 | 36 | 10.9 | Yes |
| CKD= chronic kidney disease; SD= standard deviation; MAMC= mean arm muscle circumference; LBM= lean body mass; FFM= fat free mass; BCM= body cell mass; LTI= lean tissue index; LTM= lean tissue mass; DEXA= dual energy x-ray absorptiometry; HD= hemodialysis; PD= peritoneal dialysis; ESRD= end-stage renal disease; US= ultrasound; BIA= bioelectrical impedance analysis; Rtx= transplant; mo= months. | | | | | | | | | | | | | | |
|  | | | | | | | | | | | | | | |
| **Table S1 continued. Characteristics of Clinical Studies** | | | | | | | | | | | | | | |
| **Study** | **Disease Stage** | **Diabetes** | **Dialysis** | **Additional Defining Variable** | **Muscle** | **Measurement** | **Measurement Method** | **Controls**  **(N)** | **Age** | **SD** | **CKD**  **(N)** | **Age** | **SD** | **Significant Atrophy**  **(p< 0.05)** |
| Coroas et al. 2005 [61] | Transplant | No | None | 3 mo post Rtx Female | Arm | MAMC (cm) | Anthropometry | 5 | 36 | 8.2 | 7 | 36 | 10.9 | No |
| Coroas et al. 2005 [61] | Transplant | No | None | 1 mo post Rtx | Whole Body | FFM (kg) | Anthropometry | 12 | 36 | 8.2 | 18 | 36 | 10.9 | No |
| Coroas et al. 2005 [61] | Transplant | No | None | 3 mo post Rtx | Whole Body | FFM (kg) | Anthropometry | 12 | 36 | 8.2 | 18 | 36 | 10.9 | No |
| Coroas et al. 2005 [61] | Transplant | No | None | 1 mo post Rtx Male | Whole Body | FFM (kg) | Anthropometry | 7 | 36 | 8.2 | 11 | 36 | 10.9 | Yes |
| Coroas et al. 2005 [61] | Transplant | No | None | 1 mo post Rtx Female | Whole Body | FFM (kg) | Anthropometry | 5 | 36 | 8.2 | 7 | 36 | 10.9 | No |
| Coroas et al. 2005 [61] | Transplant | No | None | 3 mo post Rtx Male | Whole Body | FFM (kg) | Anthropometry | 7 | 36 | 8.2 | 11 | 36 | 10.9 | Yes |
| Coroas et al. 2005 [61] | Transplant | No | None | 3 mo post Rtx Female | Whole Body | FFM (kg) | Anthropometry | 5 | 36 | 8.2 | 7 | 36 | 10.9 | No |
| Crowe et al. 2007 [S19] | ESRD | No | HD |  | Quadriceps | Type I CSA (μm) | Biopsy | 10 | 35 | 19 | 10 | 49 | 25 | Yes |
| Crowe et al. 2007 [S19] | ESRD | No | HD |  | Quadriceps | Type II CSA (μm) | Biopsy | 10 | 35 | 19 | 10 | 49 | 25 | Yes |
| Cuptsi et al. 2004 [S20] | ESRD | No | None |  | Arm | MAMC (mm) | Anthropometry | 28 | 45 | 8 | 28 | 45 | 11 | No |
| Cuptsi et al. 2004 [S20] | ESRD | No | None |  | Whole Body | BCM (kg/m^2^) | BIA | 28 | 45 | 8 | 28 | 45 | 11 | No |
| Diesel et al. 1993 [S21] | ESRD | Yes | HD |  | Vastus Lateralis | Type I CSA (μm) | Biopsy | 12 | 20-30 | NA | 8 | 34 | 12 | No |
| Diesel et al. 1993 [S21] | ESRD | Yes | HD |  | Vastus Lateralis | Type II CSA (μm) | Biopsy | 12 | 20-30 | NA | 8 | 34 | 12 | Yes |
| Elsayed et al. 2009 [S22] | ESRD | Yes | HD |  | Whole Body | LBM (gm) | DEXA | 20 | 43 | 12 | 23 | 42 | 13 | Yes |
| Elsayed et al. 2009 [S22] | ESRD | Yes | Pre-HD |  | Whole Body | LBM (gm) | DEXA | 20 | 43 | 12 | 20 | 43 | 13 | No |
| Fahal et al. 1997 [40] | ESRD | Yes | HD&PD |  | Quadriceps | Type I CSA (mm^2^) | Biopsy | 10 | 37 | 12 | 12 | 42 | 15 | No |
| Fahal et al. 1997 [40] | ESRD | Yes | HD&PD |  | Quadriceps | Type IIa CSA (mm^2^) | Biopsy | 10 | 37 | 12 | 12 | 42 | 15 | No |
| Fahal et al. 1997 [40] | ESRD | Yes | HD&PD |  | Quadriceps | Type IIb CSA (mm^2^) | Biopsy | 10 | 37 | 12 | 12 | 42 | 15 | No |
| Fisch et al. 1996 [18] | ESRD | Yes | Post-HD |  | Whole Body | LBM (kg) | DEXA | 16 | 35 | 5 | 11 | 42 | 14 | No |
| Fisch et al. 1996 [18] | ESRD | Yes | Pre-HD |  | Whole Body | LBM (kg) | DEXA | 16 | 35 | 5 | 11 | 42 | 14 | No |
| Guida et al. 2001 [S23] | ESRD | Yes | HD | BMI 18.5.-24.9 | Whole Body | FFM (kg) | BIA | 14 | 63 | 6 | 27 | 58 | 11 | No |
| Guida et al. 2001 [S23] | ESRD | Yes | HD | BMI 18.5.-24.9 | Whole Body | FFM (kg) | Anthropometry | 14 | 63 | 6 | 27 | 58 | 11 | No |
| Guida et al. 2001 [S23] | ESRD | Yes | HD | BMI 18.5.-24.9 | Whole Body | FFM (%) | BIA | 14 | 63 | 6 | 27 | 58 | 11 | No |
| Guida et al. 2001 [S23] | ESRD | Yes | HD | BMI 18.5.-24.9 | Whole Body | FFM (%) | Anthropometry | 14 | 63 | 6 | 27 | 58 | 11 | No |
| Guida et al. 2001 [S23] | ESRD | Yes | HD | BMI 25-29.9 | Whole Body | FFM (kg) | BIA | 12 | 63 | 6 | 16 | 58 | 11 | Yes |
| CKD= chronic kidney disease; SD= standard deviation; MAMC= mean arm muscle circumference; CSA= cross-sectional area; LBM= lean body mass; FFM= fat free mass; BMI= body mass index; BCM= body cell mass; HD= hemodialysis; PD= peritoneal dialysis; ESRD= end-stage renal disease; BIA= bioelectrical impedance analysis. Pre-HD: measurement taken prior to dialysis. Post-HD: measurement taken after to dialysis; DEXA= dual energy x-ray absorptiometry. | | | | | | | | | | | | | | |
| **Table S1 continued. Characteristics of Clinical Studies** | | | | | | | | | | | | | | |
| **Study** | **Disease Stage** | **Diabetes** | **Dialysis** | **Additional Defining Variable** | **Muscle** | **Measurement** | **Measurement Method** | **Controls**  **(N)** | **Age** | **SD** | **CKD**  **(N)** | **Age** | **SD** | **Significant Atrophy**  **(p< 0.05)** |
| Guida et al. 2001 [S23] | ESRD | Yes | HD | BMI 25-29.9 | Whole Body | FFM (kg) | Anthropometry | 12 | 63 | 6 | 16 | 58 | 11 | No |
| Guida et al. 2001 [S23] | ESRD | Yes | HD | BMI 25-29.9 | Whole Body | FFM (%) | BIA | 12 | 63 | 6 | 16 | 58 | 11 | Yes |
| Guida et al. 2001 [S23] | ESRD | Yes | HD | BMI 25-29.9 | Whole Body | FFM (%) | Anthropometry | 12 | 63 | 6 | 16 | 58 | 11 | No |
| Guida et al. 2001 [S23] | ESRD | Yes | HD | BMI > 30 | Whole Body | FFM (kg) | BIA | 12 | 63 | 6 | 7 | 58 | 11 | Yes |
| Guida et al. 2001 [S23] | ESRD | Yes | HD | BMI > 30 | Whole Body | FFM (kg) | Anthropometry | 12 | 63 | 6 | 7 | 58 | 11 | Yes |
| Guida et al. 2001 [S23] | ESRD | Yes | HD | BMI > 30 | Whole Body | FFM (%) | BIA | 12 | 63 | 6 | 7 | 58 | 11 | Yes |
| Guida et al. 2001 [S23] | ESRD | Yes | HD | BMI > 30 | Whole Body | FFM (%) | Anthropometry | 12 | 63 | 6 | 7 | 58 | 11 | Yes |
| Han et al. 2011 [19] | ESRD | Yes | HD |  | Whole Body | Muscle Mass (kg) | BIA | 41 | 63 | 16 | 60 | 64 | 10 | No |
| Isoyama et al. 2016 [41] | ESRD | Yes | PD |  | Whole Body | LBM (kg/m^2^) | DEXA | 50 | 63 | 3 | 82 | 65 | 5 | No |
| Jairam et al. 2010 [S24] | ESRD | Yes | HD&PD |  | Arm | MAMC (cm) | Anthropometry | 52 | 40 | 9 | 74 | 36 | 12 | Yes |
| Johansen et al. 1998 [S25] | ESRD | Yes | MHD |  | Whole Body | LBM (g/m) | DEXA | 41 | 36 | 10 | 19 | 48 | 12 | No |
| Johansen et al. 1998 [S25] | ESRD | Yes | PD |  | Whole Body | LBM (g/m) | DEXA | 41 | 36 | 10 | 9 | 43 | 15 | No |
| Johansen et al. 2003 [6] | ESRD | Yes | HD |  | Tibialis Anterior | Mean CSA (cm^2^) | MRI | 17 | 55 | 13 | 22 | 55 | 15 | Yes |
| Kamimura et al. 2007 [S26] | ESRD | No | HD |  | Whole Body | LBM (%) | BIA | 11 | 37 | 15 | 30 | 47 | 16 | No |
| Kamimura et al. 2007 [S26] | ESRD | No | HD |  | Whole Body | LBM (kg) | BIA | 11 | 37 | 15 | 30 | 47 | 16 | No |
| Kamimura et al. 2007 [S26] | ESRD | No | HD |  | Whole Body | FFM (kg) | BIA | 55 | 42 | 13 | 55 | 41 | 13 | Yes |
| Kemp et al. 2004 [S27] | ESRD | No | HD |  | Calf | Mean CSA (cm^2^) | MRI | 15 | 43 | NA | 23 | 50 | NA | Yes |
| Kemp et al. 2004 [S27] | ESRD | No | HD |  | Calf | Mean CSA/height^2^ | MRI | 15 | 43 | NA | 23 | 50 | NA | Yes |
| Kim et al. 2020 [20] | III | No | None |  | Whole Body | LBM (kg) | BIA | 102 | 76 | 4 | 98 | 77 | 4 | Yes |
| Kim et al. 2020 [20] | III-V | No | None |  | Whole Body | LBM (kg) | BIA | 102 | 76 | 4 | 36 | 78 | 3 | Yes |
| Kim et al. 2020 [20] | III | No | None |  | Appendicular | LBM (kg) | BIA | 102 | 76 | 4 | 98 | 77 | 4 | Yes |
| Kim et al. 2020 [20] | III-V | No | None |  | Appendicular | LBM (kg) | BIA | 102 | 76 | 4 | 36 | 78 | 3 | Yes |
| Kim et al. 2020 [20] | III | No | None |  | Whole Body | Muscle Mass (kg/m^2^) | BIA | 102 | 76 | 4 | 98 | 77 | 4 | Yes |
| Kim et al. 2020 [20] | III-V | No | None |  | Whole Body | Muscle Mass (kg/m^2^) | BIA | 102 | 76 | 4 | 36 | 78 | 3 | Yes |
| Kim et al. 2020 [20] | III | No | None |  | Whole Body | Muscle Mass (%) | BIA | 102 | 76 | 4 | 98 | 77 | 4 | Yes |
| Kim et al. 2020 [20] | III-V | No | None |  | Whole Body | Muscle Mass (%) | BIA | 102 | 76 | 4 | 36 | 78 | 3 | Yes |
| Kirkman et al. 2014 [43] | ESRD | Yes | HD |  | Thigh | Muscle Volume (cm^3^) | MRI | 8 | 45 | 16 | 19 | 53 | 12 | No |
| Leal et al. 2012 [44] | ESRD | Yes | HD | Male | Arm | MAMC (cm) | Anthropometry | 9 | 56 | 16 | 28 | 54 | 13 | No |
| Leal et al. 2012 [44] | ESRD | Yes | HD | Female | Arm | MAMC (cm) | Anthropometry | 11 | 46 | 14 | 21 | 53 | 13 | No |
| CKD= chronic kidney disease; SD= standard deviation; MAMC= mean arm muscle circumference; CSA= cross-sectional area; LBM= lean body mass; FFM= fat free mass; DEXA= dual energy x-ray absorptiometry; HD= hemodialysis; PD= peritoneal dialysis; ESRD= end-stage renal disease; BIA= bioelectrical impedance analysis; BMI= body mass index; MRI= magnetic resonance imaging. | | | | | | | | | | | | | | |
| **Table S1 continued. Characteristics of Clinical Studies** | | | | | | | | | | | | | | |
| **Study** | **Disease Stage** | **Diabetes** | **Dialysis** | **Additional Defining Variable** | **Muscle** | **Measurement** | **Measurement Method** | **Controls**  **(N)** | **Age** | **SD** | **CKD**  **(N)** | **Age** | **SD** | **Significant Atrophy**  **(p< 0.05)** |
| Lee et al. 2015 [45] | ESRD | Yes | PD |  | Arm | MAMC (cm) | Anthropometry | 35 | 54 | 12 | 102 | 54 | 12 | No |
| Lewis et al. 2012 [46] | ESRD | Yes | MHD |  | Vastus Lateralis | Type I CSA (μm^2^) | Biopsy | 21 | 41 | 12 | 60 | 44 | 11 | No |
| Lewis et al. 2012 [46] | ESRD | Yes | MHD |  | Vastus Lateralis | Type IIa CSA (μm^2^) | Biopsy | 21 | 41 | 12 | 60 | 44 | 11 | No |
| Lewis et al. 2012 [46] | ESRD | Yes | MHD |  | Vastus Lateralis | Type IIx CSA (μm^2^) | Biopsy | 21 | 41 | 12 | 60 | 44 | 11 | No |
| Lewis et al. 2015 [21] | ESRD | No | MHD |  | Vastus Lateralis | Type I CSA (μm^2^) | Biopsy | 22 | 41 | 3 | 15 | 42 | 2 | No |
| Lewis et al. 2015 [21] | ESRD | No | MHD |  | Vastus Lateralis | Type IIa CSA (μm^2^) | Biopsy | 22 | 41 | 3 | 15 | 42 | 2 | No |
| Lewis et al. 2015 [21] | ESRD | No | MHD |  | Vastus Lateralis | Type IIx CSA (μm^2^) | Biopsy | 22 | 41 | 3 | 15 | 42 | 2 | No |
| Lo et al. 1994 [S28] | ESRD | Yes | PD | Male | Whole Body | LBM (kg) | BIA | 4 | 47 | 9 | 4 | 49 | 7 | No |
| Lo et al. 1994 [S28] | ESRD | Yes | PD | Female | Whole Body | LBM (kg) | BIA | 3 | 39 | 1 | 5 | 45 | 13 | No |
| Lo et al. 1994 [S28] | ESRD | Yes | PD | Male | Whole Body | LBM (%) | BIA | 4 | 47 | 9 | 4 | 49 | 7 | No |
| Lo et al. 1994 [S28] | ESRD | Yes | PD | Female | Whole Body | LBM (%) | BIA | 3 | 39 | 1 | 5 | 45 | 13 | No |
| Macdonald et al. 2004 [S29] | ESRD | Yes | HD |  | Appendicular | LBM (kg/m^2^) | DEXA | 17 | 56 | 11 | 17 | 58 | 14 | Yes |
| Malgorzewicz et al. 2008 [S30] | ESRD | Yes | HD |  | Whole Body | LBM (kg) | NIR | 20 | 49 | 19 | 22 | 56 | 13 | No |
| Malgorzewicz et al. 2018 [23] | ESRD | Yes | PD |  | Whole Body | LBM (kg) | NIR | 23 | 63 | 8 | 30 | 57 | 18 | No |
| Malgorzewicz et al. 2010 [22] | ESRD | Yes | HD |  | Whole Body | LBM (kg) | NIR | 23 | 63 | 8 | 36 | 55 | 12 | No |
| Marchelek-Mysliwiec et al. 2019 [24] | II-IV | Yes | HD |  | Whole Body | LBM (kg) | DEXA | 23 | 54 | 13 | 47 | 59 | 15 | No |
| Marchelek-Mysliwiec et al. 2019 [24] | Transplant | No | None | >2 years post Rtx | Whole Body | LBM (kg) | DEXA | 23 | 53.8 | 13.2 | 56 | 55 | 12.4 | No |
| Matei et al. 2022 [62] | Transplant | No | None | 58 mo post Rtx | Whole Body | LBM (kg) | DEXA | 59 | 43.7 | 1.9 | 59 | 30 | 29 | No |
| Matei et al. 2022 [62] | Transplant | No | None | 58 mo post Rtx | Whole Body | LBM (%) | DEXA | 59 | 43.7 | 1.9 | 59 | 30 | 29 | No |
| Mathieu et al. 1994 [63] | Transplant | No | None | 64 mo post Rtx | Whole Body | LBM (kg) | DEXA | 18 | 50.6 | 2.2 | 18 | 51.8 | 2.7 | yes |
| Matyjek et al. 2020 [25] | IV | Yes | None |  | Whole Body | LTM (kg) | BIA | 40 | 48 | 18 | 40 | 59 | 15 | Yes |
| Matyjek et al. 2020 [25] | IV-V | Yes | HD |  | Whole Body | LTM (kg) | BIA | 40 | 48 | 18 | 20 | 49 | 16 | Yes |
| Matyjek et al. 2020 [25] | IV | Yes | None |  | Whole Body | LTI (%) | BIA | 40 | 48 | 18 | 40 | 59 | 15 | Yes |
| Matyjek et al. 2020 [25] | IV-V | Yes | HD |  | Whole Body | LTI (%) | BIA | 40 | 48 | 18 | 20 | 49 | 16 | Yes |
| Matyjek et al. 2020 [25] | IV | Yes | None |  | Whole Body | BCM (kg) | BIA | 40 | 48 | 18 | 40 | 59 | 15 | Yes |
| Matyjek et al. 2020 [25] | IV-V | Yes | HD |  | Whole Body | BCM (kg) | BIA | 40 | 48 | 18 | 20 | 49 | 16 | Yes |
| Medici et al. 2005 [S31] | ESRD | Yes | PD |  | Whole Body | FFM (kg) | DEXA | 77 | 53 | 17 | 20 | 53 | 19 | No |
| CKD= chronic kidney disease; SD= standard deviation; MAMC= mean arm muscle circumference; CSA= cross-sectional area; LTM= lean tissue mass; LTI= lean tissue index; LBM= lean body mass; FFM= fat free mass; BCM= body cell mass; DEXA= dual energy x-ray absorptiometry; HD= hemodialysis; PD= peritoneal dialysis; MHD= maintenance hemodialysis; ESRD= end-stage renal disease; BIA= bioelectrical impedance analysis Pre-HD: measurement taken prior to dialysis; NIR= near infrared spectroscopy; mo= month. | | | | | | | | | | | | | | |
| **Table S1 continued. Characteristics of Clinical Studies** | | | | | | | | | | | | | | |
| **Study** | **Disease Stage** | **Diabetes** | **Dialysis** | **Additional Defining Variable** | **Muscle** | **Measurement** | **Measurement Method** | **Controls**  **(N)** | **Age** | **SD** | **CKD**  **(N)** | **Age** | **SD** | **Significant Atrophy**  **(p< 0.05)** |
| Medici et al. 2005 [S31] | ESRD | Yes | PD |  | Arm | LTM (kg) | DEXA | 77 | 53 | 17 | 20 | 53 | 19 | No |
| Medici et al. 2005 [S31] | ESRD | Yes | PD |  | Leg | LTM (kg) | DEXA | 77 | 53 | 17 | 20 | 53 | 19 | No |
| Molsted et al. 2007 [26] | ESRD | No | HD |  | Vastus Lateralis | Type I CSA (μm^2^) | Biopsy | 12 | 48 | 12 | 14 | 46 | 18 | Yes |
| Molsted et al. 2007 [26] | ESRD | No | HD |  | Vastus Lateralis | Type II CSA (μm^2^) | Biopsy | 12 | 48 | 12 | 24 | 46 | 18 | No |
| Moraes et al. 2013 [27] | ESRD | Yes | HD |  | Whole Body | FFM (kg) | Anthropometry | 18 | 51 | 7 | 26 | 45 | 14 | No |
| Moraes et al. 2013 [27] | ESRD | Yes | HD |  | Arm | MAMC (cm^2^) | Anthropometry | 18 | 51 | 7 | 26 | 45 | 14 | Yes |
| Morishita et al. 2014 [47] | ESRD | Yes | HD | Male | Whole Body | LTI (kg/m^2^) | BIA | 16 | 37 | 9 | 32 | 67 | 10 | Yes |
| Morishita et al. 2014 [47] | ESRD | Yes | HD | Female | Whole Body | LTI (kg/m^2^) | BIA | 16 | 37 | 9 | 32 | 67 | 10 | Yes |
| Navaneethan et al. 2014 [48] | I-IV | Yes | None |  | Whole Body | LBM (kg) | DEXA | 9433 | 44 | 29 | 2153 | 61 | 32 | Yes |
| Nishizawa et al. 1998 [S32] | ESRD | Yes | HD | Male | Whole Body | LBMI (kg/m^2^) | DEXA | 53 | 49 | 7 | 39 | 57 | 15 | Yes |
| Nishizawa et al. 1998 [S32] | ESRD | Yes | HD | Female | Whole Body | LBMI (kg/m^2^) | DEXA | 114 | 54 | 10 | 64 | 52 | 9 | Yes |
| O'Sullivan et al. 2002 [28] | ESRD | No | Pre-HD |  | Whole Body | LBM (kg) | DEXA | 15 | 64 | 12 | 15 | 71 | 12 | Yes |
| Ohkawa et al. 2005 [29] | ESRD | Yes | HD | Male | Abdominals | Muscle Area (cm^2^) | CT | 33 | 57 | 14 | 85 | 59 | 11 | Yes |
| Ohkawa et al. 2005 [29] | ESRD | Yes | HD | Female | Abdominals | Muscle Area (cm^2^) | CT | 37 | 58 | 20 | 49 | 61 | 14 | Yes |
| Qureshi et al. 1994 [64] | Transplant | No | None | 46 days post Rtx | Whole Body | FFM (kg) | Anthropometry | 25 | <70 | NA | 10 | <70 | NA | No |
| Qureshi et al. 1994 [64] | Transplant | No | None | 13 mo post Rtx | Whole Body | FFM (kg) | Anthropometry | 25 | <70 | NA | 10 | <70 | NA | No |
| Qureshi et al. 1994 [64] | Transplant | No | None | 9 years post Rtx | Whole Body | FFM (kg) | Anthropometry | 25 | <70 | NA | 10 | <70 | NA | No |
| Qureshi et al. 1994 [64] | Transplant | No | None | 46 days post Rtx | Arm | MAMC (cm) | Anthropometry | 25 | <70 | NA | 10 | <70 | NA | No |
| Qureshi et al. 1994 [64] | Transplant | No | None | 13 mo post Rtx | Arm | MAMC (cm) | Anthropometry | 25 | <70 | NA | 10 | <70 | NA | No |
| Qureshi et al. 1994 [64] | Transplant | No | None | 9 years post Rtx | Arm | MAMC (cm) | Anthropometry | 25 | <70 | NA | 10 | <70 | NA | No |
| Rayner et al. 1991 [30] | ESRD | Yes | HD | Male | Arm | MAMC (cm) | Anthropometry | 30 | 42 | 12 | 41 | 48 | 15 | Yes |
| Rayner et al. 1991 [30] | ESRD | Yes | HD | Female | Arm | MAMC (cm) | Anthropometry | 33 | 43 | 10 | 21 | 52 | 14 | No |
| Rodrigues et al. 2016 [49] | III-V | Yes | None |  | Whole Body | LBM (kg) | BIA | 90 | 64 | 10 | 93 | 69 | 12 | No |
| Rodrigues et al. 2016 [49] | III-V | Yes | None |  | Whole Body | LBM (%) | BIA | 90 | 64 | 10 | 93 | 69 | 12 | Yes |
| Rodrigues et al. 2016 [49] | III-V | Yes | None |  | Whole Body | BCM (%) | BIA | 90 | 64 | 10 | 93 | 69 | 12 | No |
| Rodrigues et al. 2016 [49] | III-V | Yes | None |  | Arm | MAMC (%) | Anthropometry | 90 | 64 | 10 | 93 | 69 | 12 | Yes |
| Rymarz et al. 2016 [50] | ESRD | Yes | HD |  | Whole Body | BCM/Weight (kg) | BIA | 33 | 59 | 17 | 48 | 60 | 16 | Yes |
| Rymarz et al. 2016 [50] | ESRD | Yes | HD |  | Whole Body | LTM/Weight (kg) | BIA | 33 | 59 | 17 | 48 | 60 | 16 | No |
| CKD= chronic kidney disease; SD= standard deviation; MAMC= mean arm muscle circumference; CSA= cross-sectional area; LTM= lean tissue mass; LTI= lean tissue index; LBM= lean body mass; LBMI= lean body mass index; FFM= fat free mass; BCM= body cell mass; DEXA= dual energy x-ray absorptiometry; HD= hemodialysis; PD= peritoneal dialysis; ESRD= end-stage renal disease; CT= computed tomography; BIA= bioelectrical impedance analysis. Pre-HD: measurement taken prior to dialysis. | | | | | | | | | | | | | | |
| **Table S1 continued. Characteristics of Clinical Studies** | | | | | | | | | | | | | | |
| **Study** | **Disease Stage** | **Diabetes** | **Dialysis** | **Additional Defining Variable** | **Muscle** | **Measurement** | **Measurement Method** | **Controls**  **(N)** | **Age** | **SD** | **CKD**  **(N)** | **Age** | **SD** | **Significant Atrophy**  **(p< 0.05)** |
| Rymarz et al. 2016 [50] | IV-V | Yes | None |  | Whole Body | BCM/Weight (kg) | BIA | 33 | 59 | 17 | 61 | 60 | 18 | No |
| Rymarz et al. 2016 [50] | IV-V | Yes | None |  | Whole Body | LTM/Weight (kg) | BIA | 33 | 59 | 17 | 61 | 60 | 18 | No |
| Rymarz et al. 2019 [51] | ESRD | Yes | HD |  | Whole Body | LTI (kg/m^2^) | BIA | 15 | 58 | 19 | 41 | 61 | 17 | Yes |
| Rymarz et al. 2019 [51] | ESRD | Yes | HD |  | Whole Body | BCM/Weight (kg) | BIA | 15 | 58 | 19 | 41 | 61 | 17 | Yes |
| Sabatino et al. 2019 [52] | ESRD | Yes | HD | QRFT r 1/2 | Quadriceps | Muscle Thickness (cm) | US | 35 | 41 | 10 | 121 | 67 | 16 | Yes |
| Sabatino et al. 2019 [52] | ESRD | Yes | HD | QVIT r 1/2 | Vastus Lateralis | Muscle Thickness (cm) | US | 35 | 41 | 10 | 121 | 67 | 16 | Yes |
| Sabatino et al. 2019 [52] | ESRD | Yes | HD | QRFT r 2/3 | Quadriceps | Muscle Thickness (cm) | US | 35 | 41 | 10 | 121 | 67 | 16 | Yes |
| Sabatino et al. 2019 [52] | ESRD | Yes | HD | QVIT r 2/3 | Vastus Lateralis | Muscle Thickness (cm) | US | 35 | 41 | 10 | 121 | 67 | 16 | Yes |
| Sakkas et al. 2003 [53] | ESRD | Yes | None |  | Abdominals | Type 1 CSA (μm^2^) | Biopsy | 20 | 59 | 17 | 22 | 55 | 14 | No |
| Sakkas et al. 2003 [53] | ESRD | Yes | None |  | Abdominals | Type II A CSA (μm^2^) | Biopsy | 20 | 59 | 17 | 22 | 55 | 14 | Yes |
| Sakkas et al. 2003 [53] | ESRD | Yes | None |  | Abdominals | Type IIx CSA (μm^2^) | Biopsy | 20 | 59 | 17 | 22 | 55 | 14 | Yes |
| Sakkas et al. 2003 [53] | ESRD | Yes | None |  | Abdominals | Mean CSA (μm^2^) | Biopsy | 20 | 59 | 17 | 22 | 55 | 14 | Yes |
| Segura-Orti et al. 2018 [54] | III-V | Yes | None |  | Quadriceps | Mean CSA (cm^2^) | MRI | 19 | 53 | 8 | 22 | 63 | 10 | Yes |
| Segura-Orti et al. 2018 [54] | ESRD | Yes | MHD |  | Quadriceps | Mean CSA (cm^2^) | MRI | 19 | 53 | 8 | 16 | 55 | 7 | Yes |
| Skouroliakou et al. 2009 [55] | ESRD | Yes | HD |  | Whole Body | Muscle Mass (kg) | BIA | 23 | 49 | 16 | 25 | 53 | 17 | Yes |
| Skouroliakou et al. 2009 [55] | ESRD | Yes | HD |  | Whole Body | FFM (kg) | BIA | 23 | 49 | 16 | 25 | 53 | 17 | No |
| Suneja et al. 2011 [31] | ESRD | No | HD |  | Whole Body | LBM (%) | DEXA | 8 | 48 | 17 | 10 | 41 | 16 | No |
| Tan et al. 2019 [32] | ESRD | Yes | MHD |  | Whole Body | FFM (kg) | BIA | 173 | 56 | 11 | 173 | 58 | 12 | Yes |
| Tan et al. 2019 [32] | ESRD | Yes | MHD |  | Whole Body | BCM/Weight (kg) | BIA | 173 | 56 | 11 | 173 | 58 | 12 | Yes |
| Tan et al. 2019 [32] | ESRD | Yes | MHD |  | Arm | MAMC (cm) | Anthropometry | 173 | 56 | 11 | 173 | 58 | 12 | Yes |
| Teo et al. 2014 [56] | ESRD | Yes | None |  | Arm | MAMC (cm) | Anthropometry | 103 | 43 | 14 | 232 | 58 | 13 | No |
| Van de ham et al. 2005 [S33] | ESRD | No | HD |  | Whole Body | LBM (kg) | DEXA | 21 | 55 | 11 | 16 | 49 | 12 | No |
| Van de ham et al. 2005 [S33] | Transplant | No | None | 6 mo post Rtx | Whole Body | LBM (kg) | DEXA | 21 | 54.9 | 10.8 | 35 | 52.3 | 10.4 | No |
| Vodicar et al. 2018 [33] | ESRD | Yes | HD | 15-33 kg LBM | Whole Body | LBM (kg) | BIA | 106 | 56 | 15 | 90 | 55 | 16 | No |
| Vodicar et al. 2018 [33] | ESRD | Yes | HD | 34-44 kg LBM | Whole Body | LBM (kg) | BIA | 106 | 56 | 15 | 90 | 55 | 16 | No |
| Vodicar et al. 2018 [33] | ESRD | Yes | HD | 45-60 kg LBM | Whole Body | LBM (kg) | BIA | 106 | 56 | 15 | 90 | 55 | 16 | No |
| Wallin et al. 2018 [57] | II-III | Yes | None |  | Whole Body | LBM (kg) | DEXA | 54 | 48 | 11 | 52 | 47 | 11 | No |
| Wallin et al. 2018 [57] | IV-V | Yes | None |  | Whole Body | LBM (kg) | DEXA | 54 | 48 | 11 | 47 | 49 | 12 | No |
| CKD= chronic kidney disease; SD= standard deviation; MAMC= mean arm muscle circumference; CSA= cross-sectional area; LTI= lean tissue index; LBM= lean body mass; FFM= fat free mass; BCM= body cell mass; DEXA= dual energy x-ray absorptiometry; HD= hemodialysis; PD= peritoneal dialysis; MHD= maintenance hemodialysis; ESRD= end-stage renal disease; MRI= magnetic resonance imaging; US= ultrasound; BIA= bioelectrical impedance analysis; QRFT= quadriceps rectus femoris thickness; QVIT= quadriceps vastus intermedius thickness. | | | | | | | | | | | | | | |
| **Table S1 continued. Characteristics of Clinical Studies** | | | | | | | | | | | | | | |
| **Study** | **Disease Stage** | **Diabetes** | **Dialysis** | **Additional Defining Variable** | **Muscle** | **Measurement** | **Measurement Method** | **Controls**  **(N)** | **Age** | **SD** | **CKD**  **(N)** | **Age** | **SD** | **Significant Atrophy**  **(p< 0.05)** |
| Wallin et al. 2020 [34] | II-III | No | None |  | Whole Body | LBM (kg) | DEXA | 54 | 48 | 11 | 52 | 47 | 11 | No |
| Wilkinson et al. 2021 [58] | III-IV | Yes | None | Male | Appendicular | Muscle Mass (kg) | BIA | 195570 | 56 | 8 | 4055 | 63 | 6 | No |
| Wilkinson et al. 2021 [58] | III-IV | Yes | None | Male | Appendicular | Muscle Mass (kg)/m^2^ | BIA | 195570 | 56 | 8 | 4055 | 63 | 6 | Yes |
| Wilkinson et al. 2021 [58] | III-IV | Yes | None | Male | Appendicular | Muscle Mass (kg)/BMI | BIA | 195570 | 56 | 8 | 4055 | 63 | 6 | Yes |
| Wilkinson et al. 2021 [58] | III-IV | Yes | None | Female | Appendicular | Muscle Mass (kg) | BIA | 223983 | 56 | 8 | 4712 | 63 | 6 | Yes |
| Wilkinson et al. 2021 [58] | III-IV | Yes | None | Female | Appendicular | Muscle Mass (kg)/m^2^ | BIA | 223983 | 56 | 8 | 4712 | 63 | 6 | Yes |
| Wilkinson et al. 2021 [58] | III-IV | Yes | None | Female | Appendicular | Muscle Mass (kg)/BMI | BIA | 223983 | 56 | 8 | 4712 | 63 | 6 | Yes |
| Wong et al. 2004 [65] | Transplant | No | None | Men | Whole Body | FFM (%) | BIA | 156 | 35.3 | 10.5 | 55 | 41.8 | 11.4 | No |
| Wong et al. 2004 [65] | Transplant | No | None | Female | Whole Body | FFM (%) | BIA | 263 | 38.6 | 11.7 | 55 | 41.6 | 11.1 | No |
| Woodrow et al. 1996 [59] | II-V | No | None | Male | Whole Body | LBM/kg | DEXA | 17 | 59 | 9 | 12 | 57 | 12 | No |
| Woodrow et al. 1996 [59] | ESRD | No | PD | Male | Whole Body | LBM/kg | DEXA | 17 | 59 | 9 | 12 | 62 | 18 | No |
| Woodrow et al. 1996 [59] | ESRD | No | HD | Male | Whole Body | LBM/kg | DEXA | 17 | 59 | 9 | 11 | 62 | 10 | Yes |
| Woodrow et al. 1996 [59] | II-V | No | None | Female | Whole Body | LBM/kg | DEXA | 16 | 57 | 9 | 11 | 52 | 17 | No |
| Woodrow et al. 1996 [59] | ESRD | No | PD | Female | Whole Body | LBM/kg | DEXA | 16 | 57 | 9 | 12 | 57 | 13 | Yes |
| Woodrow et al. 1996 [59] | ESRD | No | HD | Female | Whole Body | LBM/kg | DEXA | 16 | 57 | 9 | 11 | 61 | 12 | Yes |
| Woodrow et al. 1996 [59] | II-V | No | None | Male | Arm + Leg | LTM (kg) | DEXA | 17 | 59 | 9 | 12 | 57 | 12 | No |
| Woodrow et al. 1996 [59] | ESRD | No | PD | Male | Arm + Leg | LTM (kg) | DEXA | 17 | 59 | 9 | 12 | 62 | 18 | No |
| Woodrow et al. 1996 [59] | ESRD | No | HD | Male | Arm + Leg | LTM (kg) | DEXA | 17 | 59 | 9 | 11 | 62 | 10 | Yes |
| Woodrow et al. 1996 [59] | II-V | No | None | Female | Arm + Leg | LTM (kg) | DEXA | 16 | 57 | 9 | 11 | 52 | 17 | No |
| Woodrow et al. 1996 [59] | ESRD | No | PD | Female | Arm + Leg | LTM (kg) | DEXA | 16 | 57 | 9 | 12 | 57 | 13 | Yes |
| Woodrow et al. 1996 [59] | ESRD | No | HD | Female | Arm + Leg | LTM (kg) | DEXA | 16 | 57 | 9 | 11 | 61 | 12 | Yes |
| Woodrow et al. 1996 [59] | II-V | No | None | Male | Arm | LTM (kg) | DEXA | 17 | 59 | 9 | 12 | 57 | 12 | No |
| Woodrow et al. 1996 [59] | ESRD | No | PD | Male | Arm | LTM (kg) | DEXA | 17 | 59 | 9 | 12 | 62 | 18 | Yes |
| Woodrow et al. 1996 [59] | ESRD | No | HD | Male | Arm | LTM (kg) | DEXA | 17 | 59 | 9 | 11 | 62 | 10 | Yes |
| Woodrow et al. 1996 [59] | II-V | No | None | Female | Arm | LTM (kg) | DEXA | 16 | 57 | 9 | 11 | 52 | 17 | Yes |
| Woodrow et al. 1996 [59] | ESRD | No | PD | Female | Arm | LTM (kg) | DEXA | 16 | 57 | 9 | 12 | 57 | 13 | Yes |
| Woodrow et al. 1996 [59] | ESRD | No | HD | Female | Arm | LTM (kg) | DEXA | 16 | 57 | 9 | 11 | 61 | 12 | Yes |
| Woodrow et al. 1996 [59] | II-V | No | None | Male | Whole Body | FFM (kg) | BIA | 17 | 59 | 9 | 12 | 57 | 13 | No |
| Woodrow et al. 1996 [59] | ESRD | No | PD | Male | Whole Body | FFM (kg) | BIA | 17 | 59 | 9 | 12 | 62 | 18 | No |
| Woodrow et al. 1996 [59] | ESRD | No | HD | Male | Whole Body | FFM (kg) | BIA | 17 | 59 | 9 | 11 | 62 | 10 | Yes |
| CKD= chronic kidney disease; SD= standard deviation; LBM= lean body mass; LTM= lean tissue mass; BMI= body mass index; FFM= fat free mass; DEXA= dual energy x-ray absorptiometry; HD= hemodialysis; PD= peritoneal dialysis; ESRD= end-stage renal disease; BIA= bioelectrical impedance analysis. | | | | | | | | | | | | | | |
| **Table S1 continued. Characteristics of Clinical Studies** | | | | | | | | | | | | | | |
| **Study** | **Disease Stage** | **Diabetes** | **Dialysis** | **Additional Defining Variable** | **Muscle** | **Measurement** | **Measurement Method** | **Controls**  **(N)** | **Age** | **SD** | **CKD**  **(N)** | **Age** | **SD** | **Significant Atrophy**  **(p< 0.05)** |
| Woodrow et al. 1996 [59] | II-V | No | None | Female | Whole Body | FFM (kg) | BIA | 16 | 57 | 9 | 11 | 52 | 17 | No |
| Woodrow et al. 1996 [59] | ESRD | No | PD | Female | Whole Body | FFM (kg) | BIA | 16 | 57 | 9 | 12 | 57 | 13 | Yes |
| Woodrow et al. 1996 [59] | ESRD | No | HD | Female | Whole Body | FFM (kg) | BIA | 16 | 57 | 9 | 11 | 61 | 12 | Yes |
| Woodrow et al. 1996 [59] | II-V | No | None | Male | Arm | MAMC (cm) | Anthropometry | 17 | 59 | 9 | 12 | 57 | 13 | No |
| Woodrow et al. 1996 [59] | ESRD | No | PD | Male | Arm | MAMC (cm) | Anthropometry | 17 | 59 | 9 | 12 | 62 | 18 | No |
| Woodrow et al. 1996 [59] | ESRD | No | HD | Male | Arm | MAMC (cm) | Anthropometry | 17 | 59 | 9 | 11 | 62 | 10 | No |
| Woodrow et al. 1996 [59] | II-V | No | None | Female | Arm | MAMC (cm) | Anthropometry | 16 | 57 | 9 | 11 | 52 | 17 | No |
| Woodrow et al. 1996 [59] | ESRD | No | PD | Female | Arm | MAMC (cm) | Anthropometry | 16 | 57 | 9 | 12 | 57 | 13 | No |
| Woodrow et al. 1996 [59] | ESRD | No | HD | Female | Arm | MAMC (cm) | Anthropometry | 16 | 57 | 9 | 11 | 61 | 12 | No |
| Yilmaz et al. 2020 [60] | ESRD | Yes | None |  | Whole Body | LBM (kg) | BIA | 40 | 46 | 11 | 70 | 49 | 16 | No |
| Yilmaz et al. 2020 [60] | ESRD | Yes | None |  | Whole Body | LBM (kg/m^2^) | BIA | 40 | 46 | 11 | 70 | 49 | 16 | No |
| Young et al. 1997 [S34] | II-V | No | None |  | Whole Body | LBM (kg) | DEXA | 24 | 60 | 8 | 23 | 55 | 15 | No |
| Young et al. 1997 [S34] | ESRD | No | PD |  | Whole Body | LBM (kg) | DEXA | 24 | 60 | 8 | 24 | 59 | 15 | No |
| Young et al. 1997 [S34] | ESRD | No | HD |  | Whole Body | LBM (kg) | DEXA | 24 | 60 | 8 | 22 | 61 | 11 | No |
| Young et al. 1997 [S34] | II-V | No | None |  | Arm | LTM (kg) | DEXA | 24 | 60 | 8 | 23 | 55 | 15 | No |
| Young et al. 1997 [S34] | ESRD | No | PD |  | Arm | LTM (kg) | DEXA | 24 | 60 | 8 | 24 | 59 | 15 | No |
| Young et al. 1997 [S34] | ESRD | No | HD |  | Arm | LTM (kg) | DEXA | 24 | 60 | 8 | 22 | 61 | 11 | Yes |
| Young et al. 1997 [S34] | II-V | No | None |  | Leg | LTM (kg) | DEXA | 24 | 60 | 8 | 23 | 55 | 15 | No |
| Young et al. 1997 [S34] | ESRD | No | PD |  | Leg | LTM (kg) | DEXA | 24 | 60 | 8 | 24 | 59 | 15 | No |
| Young et al. 1997 [S34] | ESRD | No | HD |  | Leg | LTM (kg) | DEXA | 24 | 60 | 8 | 22 | 61 | 11 | Yes |
| Zamojska et al. 2006 [S35] | ESRD | Yes | HD |  | Arm | MAMC (cm) | Anthropometry | 16 | 56 | 6 | 60 | 60 | 13 | No |
| Zamojska et al. 2006 [S35] | ESRD | Yes | HD |  | Whole Body | LBM (kg) | BIA | 16 | 56 | 6 | 60 | 60 | 13 | No |
| Zamojska et al. 2006 [S35] | ESRD | Yes | HD |  | Whole Body | BCM/Weight (kg) | BIA | 16 | 56 | 6 | 60 | 60 | 13 | No |
| CKD= chronic kidney disease; SD= standard deviation; MAMC= mean arm muscle circumference; LBM= lean body mass; LTM= lean tissue mass; FFM= fat free mass; BCM= body cell mass; DEXA= dual energy x-ray absorptiometry; HD= hemodialysis; PD= peritoneal dialysis; ESRD= end-stage renal disease; BIA= bioelectrical impedance analysis. | | | | | | | | | | | | | | |

| **Table S2. Characteristics of Preclinical Studies** | | | | | | | | | | | |
| --- | --- | --- | --- | --- | --- | --- | --- | --- | --- | --- | --- |
| **Study** | **Species** | **Disease Model** | **Muscle** | **Measurement** | **Measurement Method** | **Controls**  **(N)** | **Age** | **CKD**  **(N)** | **Age** | **Significant Atrophy**  **(p< 0.05)** |  |
| Acevedo et al. 2016 [66] | Rat | 5/6 | Soleus | Weight (mg) | Terminal Procurement | 12 | 30 weeks | 12 | 30 weeks | Yes |  |
| Acevedo et al. 2016 [66] | Rat | 5/6 | Tibialis Cranialis | Weight (mg) | Terminal Procurement | 12 | 30 weeks | 12 | 30 weeks | No |  |
| Acevedo et al. 2016 [66] | Rat | 5/6 | Soleus | Type I CSA (μm^2^) | Terminal Procurement | 12 | 30 weeks | 12 | 30 weeks | No |  |
| Acevedo et al. 2016 [66] | Rat | 5/6 | Tibialis Cranialis | Type IIa CSA (μm^2^) | Terminal Procurement | 12 | 30 weeks | 12 | 30 weeks | No |  |
| Acevedo et al. 2016 [66] | Rat | 5/6 | Tibialis Cranialis | Type IIx CSA (μm^2^) | Terminal Procurement | 12 | 30 weeks | 12 | 30 weeks | Yes |  |
| Acevedo et al. 2016 [66] | Rat | 5/6 | Tibialis Cranialis | Type IIb CSA (μm^2^) | Terminal Procurement | 12 | 30 weeks | 12 | 30 weeks | No |  |
| Adams et al. 2005 [67] | Rat | 5/6 | Gastrocnemius | Weight (mg) | Terminal Procurement | 12 | Not Reported | 12 | Not Reported | No |  |
| Amann et al. 1997 [68] | Rat | 5/6 | Psoas | Mean CSA (μm^2^) | Terminal Procurement | 9 | 16 weeks | 9 | 16 weeks | No |  |
| Andres-Hernando et al. 2019 [69] | Mouse | Adenine | Tibialis Anterior | Weight (mg) | Terminal Procurement | 5 | 52 weeks | 5 | 52 weeks | No |  |
| Andres-Hernando et al. 2019 [69] | Mouse | Adenine | Gastrocnemius | Weight (mg) | Terminal Procurement | 5 | 52 weeks | 5 | 52 weeks | No |  |
| Andres-Hernando et al. 2019 [69] | Mouse | Adenine | Soleus | Weight (mg) | Terminal Procurement | 5 | 52 weeks | 5 | 52 weeks | No |  |
| Berru et al. 2019 [70] | Mouse | Adenine | Soleus | Mean CSA (μm^2^) | Terminal Procurement | 8 | Not Reported | 8 | Not Reported | No |  |
| Cheung et al. 2020 [71] | Mouse | 5/6 | Tibilais Anterior | Mean CSA (μm^2^) | Terminal Procurement | 11 | 12 weeks | 11 | 12 weeks | Yes |  |
| Cheung et al. 2020 [71] | Mouse | 5/6 | Soleus | Mean CSA (μm^2^) | Terminal Procurement | 11 | 12 weeks | 11 | 12 weeks | Yes |  |
| Cheung et al. 2021 [101] | Mouse | 5/6 | Gastrocnemius | Mean CSA (μm^2^) | Terminal Procurement | 6 | 14 weeks | 6 | 14 weeks | Yes |  |
| De Moraes et al. 2018 [72] | Rat | 5/6 | Tibialis Anterior | Mean CSA (μm^2^) | Terminal Procurement | 16 | Not Reported | 16 | Not Reported | Yes |  |
| De Moraes et al. 2018 [72] | Rat | 5/6 | Tibialis Anterior | Weight (mg) | Terminal Procurement | 16 | Not Reported | 16 | Not Reported | Yes |  |
| Enoki et al. 2017 [73] | Mouse | 5/6 | Tibialis Anterior | Weight (mg) | Terminal Procurement | 7 | 34 weeks | 7 | 34 weeks | No |  |
| Enoki et al. 2017 [73] | Mouse | 5/6 | Soleus | Weight (mg) | Terminal Procurement | 7 | 34 weeks | 7 | 34 weeks | Yes |  |
| Enoki et al. 2017 [73] | Mouse | 5/6 | Gastrocnemius | Weight (mg) | Terminal Procurement | 7 | 34 weeks | 7 | 34 weeks | Yes |  |
| Flisinski et al. 2014 [74] | Rat | 1/2 | Gastrocnemius | Type I CSA (μm) | Terminal Procurement | 12 | Not Reported | 12 | Not Reported | No |  |
| Flisinski et al. 2014 [74] | Rat | 1/2 | Gastrocnemius | Type IIa CSA (μm) | Terminal Procurement | 12 | Not Reported | 12 | Not Reported | No |  |
| Flisinski et al. 2014 [74] | Rat | 1/2 | Gastrocnemius | Type IIx CSA (μm) | Terminal Procurement | 12 | Not Reported | 12 | Not Reported | No |  |
| Flisinski et al. 2014 [74] | Rat | 1/2 | Gastrocnemius | Type IIb CSA (μm) | Terminal Procurement | 12 | Not Reported | 12 | Not Reported | No |  |
| Flisinski et al. 2014 [74] | Rat | 1/2 | Longissimus Thoracis | Type I CSA (μm) | Terminal Procurement | 12 | Not Reported | 12 | Not Reported | No |  |
| Flisinski et al. 2014 [74] | Rat | 1/2 | Longissimus Thoracis | Type IIa CSA (μm) | Terminal Procurement | 12 | Not Reported | 12 | Not Reported | No |  |
| Flisinski et al. 2014 [74] | Rat | 1/2 | Longissimus Thoracis | Type IIx CSA (μm) | Terminal Procurement | 12 | Not Reported | 12 | Not Reported | No |  |
| Flisinski et al. 2014 [74] | Rat | 1/2 | Longissimus Thoracis | Type IIb CSA (μm) | Terminal Procurement | 12 | Not Reported | 12 | Not Reported | No |  |
| CKD= chronic kidney disease; CSA= cross sectional area; 5/6= 5/6 nephrectomy; 1/2= 1/2 nephrectomy. | | | | | | | | | | | |
| **Table S2 continued. Characteristics of Preclinical Studies** | | | | | | | | | | | |
| **Study** | **Species** | **Disease Model** | **Muscle** | **Measurement** | **Measurement Method** | **Controls**  **(N)** | **Age** | **CKD**  **(N)** | **Age** | **Significant Atrophy**  **(p< 0.05)** |  |
| Flisinski et al. 2014 [74] | Rat | 5/6 | Gastrocnemius | Type I CSA (μm) | Terminal Procurement | 12 | Not Reported | 12 | Not Reported | No |  |
| Flisinski et al. 2014 [74] | Rat | 5/6 | Gastrocnemius | Type IIa CSA (μm) | Terminal Procurement | 12 | Not Reported | 12 | Not Reported | No |  |
| Flisinski et al. 2014 [74] | Rat | 5/6 | Gastrocnemius | Type IIx CSA (μm) | Terminal Procurement | 12 | Not Reported | 12 | Not Reported | No |  |
| Flisinski et al. 2014 [74] | Rat | 5/6 | Gastrocnemius | Type IIb CSA (μm) | Terminal Procurement | 12 | Not Reported | 12 | Not Reported | No |  |
| Flisinski et al. 2014 [74] | Rat | 5/6 | Longissimus Thoracis | Type I CSA (μm) | Terminal Procurement | 12 | Not Reported | 12 | Not Reported | No |  |
| Flisinski et al. 2014 [74] | Rat | 5/6 | Longissimus Thoracis | Type IIa CSA (μm) | Terminal Procurement | 12 | Not Reported | 12 | Not Reported | No |  |
| Flisinski et al. 2014 [74] | Rat | 5/6 | Longissimus Thoracis | Type IIx CSA (μm) | Terminal Procurement | 12 | Not Reported | 12 | Not Reported | No |  |
| Flisinski et al. 2014 [74] | Rat | 5/6 | Longissimus Thoracis | Type IIb CSA (μm) | Terminal Procurement | 12 | Not Reported | 12 | Not Reported | No |  |
| Hu et al. 2019 [75] | Mouse | 5/6 | Tibialis Anterior | Mean CSA (μm) | Terminal Procurement | 6 | Not Reported | 6 | Not Reported | Yes |  |
| Hu et al. 2010 [76] | Mouse | 5/6 | Extensor Digitorum Longus | Weight (mg) | Terminal Procurement | 9 | Not Reported | 9 | Not Reported | Yes |  |
| Hu et al. 2010 [76] | Mouse | 5/6 | Soleus | Weight (mg) | Terminal Procurement | 9 | Not Reported | 9 | Not Reported | Yes |  |
| Hu et al. 2010 [76] | Mouse | 5/6 | Plantaris | Weight (mg) | Terminal Procurement | 9 | Not Reported | 9 | Not Reported | Yes |  |
| Hu et al. 2010 [76] | Mouse | 5/6 | Tibialis Anterior | Mean CSA | Terminal Procurement | 9 | Not Reported | 9 | Not Reported | Yes |  |
| Hu et al. 2015 [100] | Mouse | 5/6 | Extensor Digitorum Longus | Weight (mg) | Terminal Procurement | 12 | Not Reported | 12 | Not Reported | Yes |  |
| Hu et al. 2015 [100] | Mouse | 5/6 | Soleus | Weight (mg) | Terminal Procurement | 12 | Not Reported | 12 | Not Reported | Yes |  |
| Kim et al. 2021 [13] | Mouse | Adenine | Extensor Digitorum Longus | Muscle CSA (μm^2^) | Terminal Procurement | 5 | 22 weeks | 5 | 22 weeks | Yes |  |
| Kim et al. 2021 [13] | Mouse | Adenine | Extensor Digitorum Longus | Muscle CSA (μm^2^) | Terminal Procurement | 5 | 22 weeks | 5 | 22 weeks | No |  |
| Kim et al. 2021 [13] | Mouse | Adenine | Soleus | Muscle CSA (μm^2^) | Terminal Procurement | 5 | 22 weeks | 5 | 22 weeks | Yes |  |
| Kim et al. 2021 [13] | Mouse | Adenine | Soleus | Muscle CSA (μm^2^) | Terminal Procurement | 5 | 22 weeks | 5 | 22 weeks | No |  |
| Kim et al. 2021 [13] | Mouse | Adenine | Extensor Digitorum Longus | Weight (mg) | Terminal Procurement | 5 | 22 weeks | 5 | 22 weeks | Yes |  |
| Kim et al. 2021 [13] | Mouse | Adenine | Extensor Digitorum Longus | Weight (mg) | Terminal Procurement | 5 | 22 weeks | 5 | 22 weeks | No |  |
| Kim et al. 2021 [13] | Mouse | Adenine | Soleus | Weight (mg) | Terminal Procurement | 5 | 22 weeks | 5 | 22 weeks | Yes |  |
| Kim et al. 2021 [13] | Mouse | Adenine | Soleus | Weight (mg) | Terminal Procurement | 5 | 22 weeks | 5 | 22 weeks | No |  |
| Kim et al. 2021 [13] | Mouse | 5/6 | Extensor Digitorum Longus | Muscle CSA (μm^2^) | Terminal Procurement | 5 | 16 weeks | 5 | 16 weeks | Yes |  |
| Kim et al. 2021 [13] | Mouse | 5/6 | Extensor Digitorum Longus | Muscle CSA (μm^2^) | Terminal Procurement | 5 | 16 weeks | 5 | 16 weeks | No |  |
| Kim et al. 2021 [13] | Mouse | 5/6 | Soleus | Muscle CSA (μm^2^) | Terminal Procurement | 5 | 16 weeks | 5 | 16 weeks | Yes |  |
| Kim et al. 2021 [13] | Mouse | 5/6 | Soleus | Muscle CSA (μm^2^) | Terminal Procurement | 5 | 16 weeks | 5 | 16 weeks | No |  |
| Kim et al. 2021 [13] | Mouse | 5/6 | Extensor Digitorum Longus | Weight (mg) | Terminal Procurement | 5 | 16 weeks | 5 | 16 weeks | Yes |  |
| Kim et al. 2021 [13] | Mouse | 5/6 | Extensor Digitorum Longus | Weight (mg) | Terminal Procurement | 5 | 16 weeks | 5 | 16 weeks | No |  |
| Kim et al. 2021 [13] | Mouse | 5/6 | Soleus | Weight (mg) | Terminal Procurement | 5 | 16 weeks | 5 | 16 weeks | Yes |  |
| CKD= chronic kidney disease; CSA= cross sectional area; 5/6= 5/6 nephrectomy. | | | | | | | | | | | |
| **Table S2 continued. Characteristics of Preclinical Studies** | | | | | | | | | | | |
| **Study** | **Species** | **Disease Model** | **Muscle** | **Measurement** | **Measurement Method** | **Controls**  **(N)** | **Age** | **CKD**  **(N)** | **Age** | **Significant Atrophy**  **(p< 0.05)** |  |
| Kim et al. 2021 [13] | Mouse | 5/6 | Soleus | Weight (mg) | Terminal Procurement | 5 | 16 weeks | 5 | 16 weeks | No |  |
| Liu et al. 2021 [77] | rats | 5/6 | Tibialis Anterior | Mean CSA (μm^2^) | Terminal Procurement | 10 | Not Reported | 10 | Not Reported | Yes |  |
| Mitrou et al. 2019 [78] | Rabbit | 3/4 | Psoas | Mean CSA (μm^2^) | Terminal Procurement | 6 | Not Reported | 6 | Not Reported | Yes |  |
| Mitrou et al. 2017 [79] | Rabbit | 3/4 | Psoas | Mean CSA (μm^2^) | Terminal Procurement | 2 | Not Reported | 2 | Not Reported | Yes |  |
| Moraes et al. 2018 [80] | Rat | 5/6 | Soleus | Weight (g) | Terminal Procurement | 10 | 16 weeks | 10 | 16 weeks | Yes |  |
| Moraes et al. 2018 [80] | Rat | 5/6 | Extensor Digitorum Longus | Weight (g) | Terminal Procurement | 10 | 16 weeks | 10 | 16 weeks | Yes |  |
| Moraes et al. 2018 [80] | Rat | 5/6 | Plantaris | Weight (g) | Terminal Procurement | 10 | 16 weeks | 10 | 16 weeks | No |  |
| Niida et al. 2020 [81] | Mouse | Adenine | Gastrocnemius | Weight (g) | Terminal Procurement | 4-7 | 14 weeks | 4-7 | 14 weeks | No |  |
| Niida et al. 2020 [81] | Mouse | Adenine | Gastrocnemius | Mean CSA (μm^2^) | Terminal Procurement | 4-7 | 14 weeks | 4-7 | 14 weeks | Yes |  |
| Niida et al. 2020 [81] | Rat | Adenine | Soleus | Weight (g) | Terminal Procurement | 3-5 | 14 weeks | 3-5 | 14 weeks | No |  |
| Niida et al. 2020 [81] | Rat | Adenine | Gastrocnemius | Weight (g) | Terminal Procurement | 3-5 | 14 weeks | 3-5 | 14 weeks | No |  |
| Niida et al. 2020 [81] | Rat | Adenine | Tibialis Anterior | Weight (g) | Terminal Procurement | 3-5 | 14 weeks | 3-5 | 14 weeks | No |  |
| Niida et al. 2020 [81] | Rat | Adenine | Extensor Digitorum Longus | Weight (g) | Terminal Procurement | 3-5 | 14 weeks | 3-5 | 14 weeks | No |  |
| Niida et al. 2020 [81] | Rat | Adenine | Gastrocnemius | Mean CSA (μm^2^) | Terminal Procurement | 4-7 | 14 weeks | 4-7 | 14 weeks | No |  |
| Organ et al. 2016 [82] | Rat | Cy/+ | Soleus | Weight (g) | Terminal Procurement | 7 | 35 weeks | 7 | 35 weeks | Yes |  |
| Organ et al. 2016 [82] | Rat | Cy/+ | Extensor Digitorum Longus | Weight (g) | Terminal Procurement | 7 | 35 weeks | 7 | 35 weeks | Yes |  |
| Organ et al. 2016 [82] | Rat | Cy/+ | Soleus | Mean CSA (μm^2^) | Terminal Procurement | 7 | 35 weeks | 7 | 35 weeks | Yes |  |
| Organ et al. 2016 [82] | Rat | Cy/+ | Extensor Digitorum Longus | Mean CSA (μm^2^) | Terminal Procurement | 7 | 35 weeks | 7 | 35 weeks | Yes |  |
| Organ et al. 2016 [82] | Rat | Cy/+ | Soleus | Type I CSA (μm^2^) | Terminal Procurement | 7 | 35 weeks | 7 | 35 weeks | Yes |  |
| Organ et al. 2016 [82] | Rat | Cy/+ | Soleus | Type IIa CSA (μm^2^) | Terminal Procurement | 7 | 35 weeks | 7 | 35 weeks | Yes |  |
| Organ et al. 2016 [82] | Rat | Cy/+ | Extensor Digitorum Longus | Type I CSA (μm^2^) | Terminal Procurement | 7 | 35 weeks | 7 | 35 weeks | Yes |  |
| Organ et al. 2016 [82] | Rat | Cy/+ | Extensor Digitorum Longus | Type IIa CSA (μm^2^) | Terminal Procurement | 7 | 35 weeks | 7 | 35 weeks | Yes |  |
| Pan et al. 2019 [83] | Mouse | 5/6 | Posterior Limbs | Weight (g) | Terminal Procurement | 9 | 24 weeks | 9 | 24 weeks | Yes |  |
| Pan et al. 2019 [83] | Mouse | 5/6 | Posterior Limbs | Mean CSA (μm^2^) | Terminal Procurement | 9 | 24 weeks | 9 | 24 weeks | Yes |  |
| Qian et al. 2019 [84] | Mouse | 5/6 | Tibialis Anterior | Weight (mg) | Terminal Procurement | 6 | 20 weeks | 6 | 20 weeks | Yes |  |
| Qian et al. 2019 [84] | Mouse | 5/6 | Gastrocnemius | Weight (mg) | Terminal Procurement | 6 | 20 weeks | 6 | 20 weeks | No |  |
| Qian et al. 2019 [84] | Mouse | 5/6 | Tibialis Anterior | Mean CSA (μm^2^) | Terminal Procurement | 6 | 20 weeks | 6 | 20 weeks | Yes |  |
| Sato et al. 2016 [85] | Mouse | Adenine | Tibialis Anterior | Mean CSA (μm^2^) | Terminal Procurement | 30 | 13 weeks | 30 | 13 weeks | Yes |  |
| Saud et al. 2021 [86] | Rat | 5/6 | Extensor Digitorum Longus | Weight (mg)/tibia length (cm) | Terminal Procurement | 8 | Not Reported | 8 | Not Reported | No |  |
| CKD= chronic kidney disease; CSA= cross sectional area; 5/6= 5/6 nephrectomy; 3/4= 3/4 nephrectomy. | | | | | | | | | | | |
| **Table S2 continued. Characteristics of Preclinical Studies** | | | | | | | | | | | |
| **Study** | **Species** | **Disease Model** | **Muscle** | **Measurement** | **Measurement Method** | **Controls**  **(N)** | **Age** | **CKD**  **(N)** | **Age** | **Significant Atrophy**  **(p< 0.05)** |  |
| Saud et al. 2021 [86] | Rat | 5/6 | Tibialis Anterior | Weight (g)/tibia length (cm) | Terminal Procurement | 8 | Not Reported | 8 | Not Reported | Yes |  |
| Saud et al. 2021 [86] | Rat | 5/6 | Plantar | Weight (g)/tibia length (cm) | Terminal Procurement | 8 | Not Reported | 8 | Not Reported | Yes |  |
| Saud et al. 2021 [86] | Rat | 5/6 | Soleus | Weight (g)/tibia length (cm) | Terminal Procurement | 8 | Not Reported | 8 | Not Reported | Yes |  |
| Saud et al. 2021 [86] | Rat | 5/6 | Extensor Digitorum Longus+Tibial+Plantar+Soleus | Weight (g)/tibia length (cm) | Terminal Procurement | 8 | Not Reported | 8 | Not Reported | Yes |  |
| Sologna et al. 2021 [87] | Mouse | Adenine | Gastrocnemius | Weight (mg) | Terminal Procurement | 5 | 6 weeks | 5 | 6 weeks | Yes |  |
| Souza et al. 2018 [88] | Rat | 5/6 | Soleus+Gastroc+Quadriceps | Weight (g) | Terminal Procurement | 5 | 22 weeks | 5 | 22 weeks | No |  |
| Thome et al. 2020 [89] | Mouse | Adenine | Tibialis Anterior | Weight (mg) | Terminal Procurement | 10-14 | Not Reported | 10-14 | Not Reported | Yes |  |
| Thome et al. 2020 [89] | Mouse | Adenine | Gastrocnemius | Weight (mg) | Terminal Procurement | 10-14 | Not Reported | 10-14 | Not Reported | Yes |  |
| Thome et al. 2020 [89] | Mouse | Adenine | Extensor Digitorum Longus | Weight (mg) | Terminal Procurement | 10-14 | Not Reported | 10-14 | Not Reported | Yes |  |
| Thome et al. 2020 [89] | Mouse | Adenine | Soleus | Weight (mg) | Terminal Procurement | 10-14 | Not Reported | 10-14 | Not Reported | Yes |  |
| Thome et al. 2020 [89] | Mouse | Adenine | Tibialis Anterior | Weight (mg) | Terminal Procurement | 10-14 | Not Reported | 10-14 | Not Reported | No |  |
| Thome et al. 2020 [89] | Mouse | Adenine | Gastrocnemius | Weight (mg) | Terminal Procurement | 10-14 | Not Reported | 10-14 | Not Reported | No |  |
| Thome et al. 2020 [89] | Mouse | Adenine | Extensor Digitorum Longus | Weight (mg) | Terminal Procurement | 10-14 | Not Reported | 10-14 | Not Reported | No |  |
| Thome et al. 2020 [89] | Mouse | Adenine | Soleus | Weight (mg) | Terminal Procurement | 10-14 | Not Reported | 10-14 | Not Reported | Yes |  |
| Thome et al. 2020 [89] | Mouse | Adenine | Soleus | Mean CSA (μm^2^) | Terminal Procurement | 10-14 | Not Reported | 10-14 | Not Reported | No |  |
| Thome et al. 2020 [89] | Mouse | Adenine | Soleus | Mean CSA (μm^2^) | Terminal Procurement | 10-14 | Not Reported | 10-14 | Not Reported | No |  |
| Uchiyama et al. 2020 [90] | Mouse | Adenine | Gastrocnemius | Weight (mg) | Terminal Procurement | 10 | 14 weeks | 10 | 14 weeks | Yes |  |
| Uchiyama et al. 2020 [90] | Mouse | Adenine | Soleus | Weight (mg) | Terminal Procurement | 10 | 14 weeks | 10 | 14 weeks | Yes |  |
| Wang et al. 2018 [91] | Rat | 5/6 | Quadriceps | Weight (g) | Terminal Procurement | 6 | Not Reported | 6 | Not Reported | Yes |  |
| Wang et al. 2018 [91] | Rat | 5/6 | Gastrocnemius | Weight (g) | Terminal Procurement | 6 | Not Reported | 6 | Not Reported | No |  |
| Wang et al. 2018 [91] | Rat | 5/6 | Tibialis Anterior | Weight (g) | Terminal Procurement | 6 | Not Reported | 6 | Not Reported | No |  |
| Wang et al. 2018 [91] | Rat | 5/6 | Extensor Digitorum Longus | Weight (g) | Terminal Procurement | 6 | Not Reported | 6 | Not Reported | Yes |  |
| Wang et al. 2018 [91] | Rat | 5/6 | Tibialis Anterior | Mean CSA (mm^2^) | Terminal Procurement | 6 | Not Reported | 6 | Not Reported | Yes |  |
| Wang et al. 2019 [92] | Rat | 5/6 | Gastrocnemius | Weight (mg) | Terminal Procurement | 10 | 12 weeks | 10 | 12 weeks | Yes |  |
| Wang et al. 2019 [92] | Rat | 5/6 | Tibialis Anterior | Weight (mg) | Terminal Procurement | 10 | 12 weeks | 10 | 12 weeks | Yes |  |
| Wang et al. 2019 [92] | Rat | 5/6 | Soleus | Weight (mg) | Terminal Procurement | 10 | 12 weeks | 10 | 12 weeks | Yes |  |
| Wu et al. 2019 [93] | Mouse | ST | Gastrocnemius | Weight (g) | Terminal Procurement | 4-6 | 22 weeks | 4-6 | 22 weeks | Yes |  |
| Wu et al. 2019 [93] | Mouse | ST | Whole Body | LBM (g) | DEXA | 4-6 | 22 weeks | 4-6 | 22 weeks | Yes |  |
| Xia et al. 2021 [94] | Rat | 5/6 | Tibialis Anterior | Mean CSA (μm^2^) | Terminal Procurement | 8 | 24 weeks | 8 | 24 weeks | Yes |  |
| CKD= chronic kidney disease; CSA= cross sectional area; 5/6= 5/6 nephrectomy; ST = subtotal nephrectomy; LBM= lean body mass. | | | | | | | | | | | |
| **Table S2 continued. Characteristics of Preclinical Studies** | | | | | | | | | | | |
| **Study** | **Species** | **Disease Model** | **Muscle** | **Measurement** | **Measurement Method** | **Controls**  **(N)** | **Age** | **CKD**  **(N)** | **Age** | **Significant Atrophy**  **(p< 0.05)** |  |
| Yoshida et al. 2017 [95] | Rat | 5/6 | Gastrocnemius | Weight (g) | Terminal Procurement | 6 | Not Reported | 6 | Not Reported | No |  |
| Yoshida et al. 2017 [95] | Rat | 5/6 | Soleus | Weight (mg) | Terminal Procurement | 6 | Not Reported | 6 | Not Reported | Yes |  |
| Yoshida et al. 2017 [95] | Rat | 5/6 | Soleus | Mean CSA (μm^2^) | Terminal Procurement | 6 | Not Reported | 6 | Not Reported | Yes |  |
| Zhang et al. 2019 [97] | Mouse | 5/6 | Gastrocnemius | Weight (mg) | Terminal Procurement | 6 | 24 weeks | 6 | 24 weeks | Yes |  |
| Zhang et al. 2019 [97] | Mouse | 5/6 | Tibialis Anterior | Weight (mg) | Terminal Procurement | 6 | 24 weeks | 6 | 24 weeks | Yes |  |
| Zhang et al. 2015 [96] | Rat | 5/6 | Gastrocnemius | Weight (g) | Terminal Procurement | 10 | Not Reported | 10 | Not Reported | Yes |  |
| Zhang et al. 2015 [96] | Rat | 5/6 | Gastrocnemius | Mean CSA (μm^2^) | Terminal Procurement | 10 | Not Reported | 10 | Not Reported | Yes |  |
| Zhang et al. 2020 [98] | Rat | 5/6 | Tibialis anterior | Weight (mg) | Terminal Procurement | 9 | Not Reported | 9 | Not Reported | Yes |  |
| Zhang et al. 2021 [99] | Rat | 5/6 | Gastrocnemius | Mean CSA (%) | Terminal Procurement | 8 | 24 weeks | 8 | 24 weeks | Yes |  |
| Zhang et al. 2021 [99] | Rat | 5/6 | Gastrocnemius | Weight (%) | Terminal Procurement | 8 | 24 weeks | 8 | 24 weeks | Yes |  |
| CKD= chronic kidney disease; CSA= cross sectional area; 5/6= 5/6 nephrectomy. | | | | | | | | | | | |

| **Table S3. Clinical Studies (Excluding Transplant Studies) Quality Analysis via the Newcastle-Ottawa Scale (NOS)** | | | | | | | | | | | | | |
| --- | --- | --- | --- | --- | --- | --- | --- | --- | --- | --- | --- | --- | --- |
|  | **Selection** | | | | | **Comparability** | | | **Ascertainment** | | | | **Overall** |
| **Study** | **1.1** | **1.2** | **1.3** | **1.4** | **Quality** | **2.1** | **2.2** | **Quality** | **3.1** | **3.2** | **3.3** | **Quality** | **Quality** |
| Abramowitz et al. 2018 [35] | Yes | No | No | Yes | Fair | Yes | No | Fair | No | Yes | Yes | Fair | Fair |
| Adey et al. 2000 [14] | No | Yes | No | Yes | Fair | Yes | Yes | Good | No | Yes | Yes | Fair | Fair |
| Akdam et al. 2016 [36] | Yes | No | No | Yes | Fair | Yes | No | Fair | No | Yes | Yes | Fair | Fair |
| Aniort et al. 2021 [15] | No | No | No | Yes | Poor | Yes | Yes | Good | No | Yes | Yes | Fair | Poor |
| Agarwal et al. 2010 [S36] | No | No | Yes | Yes | Fair | Yes | No | Fair | No | Yes | Yes | Fair | Fair |
| Avesani et al. 2004 [37] | Yes | No | Yes | Yes | Good | Yes | Yes | Good | No | Yes | Yes | Fair | Good |
| Axelsson et al. 2006 [S37] | Yes | Yes | No | Yes | Good | Yes | No | Fair | No | Yes | Yes | Fair | Fair |
| Banzanelli et al. 2006 [S16] | Yes | Yes | No | Yes | Good | Yes | No | Fair | No | Yes | Yes | Fair | Fair |
| Baria et al. 2011 [38] | Yes | No | Yes | Yes | Good | Yes | No | Fair | No | Yes | Yes | Fair | Fair |
| Battaglia et al. 2020 [39] | Yes | No | Yes | Yes | Good | Yes | No | Fair | No | Yes | Yes | Fair | Fair |
| Boivin et al. 2010 [S17] | No | No | No | No | Poor | Yes | No | Fair | Yes | No | Yes | Fair | Poor |
| Bucar-Pajek et al. 2016 [16] | Yes | No | No | Yes | Fair | Yes | No | Fair | No | Yes | Yes | Fair | Fair |
| Bueno et al. 2017 [17] | Yes | Yes | No | Yes | Good | Yes | No | Fair | No | Yes | Yes | Fair | Fair |
| Chen et al. 2000 [S18] | No | No | No | No | Poor | Yes | Yes | Good | No | Yes | Yes | Fair | Poor |
| Crowe et al. 2007 [S19] | No | Yes | No | No | Poor | Yes | Yes | Good | Yes | Yes | Yes | Good | Poor |
| Cuptsi et al. 2004 [S20] | Yes | Yes | No | Yes | Good | Yes | Yes | Good | No | Yes | Yes | Fair | Good |
| Diesel et al. 1993 [S21] | Yes | No | Yes | Yes | Good | Yes | No | Fair | No | Yes | Yes | Fair | Fair |
| Elsayed et al. 2009 [S22] | Yes | No | Yes | Yes | Good | Yes | No | Fair | No | Yes | Yes | Fair | Fair |
| Fahal et al. 1997 [40] | Yes | No | No | Yes | Fair | Yes | No | Fair | No | Yes | Yes | Fair | Fair |
| Fisch et al. 1996 [18] | Yes | Yes | No | Yes | Good | Yes | No | Fair | No | Yes | Yes | Fair | Fair |
| Guida et al. 2001 [S23] | Yes | No | No | Yes | Fair | Yes | No | Fair | No | Yes | Yes | Fair | Fair |
| Han et al. 2011 [19] | Yes | Yes | Yes | Yes | Good | Yes | No | Fair | No | Yes | Yes | Fair | Fair |
| Isoyama et al. 2016 [41] | Yes | No | No | Yes | Fair | Yes | No | Fair | No | Yes | Yes | Fair | Fair |
| Jairam et al. 2010 [S24] | Yes | Yes | Yes | Yes | Good | Yes | No | Fair | No | Yes | Yes | Fair | Fair |
| Johansen et al. 2003 [6] | Yes | No | Yes | Yes | Good | Yes | No | Fair | No | Yes | Yes | Fair | Fair |
| Johansen et al. 1998 [S25] | Yes | Yes | Yes | Yes | Good | Yes | No | Fair | No | Yes | Yes | Fair | Fair |
| Kamimura et al. 2007 [S26] | Yes | No | No | Yes | Fair | Yes | Yes | Good | No | Yes | Yes | Fair | Fair |
| Kemp et al. 2004 [S27] | No | No | No | Yes | Poor | Yes | Yes | Good | No | Yes | Yes | Fair | Poor |
| Kim et al. 2020 [20] | Yes | No | Yes | Yes | Good | Yes | Yes | Good | No | Yes | Yes | Fair | Good |
| 1.1= Truly representative of the average adult (>18 years old) chronic kidney disease patient in the community; 1.2= somewhat representative of the average adult (age gap <10 years) chronic kidney disease patient in the community; 1.3= controls are drawn from the same community as the chronic kidney disease patients; 1.4= determined chronic kidney disease via secure record (for example: Medical chart or they measured themselves); 2.1= study controls for muscle size measurement; 2.2= study controls for diabetes or excludes diabetes; 3.1= assessment of outcome performed via blind assessment; 3.2= assessment of outcomes performed via record linkage (took measurement, but did not blind); 3.3= follow-up was long enough for outcomes to occur | | | | | | | | | | | | | |
| **Table S3 continued. Clinical Studies (Excluding Transplant Studies) Quality Analysis via the Newcastle-Ottawa Scale (NOS)** | | | | | | | | | | | | | |
|  | **Selection** | | | | | **Comparability** | | | **Ascertainment** | | | | **Overall** |
| **Study** | **1.1** | **1.2** | **1.3** | **1.4** | **Quality** | **2.1** | **2.2** | **Quality** | **3.1** | **3.2** | **3.3** | **Quality** | **Quality** |
| Kirkman et al. 2014 [43] | Yes | No | Yes | Yes | Good | Yes | No | Fair | Yes | Yes | Yes | Good | Fair |
| Leal et al. 2012 [44] | Yes | No | Yes | Yes | Good | Yes | No | Fair | No | Yes | Yes | Fair | Fair |
| Lee et al. 2015 [45] | Yes | No | Yes | Yes | Good | Yes | No | Fair | No | Yes | Yes | Good | Fair |
| Lewis et al. 2012 [46] | Yes | No | No | Yes | Fair | Yes | No | Fair | No | Yes | Yes | Fair | Fair |
| Lewis et al. 2015 [21] | Yes | No | Yes | Yes | Good | Yes | Yes | Good | No | Yes | Yes | Fair | Good |
| Lo et al. 1994 [S28] | Yes | Yes | Yes | Yes | Good | Yes | No | Fair | No | Yes | Yes | Fair | Fair |
| Macdonald et al. 2004 [S29] | No | Yes | No | Yes | Fair | Yes | No | Fair | No | Yes | Yes | Fair | Fair |
| Malgorzewicz et al. 2008 [S30] | Yes | No | No | Yes | Fair | Yes | No | Fair | No | Yes | Yes | Fair | Fair |
| Malgorzewicz et al. 2010 [22] | Yes | No | No | Yes | Fair | Yes | No | Fair | No | Yes | Yes | Fair | Fair |
| Malgorzewicz et al. 2018 [23] | Yes | Yes | No | Yes | Good | Yes | No | Fair | No | Yes | Yes | Fair | Fair |
| Marchelek-Mysliwiec et al. 2019 [24] | No | No | No | Yes | Poor | Yes | No | Fair | No | Yes | Yes | Fair | Poor |
| Matyjek et al. 2020 [25] | Yes | No | No | Yes | Fair | Yes | No | Fair | No | Yes | Yes | Fair | Fair |
| Medici et al. 2005 [S31] | Yes | No | Yes | Yes | Good | Yes | No | Fair | No | Yes | Yes | Fair | Fair |
| Molsted et al. 2007 [26] | Yes | No | No | Yes | Fair | Yes | Yes | Good | No | Yes | Yes | Fair | Fair |
| Moraes et al. 2013 [27] | Yes | No | Yes | Yes | Good | Yes | No | Fair | No | Yes | Yes | Fair | Fair |
| Morishita et al. 2014 [47] | Yes | Yes | No | Yes | Good | Yes | No | Fair | No | Yes | Yes | Fair | Fair |
| Navaneethan et al. 2014 [48] | Yes | No | Yes | Yes | Good | Yes | No | Fair | No | Yes | Yes | Fair | Fair |
| Nishizawa et al. 1998 [S32] | No | Yes | Yes | Yes | Good | Yes | No | Fair | No | Yes | Yes | Fair | Fair |
| O' Sullivan et al. 2002 [28] | Yes | Yes | Yes | Yes | Good | Yes | Yes | Good | No | Yes | Yes | Fair | Good |
| Ohkawa et al. 2005 [29] | Yes | No | No | Yes | Fair | Yes | No | Fair | No | Yes | Yes | Fair | Fair |
| Rayner et al. 1991 [30] | Yes | Yes | Yes | Yes | Good | Yes | No | Fair | No | Yes | Yes | Fair | Fair |
| Rodrigues et al. 2016 [49] | Yes | No | Yes | Yes | Good | Yes | No | Fair | No | Yes | Yes | Fair | Fair |
| Rymarz et al. 2016 [50] | Yes | No | Yes | Yes | Good | Yes | No | Fair | No | Yes | Yes | Fair | Fair |
| Rymarz et al. 2019 [51] | Yes | No | No | Yes | Fair | Yes | No | Fair | No | Yes | Yes | Fair | Fair |
| Sabintino et al. 2019 [52] | Yes | No | Yes | Yes | Good | Yes | No | Fair | No | Yes | Yes | Fair | Fair |
| Sakkas et al. 2003 [53] | Yes | No | Yes | Yes | Good | Yes | No | Fair | No | Yes | Yes | Fair | Fair |
| Segura-Orti et al. 2018 [54] | Yes | No | No | Yes | Fair | Yes | No | Fair | No | Yes | Yes | Fair | Fair |
| Skouroliakou et al. 2009 [55] | Yes | No | No | Yes | Fair | Yes | No | Fair | No | Yes | Yes | Fair | Fair |
| Suneja et al. 2011 [31] | Yes | Yes | No | Yes | Good | Yes | Yes | Good | No | Yes | Yes | Fair | Good |
| Tan et al. 2019 [32] | Yes | No | Yes | Yes | Good | Yes | No | Fair | No | Yes | Yes | Fair | Fair |
| 1.1= Truly representative of the average adult (>18 years old) chronic kidney disease patient in the community; 1.2= somewhat representative of the average adult (age gap <10 years) chronic kidney disease patient in the community; 1.3= controls are drawn from the same community as the chronic kidney disease patients; 1.4= determined chronic kidney disease via secure record (for example: Medical chart or they measured themselves); 2.1= study controls for muscle size measurement; 2.2= study controls for diabetes or excludes diabetes; 3.1= assessment of outcome performed via blind assessment; 3.2= assessment of outcomes performed via record linkage (took measurement, but did not blind); 3.3= follow-up was long enough for outcomes to occur | | | | | | | | | | | | | |
| **Table S3 continued. Clinical Studies (Excluding Transplant Studies) Quality Analysis via the Newcastle-Ottawa Scale (NOS)** | | | | | | | | | | | | | |
|  | **Selection** | | | | | **Comparability** | | | **Ascertainment** | | | | **Overall** |
| **Study** | **1.1** | **1.2** | **1.3** | **1.4** | **Quality** | **2.1** | **2.2** | **Quality** | **3.1** | **3.2** | **3.3** | **Quality** | **Quality** |
| Teo et al. 2014 [58] | Yes | No | Yes | Yes | Good | Yes | No | Fair | No | Yes | Yes | Fair | Fair |
| Van de ham et al. 2005 [S33] | Yes | No | No | Yes | Fair | Yes | Yes | Good | No | Yes | Yes | Fair | Fair |
| Vodicar et al. 2018 [33] | Yes | No | Yes | Yes | Good | Yes | No | Fair | No | Yes | Yes | Fair | Fair |
| Wallin et al. 2018 [57] | Yes | No | No | Yes | Fair | Yes | No | Fair | No | Yes | Yes | Fair | Fair |
| Wallin et al. 2020 [34] | Yes | No | Yes | Yes | Good | Yes | Yes | Good | No | Yes | Yes | Fair | Good |
| Wilkinson et al. 2021 [58] | Yes | Yes | Yes | Yes | Good | Yes | No | Fair | No | Yes | Yes | Fair | Fair |
| Woodrow et al. 1996 [59] | Yes | No | No | Yes | Fair | Yes | Yes | Good | No | Yes | Yes | Fair | Fair |
| Yilmaz et al. 2020 [60] | Yes | No | No | Yes | Fair | Yes | No | Fair | No | Yes | Yes | Fair | Fair |
| Young et al. 1997 [S34] | No | Yes | No | Yes | Fair | Yes | Yes | Good | No | Yes | Yes | Fair | Fair |
| Zamojska et al. 2006 [S35] | Yes | No | No | Yes | Fair | Yes | No | Fair | No | Yes | Yes | Fair | Fair |
| 1.1= Truly representative of the average adult (>18 years old) chronic kidney disease patient in the community; 1.2= somewhat representative of the average adult (age gap <10 years) chronic kidney disease patient in the community; 1.3= controls are drawn from the same community as the chronic kidney disease patients; 1.4= determined chronic kidney disease via secure record (for example: Medical chart or they measured themselves); 2.1= study controls for muscle size measurement; 2.2= study controls for diabetes or excludes diabetes; 3.1= assessment of outcome performed via blind assessment; 3.2= assessment of outcomes performed via record linkage (took measurement, but did not blind); 3.3= follow-up was long enough for outcomes to occur | | | | | | | | | | | | | |

| **Table S4. Clinical Transplant Studies Quality analysis via the Newcastle-Ottawa Scale** | | | | | | | | | | | | | |
| --- | --- | --- | --- | --- | --- | --- | --- | --- | --- | --- | --- | --- | --- |
|  | **Selection** | | | | | **Comparability** | | | **Ascertainment** | | | | **Overall** |
| **Study** | **1.1** | **1.2** | **1.3** | **1.4** | **Quality** | **2.1** | **2.2** | **Quality** | **3.1** | **3.2** | **3.3** | **Quality** | **Quality** |
| Coroas et al. 2005 [61] | Yes | No | No | Yes | Fair | Yes | Yes | Good | No | Yes | Yes | Fair | Fair |
| Marchelek-Mysliwiec et al. 2019 [24] | No | No | No | Yes | Poor | Yes | No | Fair | No | Yes | Yes | Fair | Poor |
| Matei et al. 2022 [62] | Yes | Yes | No | Yes | Good | Yes | Yes | Good | No | Yes | Yes | Fair | Good |
| Mathieu et al. 1994 [63] | Yes | Yes | No | Yes | Good | Yes | No | Fair | No | Yes | Yes | Fair | Fair |
| Qureshi et al. 1994 [64] | Yes | No | No | Yes | Fair | Yes | Yes | Good | No | Yes | Yes | Fair | Fair |
| Van de ham et al. 2005 [S33] | Yes | No | No | Yes | Fair | Yes | Yes | Good | No | Yes | Yes | Fair | Fair |
| Wong et al. 2004 [65] | Yes | No | No | Yes | Fair | Yes | No | Fair | No | Yes | Yes | Fair | Fair |
| 1.1= Truly representative of the average adult (>18 years old) chronic kidney disease patient in the community; 1.2= somewhat representative of the average adult (age gap <10 years) chronic kidney disease patient in the community; 1.3= controls are drawn from the same community as the chronic kidney disease patients; 1.4= determined chronic kidney disease via secure record (for example: Medical chart or they measured themselves); 2.1= study controls for muscle size measurement; 2.2= study controls for diabetes or excludes diabetes; 3.1= assessment of outcome performed via blind assessment; 3.2= assessment of outcomes performed via record linkage (took measurement, but did not blind); 3.3= follow-up was long enough for outcomes to occur | | | | | | | | | | | | | |

| **Table S5 continued. Preclinical Studies Quality Analysis via the SRYCLE’s Tool** | | | | | | | | | | | | | | | | | | | | | | | | | | | | | | | | |
| --- | --- | --- | --- | --- | --- | --- | --- | --- | --- | --- | --- | --- | --- | --- | --- | --- | --- | --- | --- | --- | --- | --- | --- | --- | --- | --- | --- | --- | --- | --- | --- | --- |
|  | **Sequence Bias** | | **Baseline Characteristics** | | | | **Allocation Concealment** | | **Random Housing** | | | **Performance Blinding** | | **Random Outcomes** | | **Detection Blinding** | | | **Attrition** | | | | | **Reporting Bias** | | | **Other Sources** | | | | | |
| **Study** | **1.1** | **B** | **2.1** | **2.2** | **2.3** | **B** | **3.1** | **B** | **4.1** | **4.2** | **B** | **5.1** | **B** | **6.1** | **B** | **7.1** | **7.2** | **B** | **8.1** | **8.2** | **8.3** | **8.4** | **B** | **9.1** | **9.2** | **B** | **10.1** | **10.2** | **10.3** | **10.4** | **10.5** | **B** |
| Acevedo et al. 2016 [66] | U | U | Y | ? | Y | Y | U | U | U | Y | Y | U | U | U | U | U | Y | Y | Y | ? | ? | ? | Y | N | Y | Y | Y | Y | Y | Y | N | Y |
| Adams et al. 2005 [67] | U | U | Y | ? | Y | Y | U | U | U | Y | Y | U | U | U | U | U | Y | Y | U | ? | ? | ? | U | N | Y | Y | Y | Y | U | U | N | U |
| Amann et al. 1997 [68] | U | U | Y | ? | Y | Y | U | U | U | Y | Y | U | U | U | U | Y | ? | Y | U | ? | ? | ? | U | N | Y | Y | Y | Y | Y | Y | N | Y |
| Andres-Hernando et al. 2019 [69] | N | N | N | Y | U | U | U | U | U | Y | Y | U | U | U | U | U | Y | Y | U | ? | ? | ? | U | N | Y | Y | Y | Y | U | U | U | U |
| Berru et al. 2019 [70] | N | N | U | ? | Y | U | U | U | U | Y | Y | U | U | U | U | U | Y | Y | U | ? | ? | ? | U | N | Y | Y | Y | Y | U | U | U | U |
| Cheung et al. 2020 [71] | N | N | Y | ? | Y | Y | U | U | U | U | U | U | U | U | U | U | Y | Y | U | ? | ? | ? | U | N | Y | Y | Y | Y | U | U | U | U |
| Cheung et al. 2021 [101] | N | N | Y | ? | U | Y | U | U | U | U | U | U | U | U | U | U | Y | Y | U | ? | ? | ? | U | N | Y | Y | Y | Y | U | U | U | U |
| De Moraes et al. 2018 [72] | U | U | Y | ? | Y | Y | U | U | U | Y | Y | U | U | U | U | Y | ? | Y | U | ? | ? | ? | U | N | Y | Y | Y | Y | U | U | N | U |
| Enoki et al. 2017 [73] | U | U | Y | ? | Y | Y | U | U | U | U | U | U | U | U | U | U | Y | Y | U | ? | ? | ? | U | N | Y | Y | Y | U | U | U | N | U |
| Flisinski et al. 2014 [74] | U | U | U | Y | Y | U | U | U | U | Y | Y | U | U | U | U | U | Y | Y | U | ? | ? | ? | U | N | Y | Y | Y | Y | Y | Y | N | Y |
| Hu et al. 2010 [76] | N | N | U | Y | U | U | U | U | U | U | U | U | U | U | U | U | Y | Y | U | ? | ? | ? | U | N | Y | Y | Y | Y | U | U | N | U |
| Hu et al. 2015 [100] | N | N | Y | ? | U | Y | U | U | U | U | U | U | U | U | U | U | Y | Y | U | ? | ? | ? | U | N | Y | Y | Y | Y | U | U | N | U |
| Hu et al. 2019 [75] | N | N | U | U | U | U | U | U | U | U | U | U | U | U | U | U | U | U | U | ? | ? | ? | U | N | Y | Y | Y | Y | U | U | N | U |
| Kim et al. 2021 [13] | U | U | Y | ? | Y | Y | U | U | U | Y | Y | U | U | U | U | U | Y | Y | U | ? | ? | ? | U | N | Y | Y | Y | Y | U | U | U | U |
| B=bias; U=unclear; N=no; Y=Yes; ?= N/A; 1.1= Was the allocation sequence adequately generated and applied?; 2.1= Was the distribution of relevant baseline characteristics balanced for the intervention and control groups?; If relevant, did the investigators adequately adjust for unequal distribution of some relevant baseline characteristics in the analysis?; Was timing of disease induction adequate?; 3.1= Could the investigator allocating the animals to intervention or control group not foresee assignment?; 4.1= Did the authors randomly place the cages or animals within the animal room/facility?; 4.2= Is it unlikely that the outcome or the outcome measurement was influenced by not randomly housing the animals?; 5.1= Was blinding of caregivers and investigators ensured, and was it unlikely that their blinding could have been broken?; 6.1=Did the investigators randomly pock an animals during outcome assessment, or did they use a random component?; 7.1= Was blinding of the outcome assessor ensured and was it unlikely that blinding could have been broken?; 7.2= Was the outcome assessor not blinded, but do review authors judge that the outcome is not likely to be influenced by lack of blinding?; 8.1= Were all animals included in the analysis?; 8.2=were the reasons for missing outcome data unlikely to be related to true outcome?; 8.3= are missing outcome data balanced in numbers across intervention groups with similar reasons for missing data across groups?; 8.4=are missing outcome data imputed using appropriate methods?; 9.1=was the study protocol available and were all of the study’s pre-specified primary and secondary outcomes reported in the current manuscript?; 9.2= Was the study protocol not available, but was it clear that the published reported included all expected outcomes?; 10.1=was the study free of contamination?; 10.2= Was the study free of inappropriate influence of funders; 10.3= Was the study free of unit of analysis errors?; 10.4= Were design specific risks of bias absent?; 10.5= Were new animals added to the control and experimental groups to replace dropouts from the original population? | | | | | | | | | | | | | | | | | | | | | | | | | | | | | | | | |

| **Table S5 continued. Preclinical Studies Quality Analysis via the SRYCLE’s Tool** | | | | | | | | | | | | | | | | | | | | | | | | | | | | | | | | |
| --- | --- | --- | --- | --- | --- | --- | --- | --- | --- | --- | --- | --- | --- | --- | --- | --- | --- | --- | --- | --- | --- | --- | --- | --- | --- | --- | --- | --- | --- | --- | --- | --- |
|  | **Sequence Bias** | | **Baseline Characteristics** | | | | **Allocation Concealment** | | **Random Housing** | | | **Performance Blinding** | | **Random Outcomes** | | **Detection Blinding** | | | **Attrition** | | | | | **Reporting Bias** | | | **Other Sources** | | | | | |
| **Study** | **1.1** | **B** | **2.1** | **2.2** | **2.3** | **B** | **3.1** | **B** | **4.1** | **4.2** | **B** | **5.1** | **B** | **6.1** | **B** | **7.1** | **7.2** | **B** | **8.1** | **8.2** | **8.3** | **8.4** | **B** | **9.1** | **9.2** | **B** | **10.1** | **10.2** | **10.3** | **10.4** | **10.5** | **B** |
| Liu et al. 2021 [77] | U | U | Y | ? | Y | Y | U | U | U | U | U | U | U | U | U | U | U | U | U | ? | ? | ? | U | N | Y | Y | Y | Y | U | U | N | U |
| Mitrou et al. 2017 [79] | N | N | Y | ? | U | Y | U | U | U | Y | Y | U | U | U | U | Y | ? | Y | U | ? | ? | ? | U | N | Y | Y | Y | Y | U | U | N | U |
| Mitrou et al. 2019 [78] | U | U | Y | ? | U | Y | U | U | U | U | U | Y | Y | U | U | Y | ? | Y | U | ? | ? | ? | U | N | Y | Y | Y | Y | U | U | N | U |
| Moraes et al. 2018 [80] | N | N | Y | ? | U | Y | U | U | U | Y | Y | U | U | U | U | U | Y | Y | U | ? | ? | ? | U | N | Y | Y | Y | Y | U | U | N | U |
| Niida et al. 2020 [81] | N | N | Y | ? | U | Y | U | U | U | Y | Y | U | U | U | U | U | Y | Y | U | ? | ? | ? | U | N | Y | Y | Y | Y | U | Y | N | Y |
| Organ et al. 2016 [82] | N | N | Y | ? | Y | Y | U | U | U | U | U | U | U | U | U | U | Y | Y | U | ? | ? | ? | U | N | Y | Y | Y | Y | U | U | N | U |
| Pan et al. 2019 [83] | N | N | Y | ? | U | Y | U | U | U | Y | Y | U | U | U | U | U | Y | Y | U | ? | ? | ? | U | N | Y | Y | Y | Y | U | U | N | U |
| Qian et al. 2019 [84] | U | U | Y | ? | U | Y | U | U | U | Y | Y | Y | Y | U | U | Y | ? | Y | U | ? | ? | ? | U | N | Y | Y | Y | Y | U | U | N | U |
| Sato et al. 2016 [85] | U | U | Y | ? | Y | Y | U | U | U | U | U | U | U | U | U | U | Y | Y | U | ? | ? | ? | U | N | Y | Y | Y | Y | U | U | N | U |
| Saud et al. 2021 [86] | U | U | Y | ? | Y | Y | U | U | U | Y | Y | U | U | U | U | U | Y | Y | U | ? | ? | ? | U | N | Y | Y | Y | Y | U | U | N | U |
| Solagna et al. 2021 [87] | N | N | Y | ? | U | Y | U | U | U | Y | Y | U | U | U | U | U | Y | Y | U | ? | ? | ? | U | N | Y | Y | Y | Y | U | U | N | U |
| Souza et al. 2018 [88] | U | U | Y | ? | Y | Y | U | U | U | Y | Y | U | U | U | U | U | Y | Y | U | ? | ? | ? | U | N | Y | Y | Y | Y | N | N | N | N |
| Thome et al. 2020 [89] | N | N | Y | ? | U | Y | U | U | U | Y | Y | U | U | U | U | Y | ? | Y | U | ? | ? | ? | U | N | Y | Y | Y | Y | U | U | N | U |
| Uchiyama et al. 2020 [90] | U | U | Y | ? | Y | Y | U | U | U | Y | Y | U | U | U | U | U | Y | Y | U | ? | ? | ? | U | N | Y | Y | Y | N | Y | Y | N | Y |
| Wang et al. 2018 [91] | U | U | Y | ? | Y | Y | U | U | U | Y | Y | U | U | U | U | U | Y | Y | N | Y | Y | U | Y | N | Y | Y | Y | Y | U | U | N | U |
| B=bias; U=unclear; N=no; Y=Yes; ?= N/A; 1.1= Was the allocation sequence adequately generated and applied?; 2.1= Was the distribution of relevant baseline characteristics balanced for the intervention and control groups?; If relevant, did the investigators adequately adjust for unequal distribution of some relevant baseline characteristics in the analysis?; Was timing of disease induction adequate?; 3.1= Could the investigator allocating the animals to intervention or control group not foresee assignment?; 4.1= Did the authors randomly place the cages or animals within the animal room/facility?; 4.2= Is it unlikely that the outcome or the outcome measurement was influenced by not randomly housing the animals?; 5.1= Was blinding of caregivers and investigators ensured, and was it unlikely that their blinding could have been broken?; 6.1=Did the investigators randomly pock an animals during outcome assessment, or did they use a random component?; 7.1= Was blinding of the outcome assessor ensured and was it unlikely that blinding could have been broken?; 7.2= Was the outcome assessor not blinded, but do review authors judge that the outcome is not likely to be influenced by lack of blinding?; 8.1= Were all animals included in the analysis?; 8.2=were the reasons for missing outcome data unlikely to be related to true outcome?; 8.3= are missing outcome data balanced in numbers across intervention groups with similar reasons for missing data across groups?; 8.4=are missing outcome data imputed using appropriate methods?; 9.1=was the study protocol available and were all of the study’s pre-specified primary and secondary outcomes reported in the current manuscript?; 9.2= Was the study protocol not available, but was it clear that the published reported included all expected outcomes?; 10.1=was the study free of contamination?; 10.2= Was the study free of inappropriate influence of funders; 10.3= Was the study free of unit of analysis errors?; 10.4= Were design specific risks of bias absent?; 10.5= Were new animals added to the control and experimental groups to replace dropouts from the original population? | | | | | | | | | | | | | | | | | | | | | | | | | | | | | | | | |
| **Table S5 continued. Preclinical Studies Quality Analysis via the SRYCLE’s Tool** | | | | | | | | | | | | | | | | | | | | | | | | | | | | | | | | |
|  | **Sequence Bias** | | **Baseline Characteristics** | | | | **Allocation Concealment** | | **Random Housing** | | | **Performance Blinding** | | **Random Outcomes** | | **Detection Blinding** | | | **Attrition** | | | | | **Reporting Bias** | | | **Other Sources** | | | | | |
| **Study** | **1.1** | **B** | **2.1** | **2.2** | **2.3** | **B** | **3.1** | **B** | **4.1** | **4.2** | **B** | **5.1** | **B** | **6.1** | **B** | **7.1** | **7.2** | **B** | **8.1** | **8.2** | **8.3** | **8.4** | **B** | **9.1** | **9.2** | **B** | **10.1** | **10.2** | **10.3** | **10.4** | **10.5** | **B** |
| Wang et al. 2019 [92] | U | U | Y | ? | U | Y | U | U | U | Y | Y | U | U | U | U | U | Y | Y | U | ? | ? | ? | U | N | Y | Y | Y | Y | U | U | N | U |
| Wu et al. 2019 [93] | N | N | Y | ? | U | Y | U | U | U | Y | Y | U | U | U | U | U | Y | Y | U | ? | ? | ? | U | N | Y | Y | Y | Y | U | U | N | U |
| Xia et al. 2021 [94] | U | U | Y | ? | Y | Y | U | U | U | Y | Y | U | U | U | U | U | Y | Y | U | ? | ? | ? | U | N | Y | Y | Y | Y | U | U | N | U |
| Yoshida et al. 2017 [95] | U | U | Y | ? | U | Y | U | U | U | Y | Y | U | U | U | U | U | Y | Y | N | Y | N | ? | N | N | Y | Y | Y | Y | Y | Y | N | Y |
| Zhang et al. 2015 [96] | U | U | Y | ? | Y | Y | U | U | U | Y | Y | U | U | U | U | U | Y | Y | U | ? | ? | ? | U | N | Y | Y | Y | Y | U | U | N | U |
| Zhang et al. 2019 [97] | U | U | Y | ? | Y | Y | U | U | U | Y | Y | U | U | U | U | Y | ? | Y | Y | ? | ? | ? | Y | N | Y | Y | Y | Y | U | U | N | U |
| Zhang et al. 2020 [98] | N | N | Y | ? | U | Y | U | U | U | Y | Y | U | U | U | U | U | Y | Y | U | ? | ? | ? | U | N | Y | Y | Y | N | U | U | N | U |
| Zhang et al. 2021 [99] | U | U | Y | ? | Y | Y | U | U | U | Y | Y | U | U | U | U | U | Y | Y | N | U | N | ? | N | N | Y | Y | Y | Y | N | N | N | N |
| B=bias; U=unclear; N=no; Y=Yes; ?= N/A; 1.1= Was the allocation sequence adequately generated and applied?; 2.1= Was the distribution of relevant baseline characteristics balanced for the intervention and control groups?; If relevant, did the investigators adequately adjust for unequal distribution of some relevant baseline characteristics in the analysis?; Was timing of disease induction adequate?; 3.1= Could the investigator allocating the animals to intervention or control group not foresee assignment?; 4.1= Did the authors randomly place the cages or animals within the animal room/facility?; 4.2= Is it unlikely that the outcome or the outcome measurement was influenced by not randomly housing the animals?; 5.1= Was blinding of caregivers and investigators ensured, and was it unlikely that their blinding could have been broken?; 6.1=Did the investigators randomly pock an animals during outcome assessment, or did they use a random component?; 7.1= Was blinding of the outcome assessor ensured and was it unlikely that blinding could have been broken?; 7.2= Was the outcome assessor not blinded, but do review authors judge that the outcome is not likely to be influenced by lack of blinding?; 8.1= Were all animals included in the analysis?; 8.2=were the reasons for missing outcome data unlikely to be related to true outcome?; 8.3= are missing outcome data balanced in numbers across intervention groups with similar reasons for missing data across groups?; 8.4=are missing outcome data imputed using appropriate methods?; 9.1=was the study protocol available and were all of the study’s pre-specified primary and secondary outcomes reported in the current manuscript?; 9.2= Was the study protocol not available, but was it clear that the published reported included all expected outcomes?; 10.1=was the study free of contamination?; 10.2= Was the study free of inappropriate influence of funders; 10.3= Was the study free of unit of analysis errors?; 10.4= Were design specific risks of bias absent?; 10.5= Were new animals added to the control and experimental groups to replace dropouts from the original population? | | | | | | | | | | | | | | | | | | | | | | | | | | | | | | | | |
