# Supplementary material for: Skeletal muscle atrophy in clinical and preclinical models of chronic kidney disease: A systematic review and meta‐analysis
Source: J Cachexia Sarcopenia Muscle. 2023 Dec 7;15(1):21–35. doi: 10.1002/jcsm.13400 (PMC10834351; doi:10.1002/jcsm.13400)
Supplement: Supplementary file 4 — Data S4. Supporting Information. [file JCSM-15-21-s001.docx]

**Supplementary References**

1. Workeneh BT, Mitch WE. Review of muscle wasting associated with chronic kidney disease. The American Journal of Clinical Nutrition. 2010;91:1128S-32S.1.
2. Wang XH, Mitch WE. Mechanisms of muscle wasting in chronic kidney disease. Nature Reviews Nephrology. 2014;10:504-16.
3. Martinson M, Ikizler TA, Morrell G, Wei G, Almeida N, Marcus RL, et al. Associations of body size and body composition with functional ability and quality of life in hemodialysis patients. Clinical Journal of the American Society of Nephrology. 2014;9:1082-90.
4. Carrero JJ, Chmielewski M, Axelsson J, Snaedal S, Heimbürger O, Bárány P, et al. Muscle atrophy, inflammation and clinical outcome in incident and prevalent dialysis patients. Clinical Nutrition. 2008;27:557-64.
5. Higgins JPT, Green S, Cochrane Collaboration. Cochrane handbook for systematic reviews of interventions. Chichester, England ; Hoboken, NJ: Wiley-Blackwell; 2011.
6. Zeng X, Zhang Y, Kwong JSW, Zhang C, Li S, Sun F, et al. The methodological quality assessment tools for preclinical and clinical studies, systematic review and meta-analysis, and clinical practice guideline: a systematic review. Journal of Evidence-Based Medicine. 2015;8:2-10.
7. Moreno-Rabie C, Gaeta-Araujo H, Oliveira-Santos C, Politis C, Jacobs R. Early imaging signs of the use of antiresorptive medication and MRONJ: a systematic review. Clin Oral Investig. 2020;24:2973-89.
8. Hooijmans CR, Rovers MM, de Vries RBM, Leenaars M, Ritskes-Hoitinga M, Langendam MW. SYRCLE’s risk of bias tool for animal studies. BMC Medical Research Methodology. 2014;14:43.
9. Wells GA, Shea B, O’Connell D, Peterson J, Welch V, Losos M, et al. The Newcastle-Ottawa Scale (NOS) for assessing the quality of nonrandomised studies in meta-analyses. Oxford; 2000.
10. Higins JP GS, editor(s). Cochrane Handbook for Systematic Reviews of Interventions. The Cochrane Collaboration 2011.
11. Lin L, Aloe AM. Evaluation of various estimators for standardized mean difference in meta-analysis. Stat Med. 2021;40:403-26.
12. Leppink J, O'Sullivan P, Winston K. Effect size - large, medium, and small. Perspect Med Educ. 2016;5:347-9.
13. Asbaghi O, Sadeghian M, Rahmani S, Mardani M, Khodadost M, Maleki V, et al. The effect of green coffee extract supplementation on anthropometric measures in adults: A comprehensive systematic review and dose-response meta-analysis of randomized clinical trials. Complement Ther Med. 2020;51:102424.
14. Williams R. Moderateor analyses: Categorical models and Meta-regression https://campbellcollaboration.org/images/pdf/plain-language/Moderator_Analysis_Williams.pdf: The Campbell Collaboration
15. Higgins JP TJ, Chandler J, Cumpston M, Li T, Page MJ, Welch VA, editor(s). Cochrane Handbook for Systematic Reviews of Interventions Available from training.cochrane.org/handbook: Cochrane; 2021.
16. Bazanelli AP, Kamimura MA, da Silva CB, Avesani CM, Lopes MG, Manfredi SR, et al. Resting energy expenditure in peritoneal dialysis patients. Perit Dial Int. 2006;26:697-704.
17. Boivin MA, Battah SI, Dominic EA, Kalantar-Zadeh K, Ferrando A, Tzamaloukas AH, et al. Activation of caspase-3 in the skeletal muscle during haemodialysis. Eur J Clin Invest. 2010;40:903-10.
18. Chen YC, Chen HH, Yeh JC, Chen SY. Body composition in hemodialysis patients--is it different from that of normal subjects? Clin Nephrol. 2000;53:291-5.
19. Crowe AV, McArdle A, McArdle F, Pattwell DM, Bell GM, Kemp GJ, et al. Markers of oxidative stress in the skeletal muscle of patients on haemodialysis. Nephrol Dial Transplant. 2007;22:1177-83.
20. Cupisti A, Licitra R, Chisari C, Stampacchia G, D'Alessandro C, Galetta F, et al. Skeletal muscle and nutritional assessment in chronic renal failure patients on a protein-restricted diet. J Intern Med. 2004;255:115-24.
21. Diesel W, Emms M, Knight BK, Noakes TD, Swanepoel CR, van Zyl Smit R, et al. Morphologic features of the myopathy associated with chronic renal failure. Am J Kidney Dis. 1993;22:677-84.
22. Elsayed NM, Hamed ST, El-Khatib MM, El-Shehaby AM. The relation between dual energy x-ray absorptiometry measurement of body fat composition and plasma ghrelin in patients with end-stage renal disease. Saudi Med J. 2009;30:109-15.
23. Guida B, De Nicola L, Pecoraro P, Trio R, Di Paola F, Iodice C, et al. Abnormalities of bioimpedance measures in overweight and obese hemodialyzed patients. Int J Obes Relat Metab Disord. 2001;25:265-72.
24. Jairam A, Das R, Aggarwal PK, Kohli HS, Gupta KL, Sakhuja V, et al. Iron status, inflammation and hepcidin in ESRD patients: The confounding role of intravenous iron therapy. Indian J Nephrol. 2010;20:125-31.
25. Johansen KL, Mulligan K, Tai V, Schambelan M. Leptin, body composition, and indices of malnutrition in patients on dialysis. J Am Soc Nephrol. 1998;9:1080-4.
26. Kamimura MA, Draibe SA, Avesani CM, Canziani ME, Colugnati FA, Cuppari L. Resting energy expenditure and its determinants in hemodialysis patients. Eur J Clin Nutr. 2007;61:362-7.
27. Kemp GJ, Crowe AV, Anijeet HK, Gong QY, Bimson WE, Frostick SP, et al. Abnormal mitochondrial function and muscle wasting, but normal contractile efficiency, in haemodialysed patients studied non-invasively in vivo. Nephrol Dial Transplant. 2004;19:1520-7.
28. Lo WK, Prowant BF, Moore HL, Gamboa SB, Nolph KD, Flynn MA, et al. Comparison of different measurements of lean body mass in normal individuals and in chronic peritoneal dialysis patients. Am J Kidney Dis. 1994;23:74-85.
29. Macdonald JH, Phanish MK, Marcora SM, Jibani M, Bloodworth LL, Holly JM, et al. Muscle insulin-like growth factor status, body composition, and functional capacity in hemodialysis patients. J Ren Nutr. 2004;14:248-52.
30. Malgorzewicz S, Debska-Slizien A, Rutkowski B, Lysiak-Szydlowska W. Serum concentration of amino acids versus nutritional status in hemodialysis patients. J Ren Nutr. 2008;18:239-47.
31. Medici G, Mussi C, Fantuzzi AL, Malavolti M, Albertazzi A, Bedogni G. Accuracy of eight-polar bioelectrical impedance analysis for the assessment of total and appendicular body composition in peritoneal dialysis patients. Eur J Clin Nutr. 2005;59:932-7.
32. Nishizawa Y, Shoji T, Tanaka S, Yamashita M, Morita A, Emoto M, et al. Plasma leptin level and its relationship with body composition in hemodialysis patients. Am J Kidney Dis. 1998;31:655-61.
33. van den Ham EC, Kooman JP, Schols AM, Nieman FH, Does JD, Franssen FM, et al. Similarities in skeletal muscle strength and exercise capacity between renal transplant and hemodialysis patients. Am J Transplant. 2005;5:1957-65.
34. Young GA, Woodrow G, Kendall S, Oldroyd B, Turney JH, Brownjohn AM, et al. Increased plasma leptin/fat ratio in patients with chronic renal failure: a cause of malnutrition? Nephrol Dial Transplant. 1997;12:2318-23.
35. Zamojska S, Szklarek M, Niewodniczy M, Nowicki M. Correlates of habitual physical activity in chronic haemodialysis patients. Nephrol Dial Transplant. 2006;21:1323-7.
36. Agarwal R, Bills JE, Light RP. Diagnosing obesity by body mass index in chronic kidney disease: an explanation for the "obesity paradox?". Hypertension. 2010;56:893-900.
37. Axelsson J, Bergsten A, Qureshi AR, Heimburger O, Barany P, Lonnqvist F, et al. Elevated resistin levels in chronic kidney disease are associated with decreased glomerular filtration rate and inflammation, but not with insulin resistance. Kidney Int. 2006;69:596-604.
38. Serrano N, Colenso-Semple LM, Lazauskus KK, Siu JW, Bagley JR, Lockie RG, et al. Extraordinary fast-twitch fiber abundance in elite weightlifters. PLoS One. 2019;14:e0207975.
